# Protein Fragments: Functional and Structural Roles of Their Coevolution Networks

**DOI:** 10.1371/journal.pone.0048124

**Published:** 2012-11-05

**Authors:** Linda Dib, Alessandra Carbone

**Affiliations:** 1 Université Pierre et Marie Curie, UMR 7238, Équipe de Génomique Analytique, Paris, France; 2 CNRS, UMR 7238, Laboratoire de Génomique des Microorganismes, Paris, France; University of Cyprus, Cyprus

## Abstract

Small protein fragments, and not just residues, can be used as basic building blocks to reconstruct networks of coevolved amino acids in proteins. Fragments often enter in physical contact one with the other and play a major biological role in the protein. The nature of these interactions might be multiple and spans beyond binding specificity, allosteric regulation and folding constraints. Indeed, coevolving fragments are indicators of important information explaining folding intermediates, peptide assembly, key mutations with known roles in genetic diseases, distinguished subfamily-dependent motifs and differentiated evolutionary pressures on protein regions. Coevolution analysis detects networks of fragments interaction and highlights a high order organization of fragments demonstrating the importance of studying at a deeper level this structure. We demonstrate that it can be applied to protein families that are highly conserved or represented by few sequences, enlarging in this manner, the class of proteins where coevolution analysis can be performed and making large-scale coevolution studies a feasible goal.

## Introduction

Coevolving residues in a protein structure correspond to groups of residues whose mutations have arisen simultaneously during the evolution of different species, and this is due to several possible reasons involving the three-dimensional shape of the protein: functional interactions, conformational changes and folding.

Several studies addressed the problem of extracting signals of coevolution between residues. Two classes of methods have been developed to identify residue correlations. They exploit information coming either from the protein structure [1]–[3] or from the sequence alignment. The second class of methods investigates evolutionary constraints in protein families via the analysis of correlated distribution of amino acids in sequences and it is characterized by statistical and combinatorial approaches. Statistical methods use correlation coefficients [4], [5], mutual information [6]–[11] and deviance between marginal and conditional distributions to estimate the thermodynamic coupling between residues [12]–[15]. Phylogenetic information has been integrated [16]–[18] to help the treatment of sequences displaying the same level of co-variation. These methods ask for high sequence divergence on several positions of the sequence alignment, and require sufficiently many sequences to belong to the alignment (to guarantee statistical equilibrium [13]). In general, these constraints limit the domain of applicability. A combinatorial approach based on phylogenetic reconstructions of protein families was proposed in [19] where no filtering of sequences was required to perform the analysis and a variable divergence of protein families is accepted. The method can detect residues that are both coevolving and conserved.

All these methods provide sets of coevolved residues that are usually close in the three-dimensional structure, form connected networks covering roughly a third of the entire structure, and have been demonstrated for a few protein complexes (for which experimental data was available) to play a crucial role in allosteric mechanisms [12], [20], to maintain short paths in network communication and to mediate signaling [2], [3].

All methods have been tested on a handful of divergent protein sequences. An attempt to large-scale investigation of residue networks has been made in [16] but the class of sequences handled by the approach is filtered on criteria excluding positions that contain a high number of gaps, that are highly conserved or that are highly divergent. This brought the large-scale coevolution analysis of the PFAM database to consider 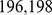 position pairs against the 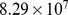 existing ones and certain families to be excluded by the analysis. In particular, 7719 Pfam domains over 12273 (version v25, where for each family of aligned sequences we eliminated 100% identical sequences) show at least 50% of their positions that are either highly gapped (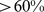 of gaps) or highly conserved (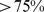 of sequences contain the same residue), and 5868 Pfam families contain less than 120 sequences, a rough lower bound for applying statistical methods of co-evolution analysis [13] (Text S1). The development of conceptually new approaches treating non divergent sequences and protein families represented by small sets of sequences reveals to be necessary for large-scale calculations [21]. To overcome this difficulty, we propose a new combinatorial method, named *Blocks In Sequences* (BIS), that detects similarities in the evolutionary behavior of alignment positions within either small or conserved sets of sequences. Contrary to statistical approaches and other combinatorial approaches, BIS does not require sequence variability nor sequence divergence, conditions that are not satisfied by the classes of sequences it addresses. It uses a counting formula that captures positional differences in aligned protein sequences and based on those it evaluates whether two or more positions underwent simultaneous mutations, that is whether they coevolved or not.

With BIS, we extend the search of coevolving positions to *blocks* of contiguous positions in sequence alignments, where a block is possibly constituted by a single position. Intuitively, blocks represent protein fragments and we determine whether fragments coevolve or not and at which strength. The concept of fragment as an elementary unit was first introduced in [22] to study protein folding, and it was exploited in several further studies [23]–[28]. Here we use the notion of fragment as an elementary unit to study protein evolution, where structural conditions are a possible evolutionary constraint. The basic observation is that coevolving positions are usually not isolated along the sequence and that their coevolution concerns their adjacent positions as well. Based on fragments, new accurate structural and functional insights on proteins can be provided. We demonstrate that coevolving fragments are indicators of important information explaining folding intermediates, distinguished subfamily-dependent functional motifs, key mutations in genetic diseases, peptide assembly, structural features and specific evolutionary pressures that have been undergoing on non-overlapping regions of the protein sequence. This suggests that the nature of coevolution signals extends beyond the role of coevolving residues in interaction sites, allosteric movements and folding as highlighted in seminal works.

To illustrate, in multiple ways, the importance of thinking about fragments while studying protein evolution, we have chosen four protein families. The Amyloid beta peptide presents predicted coevolving fragments that correspond to high affinity regions involved in peptide aggregation and predicted residues that correspond to known key mutations in Alzheimer's disease. The B domain of Protein A displays predicted sites that strikingly match fragments and residues known to play a major role in intermediate folding states; these fragments have been previously experimentally identified by 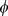-analysis. The MukB family shows that gap insertion within a protein family is an important event that can be used to extract fragment information and to explain the joint functional and structural role of different parts of a protein sequence. It highlights the possibility to identify structural features and specific evolutionary pressures undergoing non-overlapping regions, named *partitions*, of the protein sequence. Finally, the AATPase family shows that coevolution analysis can fruitfully revisit already treated protein families and bring new biological insights into play: it highlights distinguished subfamily-dependent motifs, suggests extensions of known motifs, identifies new motifs and provides evidence of a mutual evolution among motifs. For these four protein families, the number of sequences is quite restraint, varying between a few hundreds down to a dozen (Table 1 and Text S10). The percentage of identity is high: greater than 80% for the first three families and varying from 47% to 65% for the AATPase family (Fig. S1). Because of the special characteristics of the sequences, coevolution signals within these protein families are expected to be particularly difficult to extract with current methods. We begin by introducing the method on a specific example and discuss afterwards the results obtained on the four protein families.

**Table 1:** Performance of BIS coevolution analysis on AATPase families.

	Seq	API	Pos	#Exp	#Coev	TP	Prob	Sen*	Spe*	Acc*	PPV*
**Upf1**	18	0.58	677	64	26	18	2.93 *e* ^−14^	0.28	0.99	0.92	0.69
**RecD**	6	0.51	642	55	73	32	1.63 *e* ^−19^	0.58	0.93	0.90	0.44
**UvrD/Rep**	8	0.53	661	62	38	30	1.89 *e* ^−27^	0.48	0.99	0.94	0.79
**Rad3**	9	0.52	592	55	62	28	3.53 *e* ^−16^	0.51	0.94	0.90	0.45
**DEAD-box**	67	0.61	624	66	16	16	4.11 *e* ^−17^	0.24	1	0.92	1
**RecQ**	9	0.61	514	65	42	31	1.26 *e* ^−22^	0.48	0.98	0.91	0.74
**Ski2-like**	13	0.51	686	62	35	22	1.47 *e* ^−16^	0.35	0.98	0.92	0.63
**RigI-like**	6	0.47	658	64	104	40	9.42 *e* ^−20^	0.63	0.89	0.87	0.38
**DEAH-RHA**	24	0.65	562	63	43	29	8.06 *e* ^−21^	0.46	0.97	0.91	0.67
**NS3-NPH-II**	11	0.6	479	62	101	36	4.83 *e* ^−12^	0.58	0.84	0.81	0.36
**Swi2-Snf2**	45	0.51	714	76	15	14	1.05 *e* ^−13^	0.18	1	0.91	0.93

Percentage of identity for the alignment (API), number of sequences (Seq), alignment length (where all gapped positions are eliminated; Pos), number of experimentally confirmed residues (#Exp), number of residues identified by coevolution analysis (#Coev), number of true positives (TP) computed by intersecting #Exp and #Coev, probability of predicting TP residues out of #Exp by selecting #Coev residues within Len residues (Prob), Sensitivity (Sen*), Specificity (Spe*), Accuracy (Acc*), Positive Predictive Value (PPV*) are given (see Methods). Experimentally validated residues (#Exp) are those belonging to known motifs described in [56]. See Text S10.

## Results

### A Combinatorial Mapping of Fragments in Proteins

**Figure 1:**
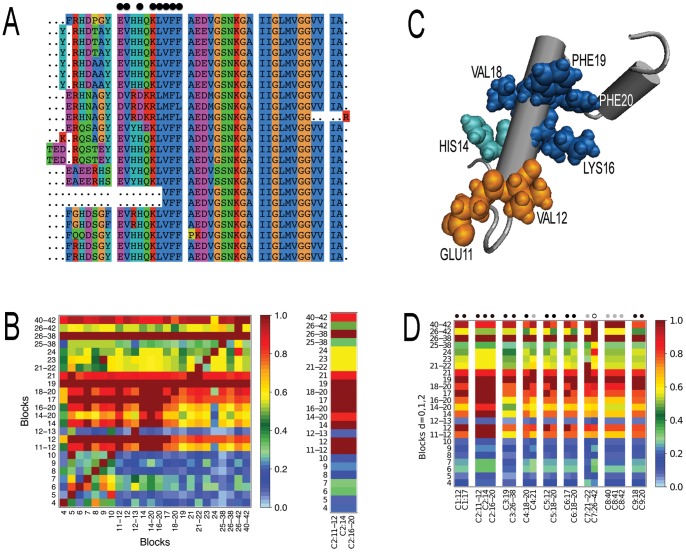
Amyloid fragment 16–20 and its coevolving blocks. A. Subset of the full sequence alignment (made of 80 members, with 87% sequence identity) used in coevolution analysis of the Amyloid family. A block is highlighted by consecutive dots (top). It corresponds to cluster C2 in BC. B. Matrix of coevolution scores between blocks of dimensions 0, 1, 2 (left) and coevolution score matrix of cluster C2 (right), where the color scale for coevolution scores goes from dark red (maximal coevolution) to pale blue (no coevolution). For C2 coevolution score matrix, notice the high coevolution scores between the three blocks in C2 (dark red) and the same coevolution scores of these blocks with all other blocks (rows are uniformly colored). Observe that the three coevolving blocks display maximal coevolution scores with block 26–38 (dark red), but that 26–38 is not claimed to coevolve with them because it displays different coevolution scores with the full set of blocks. C. Blocks 16–20, 11–12 and 14 form a network of interacting residues in the structure (1aml). D. Clusterized matrix of coevolving blocks for the Amyloid beta peptide. The matrix is computed from the unclustered matrix in B. Block dimension is indicated by a colored dot on the top: black (dimension 0), grey (dimension 1), white (dimension 2). Clusters C1–C3, C8, C9 correspond to the highest coevolution scores as shown by the uniform behavior of the blocks within each cluster. Clusters C8 and C9 are formed by the hits in blocks 40–42 and 18–20, that, by definition, display the same behavior with all other blocks. Clusters C4 and C7 are formed by blocks detected in two dimensions. Notice that C2 in B (right) is included in this matrix.

We study coevolution of blocks as we would study coevolution of positions by analyzing words variability in adjacent alignment positions instead of residues variability in a column alignment. A block is defined by extending a position, called the *hit* of the block, with adjacent positions on its right and on its left, by ensuring that the word distribution for the block is the same as the amino acids distribution for the hit. This means that, by looking at the columns corresponding to the block within the alignment, the aligned sequences where the same word appears should display the same amino acid in the hit column, and vice versa, by looking at the hit column, the aligned sequences where the same amino acid appears should display the same words in the block columns. As an example, take the alignment of the Amyloid beta (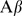) peptide fragment 16–20 reported in Fig. 1A and consider position 16, containing Arginines (K), Lysines (R) or indels (.). By extending position 16 on the right to the fragment 16–20 we observe three words KLVFF, RLMFL and ..VFF, extending K, R and. respectively, that preserve the distribution of position 16. Positions 16–20 are such that by extending them further to the left or to the right, we would increase the number of distinguished words (because of a Glutamate in position 15 and of a Proline in position 21) and change, in this way, the word distribution of the hit at position 16. We say that the extension 16–20 is maximal and call it a block.

Coevolution analysis looks for blocks in the alignment that coevolve with fragment 16–20. These blocks are identified along the alignment by extension from a position as above. They can have arbitrary size. For each block, we ask the words appearing on it to have at least two occurrences in the record of the protein family. Intuitively, we trust the analysis of coevolution signals for a block only when it is issued from alignment sequences that contain words witnessed by at least two sequences. But a block might have 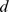 words that appear only once and in this case, we say that the block has 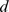 exceptions. When considering blocks of 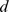 exceptions, we will ignore from coevolution analysis the 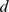 sequences with only one word occurrence. Notice that a block has 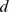 exceptions only when its hit (that is, the position from which it is extended) has 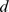 exceptions, and that the maximum value of 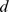 can be 21, that is the number of different amino acids together with the indel. Also, notice that blocks, possibly with the same number of exceptions, can overlap each other.

As all blocks are defined, we look for coevolution signals between pairs of blocks of at most 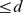 exceptions, where 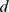 is a family dependent value. The intuition is that divergent families will have greater 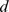 than more conserved ones. For the 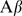 peptide, a very conserved protein family, we consider all blocks with 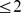 exceptions and compute coevolution scores between pairs of these blocks. The approach is combinatorial in nature and it is based on a counting argument that captures all changes in word distribution between pairs of blocks: we check within sequences preserving a word for a block, whether words vary for the other block or not. Block 16–20 turns out to coevolve with blocks 11–12 and 14 (Fig. 1A), and this can easily be seen by observing that the word EV in 11–12 changes in DV and. exactly when word H in 14 changes into D and. and when KLVFF changes into RLMLF and. VFF. It is an example of perfect coevolution between blocks. To study less regular word matching between blocks, the method considers the evolutionary pressure involving the entire 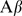 sequences, and not only the mutational process involving single positions. For this, it uses the distance tree of sequences as a representation of the evolutionary process, it extracts information from the topology of the tree and it provides numerical values evaluating the strength of coevolution between blocks (Fig. 1B). The counting argument occurring in the correlation formula (see Methods) joins together the analysis of all subtrees of the distance tree of sequences: for each subtree and each pair of blocks, the word distributions associated to the pair of blocks are compared and weighted by the size, that is the number of sequences, of the subtree.

The set of coevolving scores among all pairs of blocks gives rise to a coevolution score matrix (Fig. 1B left). We cluster this matrix to identify those blocks that display the same evolutionary behavior, that is the same maximal coevolution scores among each other, as cluster C2 in Fig. 1B (right). Intuitively, clusters should help to interpret blocks functional role. To further analyze the functional relationships between blocks in the evolutionary record of the Amyloid protein family, a mapping of clusters of coevolving blocks in the three-dimensional structure helps to display their spatial proximity, if any, and to detect their physical interactions. Fragment 16–20 is physically connected in a pathway of tight interactions with 11–12 and 14. The three blocks form a network, where a very small atomic distance (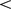
4Å) exist between at least one atom in each block of the network (Fig. 1C).

### Genetic Signals and Functional Motifs Detected by Fragments in Amyloid Beta Peptide

The complete calculation of block coevolution for the 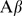 peptide provides many insights on this peptide known to be the primary reason of extracellular deposits in Alzheimer's disease [29], [30]: 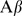 is a 43 amino acids peptide formed after sequential cleavage of the Amyloid Precursor Protein (APP) and is found in the brain of patients. To see this, we consider residues (11 17 21 22) and peptide fragments (25–35, 18–20, 16–20, 14–23, 40–42) that have been experimentally studied and highlighted to have severe clinical implications:

1. mutations on residues 11 and 17 can lead to Alzheimer's disease by shifting the cleavage site in APP [31], [32];
2. E22Q mutation (Dutch type) produces severe cerebral amyloid angiopathy. It likely affects processing of APP, leading to the formation of amyloid plaques in brain tissue and in walls of blood vessels serving the brain [33]. It is associated to stroke [34].
3. A21G mutation (Flemish type) produces cerebral amyloid angiopathy with milder effects than E22Q [33], [35]. It is associated to stroke and dementia [34].
4. several *in vitro* studies showed that peptides containing the 25–35 fragment induce neurotoxicity in neuronal cultures and self-aggregate as 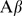 [36]–[39]. *In vivo* studies demonstrated that 25–35 administration induces amnesia in rats [40].
5. fragment 25–38 is key for the APP transmembrane sequence dimerization via the GxxxG motifs [41].
6. a study of several amnestic peptides showed that impairment was dependent on the presence of the configuration VFF in position 18–20 [42].
7. fragment 16–20 is the 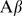 region that most efficiently binds to 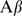, and this sequence is necessary for fibril formation [43]. The extension 14–23 was shown to be the minimal segment of 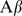 that is sufficient for fibril formation [44].
8. fragment 40–42 is the site of 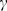-secretase cleavage, either between residues 40 and 41 or between 42 and 43; both forms 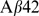 and 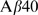 are normally present, with 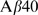 in great excess of 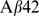 [45].

We compared these residues and fragments with those detected by coevolution analysis (run on 80 sequences). There are nine coevolving clusters: C1 =  (12 17), C2 =  (11–12 14 16–20), C3 = (19 26–38), C4 = (21 18–20), C5 = (12 18–20), C6 = (17 18–20), C7 = (21–22 26–42), C8 = (40 41 42), C9 = (18 20) (Fig. 1D), whose blocks form physically connected networks of interacting residues. These clusters sharply agree with the experimental observations reported above. Residues 11, 17, 21 and 22 are detected as coevolving residues within several clusters and their mutations are known to lead to Alzheimer's disease (observations 1–3). Among fragments identified experimentally, C3 contains 26–38 and C7 contains 26–42 that strongly overlap with 25–35 known to be the site that best binds to 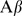 (observation 4) and with 25–38 involved in APP dimerization (observation 5). Fragment VFF (observation 6) is found to coevolve with 12, 17 and 21 in C4, C5, C6, and fragment 16–20 (observation 7) with residues 11, 12 and 14 in C2. Notice also that fragment 14–20 is identified as a block of positions submitted to the same evolutionary pressure and this supports that fragment 14–23 has been found to be the shortest fibril-forming 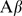-sequence containing 16–20 [46]. Finally, residues in fragment 40–42 are found to coevolve together in a cluster (observation 8). To statistically evaluate the accuracy of the predictions, we computed the probability to find 22 (experimentally validated predicted) residues over 25 (experimental residues) by selecting 27 (predicted total) residues on a pool of 43 (that is, the alignment length) and obtained 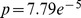 (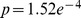, if the length of the peptide structure, that is 42 residues, is considered instead). The prediction has been realized with high accuracy (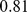), specificity (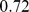), sensitivity (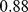) and positive predictive value (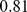). Notice that this evaluation underestimates the prediction though since it neglects fragments organization that matches well with experimental evidence.

### Fragments, Gaps and Conserved Functional Motifs: The Walker-A Motif

**Figure 2:**
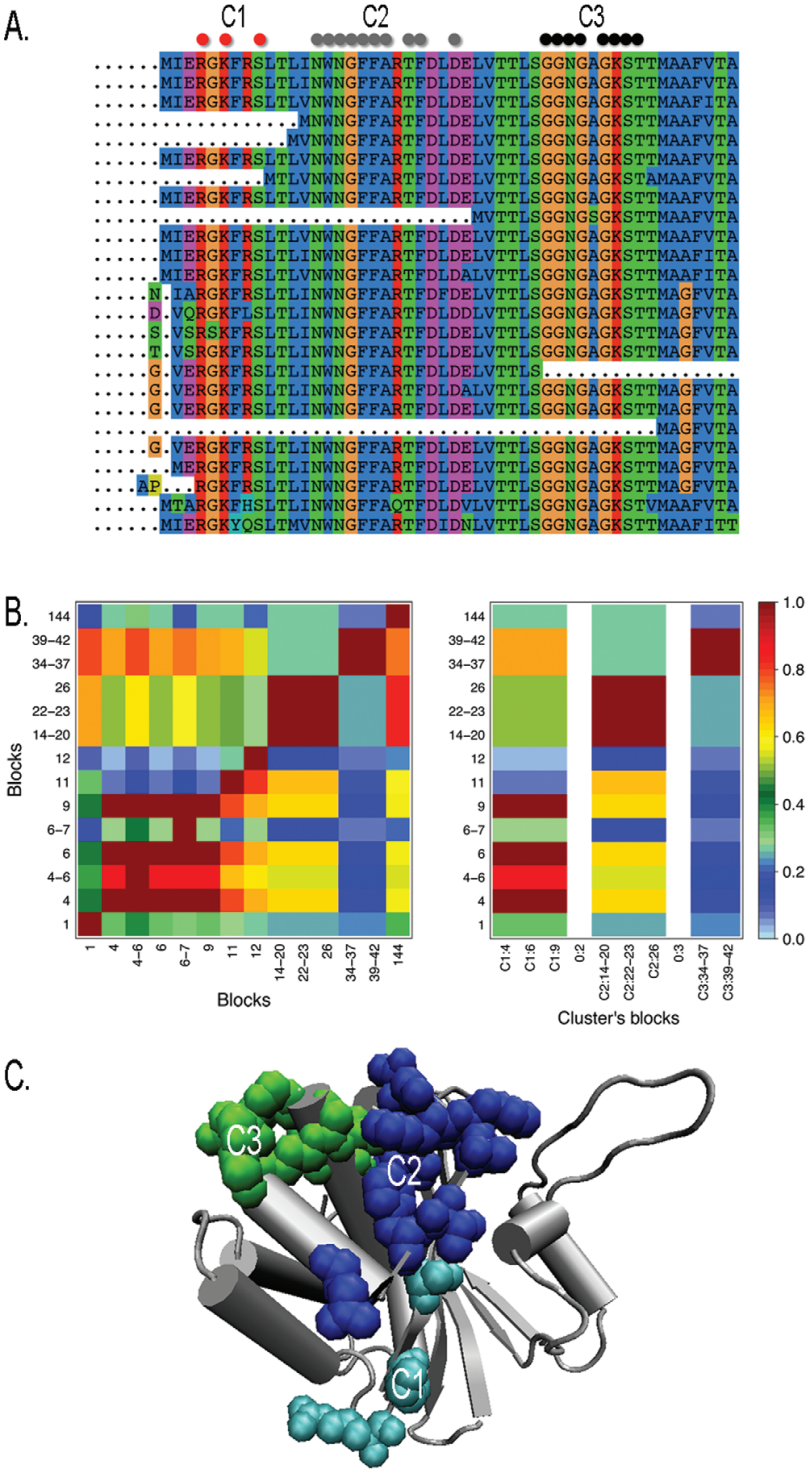
The Walker-A motif in MukB proteins. Display of a subset of the full sequence alignment (made of 200 members, with 84% sequence identity) used to analyze coevolution in the MukB family. Sequences are truncated on the right. Three clusters are indicated by colored dots (top). B. Matrix of coevolution scores between blocks of dimension 0 (left) and clustered coevolution score matrix highlighting 3 resulting clusters (right). See Text S15. C. Clusters C1–C3 are plot in the structure (1qhl:A). Walker-A (C3) is colored green.

The explicit treatment of indels as residues discussed for the 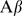 peptide, allows the sequence-based combinatorial method to successfully analyze conserved blocks besides coevolving ones in a highly accurate manner. As a challenging example, we consider an alignment of 200 members of the 26 kDa N-terminal domain of the MukB family (see Methods) which features a mixed 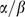-fold with a central six-stranded anti-parallel 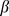-sheet and a putative Walker-A motif. The MukB protein family is highly conserved (84% sequence identity) (Fig. 2A). From the alignment, fourteen blocks are detected as being *fully conserved*, that is they are defined by a single word and possibly several gaps (that is, words made of indels only). By taking into consideration the location of gap insertions (that for different blocks might involve different sequences), the analysis discriminates eight of these blocks to cluster within 3 distinct groups with highest scores (Fig. 2B right). Among them, there are two blocks that characterize the 8-letters Walker-A motif 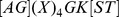. They are identified by blocks 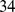
–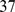 and 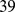
–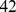 (in the pdb 1qhl), and they correspond to the subsequences 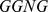 and 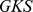 matching the Walker-A submotifs 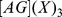 and 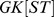. These two blocks form a network of spatially connected residues (C3 in Fig. 2C) and sharply identify the Walker-A motif. The six remaining blocks are all located on the left hand side of the Walker-A along the sequence, and form two distinguished clusters of blocks, C1 and C2, belonging to the dimerization site of the protein structure (Fig. 2BC).

The identification of the Walker-A motif challenged several prediction systems based on conservation signals. The specificity of these systems is usually not very high [47] compared to the one reached by our analysis. When run on their own datasets of homologous sequences, iJET [48] detects the Walker-A site within 29 predicted conserved residues, Consurf [49] detects it within 58, siteFINDER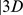 [47] within 45, and ET Viewer 2.0 [50], [51] fails to make a useful prediction. In fact, these systems output conserved clustered patches that might have several functional activities, and in particular, the residues lying in the same face of the molecule as the Walker-A are suggested to be involved in dimerization of MukB.

Taking into account that the Walker-A is predicted as forming a separate cluster, its prediction is realized with 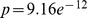, accuracy 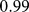, specificity 1, sensitivity 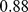 and positive predictive value at 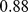.

### Clusters of Fragments are Structurally Organized in MukB

**Figure 3:**
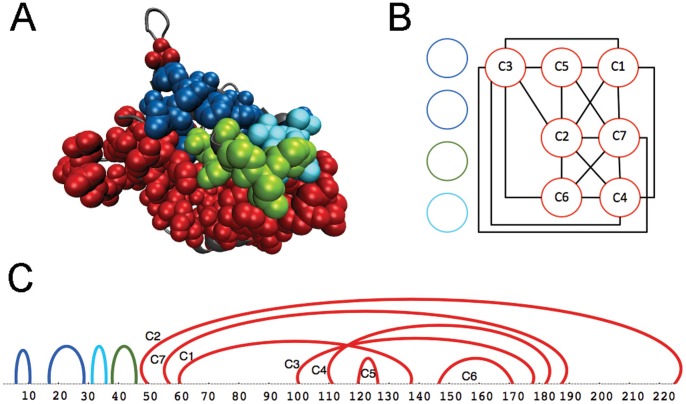
Partitions in protein MukB (*Escherichia coli K12*). Coevolution analysis, realized on a dataset of 200 sequences, detects 11 clusters at dimensions 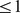. The dataset is the same as the one used in Fig. 2. A: MukB structure (1qhl:A) with coevolving clusters: in green, the Walker-A motif; in blue, clusters C1, C2 and C8 that sit on the left hand side of the Walker-A motif; in red, all clusters sitting on the right hand side of the Walker-A motif. B: interval graph of C. It is constructed by setting each interval in C to be a node of the graph and by defining an edge between two nodes when the corresponding intervals overlap or cross each other. A connected component (red) forms a partition located on the bottom of the structure in A. C: protein sequence corresponding to the structure in A (SI Text S3 and Text S15); arcs highlight intervals along the sequence hosting clusters, where an interval associated to a cluster is identified (with an arc) by using the smallest and the largest positions among all blocks in the cluster. Color code as in A. The 11 clusters have been obtained by imposing very stringent parametric conditions to coevolution analysis. More relaxed analysis conditions (with blocks defined for larger dimensions 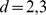, and clustering done with larger 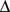 values; see Methods) still detect the Walker-A and partitions remain located on its left and right hand sides.

Do clusters of coevolving blocks organize in a structure providing evolutionary insights in fragment interactions? To highlight the existence of such a structure, we look at contiguous regions in both the sequence and the three-dimensional structure that appear to have coevolved together in the record of MukB sequences. The full MukB coevolution analysis identifies 8 clusters with 1 exception (Text S2) that are slightly less conserved than the Walker-A motif and the two clusters coevolving with it (C1 and C2, detected with no exceptions) discussed above. Among them, four are obtained by extending a non-fully conserved hit with fully conserved positions and they overlap through their fully conserved positions. The localization of all clusters along the sequence is illustrated in Fig. 3C, where the corresponding intervals are identified by arcs. Two intervals can overlap or be included one into the other, and this might happen because either the clusters share common residues or they are formed by blocks that are intercalating. By constructing the associated interval graph (Figs. 3B), we notice that the eleven clusters form five connected components in the graph. We call the connected subgraphs *partitions* of MukB: one is the Walker-A motif, one is located at the right hand side of the Walker-A motif and the remaining three on its left hand side. These partitions correspond to a spatial division of the three-dimensional structure forming a sandwich-like structural arrangement around the Walker-A motif (Fig. 3A and Text S3). This suggests that the protein sequence underwent three independent evolutionary processes: one for the Walker-A motif, and the other two for the right and the left hand side, respectively. To support this hypothesis, it is worth noticing that the cluster (C3) identifying the Walker-A, was determined because of the presence, in the alignment, of two large gapped regions starting at the opposite extremes of the alignment and overlapping the motif (Fig 2A). More precisely, the sequences that do not contain the motif, happen to be aligned either on its right hand side or on its left hand side, and all sequences containing the motif, happen to display both the left and the right hand side. No protein sequence, which is homologous to one of the two regions at the right and left of Walker-A, is found combined to the Walker-A only. Sequences aligned either with the right hand side or with the left hand side (from the Walker-A) of the MukB sequence, provide evidence 1. of the evolutionary independence of these two regions in the MukB sequence, 2. of the role of Walker-A as a structural link to combine the two sequences together in three-dimensions, and 3. of the role of Walker-A in the functional evolution of MukB.

The MukB protein shows how the notion of partition helps to understand the complexity of the evolutionary process. It helps to identify contiguous regions having coevolved together in both the sequence and the three-dimensional structure and it does not require clusters in a connected component to form a physically connected network. In this sense, partitions highlight a high order clusters organization. In particular, partitions do not correspond to domains and we suggest them to characterize new evolutionary units for the study of protein structures. The concept is reminiscent of the notion of sector introduced in [15], with the basic difference that it involves the organization of several clusters of coevolved fragments and that it requires a proximity of these clusters on the sequence.

### Fragments Interaction and Structural Stability in Protein A

Does the partition organization provide functional insights in fragment interactions? We demonstrate, on the B domain of Protein A, how partitions can help to identify stable subparts of the protein sequence in the folding process. We do not expect coevolution analysis to give any hint on the kinetics of a folding process but rather on the actors (that is, residues, parts of secondary structures, 3D interactions) of the kinetics process. The level of importance of these actors is encoded within the strength of the coevolution signal and in the three-dimensional interactions identified by partitions.

The B domain of protein A is a three helix bundle protein of 57 residues that has been particularly studied because of its fast kinetics [52]. This alpha protein structure is constituted by three helices and two turns. The first helix (H1) spans residues 10–19, the second helix (H2) 25–37, the third helix (H3) 42–56, the first turn (T1) 20–24 and the second turn (T2) 38–41. Protein A turned out to be particularly amenable to 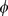-analysis [52] that proposed a number of hotspots, 18 20 23 27 28 31 32 34 35 45 46 49 52, and stated several experimental observations on the structure of the transition states during folding:

1. H2 is the most structured helix;
2. H2 and H3 form a stable or marginally stable intermediate;
3. strong interaction between H1 and H2 during transition state;
4. residues 20, 23 and 27 are implied in a serious destabilization of the protein;
5. H1 is docked in the rate limiting step;
6. no significant structure is found for T1 and T2 in transition state;
7. H3 shows important residues at the N-terminus.

**Figure 4:**
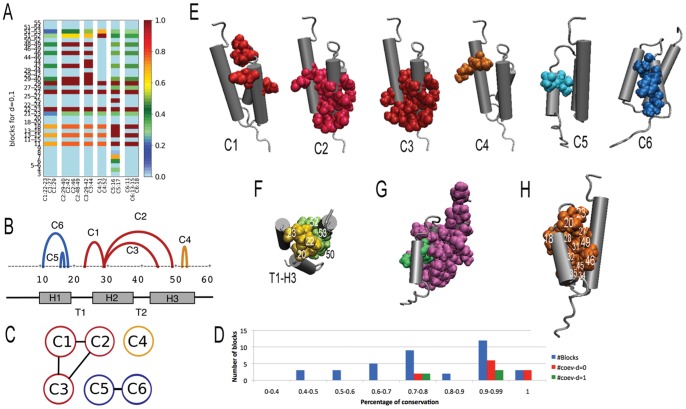
B domain of Protein A. Analysis realized on a dataset of 452 sequences. A: clustered correlated distribution score matrix showing clusters C1–C6 (computed on blocks of dimensions 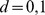; Text S17). B: protein sequence associated to the structure in EFGH; arcs highlight intervals along the sequence that host clusters C1–C6; positioning of helices H1, H2, H3 (grey) and turns T1, T2 is reported. C: interval graph of B; color codes as in B. D: distribution of blocks (blue) with respect to their conservation; all coevolving blocks for 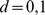 are used. E: clusters C1-C6 are plot on the structure (1bdd); color code as in BC. F: residues in physical contact between helix H3 and turn T1. G: residues 14, 17 (green) and fragment 25–59 (pink) are experimentally validated as important in intermediate folding [52]. H: hotspots from 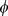-analysis [52].

Co-evolution analysis (run on a dataset of 452 sequences) detected six coevolving clusters: C1 =  (22–23 27 29), C2 =  (29–40 42 46 48–49), C3 = (29–42 44), C4 =  (51 52), C5 =  (16 17) and C6 = (11 13–15 18) (Fig. 4AEFG and Text S4), that are physically connected networks and form three partitions (Fig. 4BC). It sharply agrees with experimental observations, with ten over thirteen 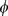-analysis hotspots that are coevolving. Residues 20, 28 and 45 are missing. The partition constituted by networks C1, C2 and C3 (Fig. 4CD) highlights a high number of potential constraints that are coded by the coevolution patterns of these networks and indicates that H2, exhibiting a large overlapping of the clusters, is the most structured helix (observation 1).

The important overlapping of networks C2 and C3 on blocks 29–40 (for C2) and 29–42 (for C3) highlights an evolutionary pressure undergoing a long fragment crossing H2, T2 and H3. It suggests a possible interaction of H2 and H3 in intermediate folding (observation 2) and fits with the experimental evidence that a long peptide fragment corresponding to residues 25–59 defining H2, T2, H3 has much larger helical circular dichroism signals (at 222 nm) than individual fragments of H2 and H3. This fragment turned out to have a much larger signal than the full length model of the denatured state of protein A [52].

All residues at the interaction site of H1 with H2 (14, 17, 18, 32) that have been shown to display high 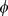-values and to have side-chain interactions in the NMR structure are detected to be coevolving (observation 3). In particular, 14, 17 and 18 coevolve together in C6.

Residues 23 and 27 are detected to coevolve in network C1 and they have been observed [52] to be both playing an important role in the stabilization of the protein (observation 4): mutation on residue 23 generates 90–95% unfolded structures and mutation of residue 27 highly destabilizes the protein. Coevolution analysis highlights that the two positions are not independently important but that there is a strong coevolution pressure going on between them, suggesting that, after mutation, protein destabilization might be a consequence of their interaction.

The two partitions, one constituted by networks C1, C2, C3, and the other by C5, C6 (Fig. 4C), suggest a separation in the folding process into two main independent events, one involving H1, exhibiting a large overlapping with C5 and C6, and the other involving H2 and H3 (and T1, T2) in agreement with observation 5.

From the overlapping of C2 and C3, it seems that any hypothesis on the potential role of T2 in transition state (observation 6) should be analyzed in the context of the structure of H2 and H3. Concerning T1, BIS highlights block 22–23 as important and the coevolution (C1) of this block with residues 27 and 29 of H2 suggests analyzing the potential structure of T1 in transition state (observation 6) by coupling it with the structure of H2.

Consensus between simulation and experiments on the structure of H3 is unsettled [52], [53]. Coevolution analysis highlights residues 42 44 46 48 49 as coevolving in C2 and C3, and suggests that the helix might be partially stable due to an evolutionary pressure involving only the first half of it. The prediction on H3 N-terminus supports observation 7. The second half of the helix interacts with T1, which potentially might help H3 to stabilize; in fact, the H3-T1 interaction site involves residues 22, 23 and 51, 52 which form two coevolving fragments (22–23 coevolves in C1 and 51 52 coevolve together as residues in C4).

To conclude, the analysis of partitions of coevolving clusters suggests a strong evolutionary pressure affecting fragments in H2 as well as H1 and H3 to guarantee the stability of H2 and, possibly, strong interactions between H2 and H1, H3 at the transition state [52]. Our finding might suggest new possible experimental mutations that could contribute to clarify the folding pathway [52], [54].

It is interesting to notice the intimate link between hits and hotspots of 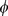-analysis. In fact, 9 over 14 blocks in clusters are defined by hits that are also hotspots of 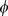-analysis. All clusters contain several hotspots.

The probability to find 25 residues out of 36 experimentally validated ones by choosing 30 alignment positions over 57 (that is, the length of the alignment) is 0.0009. For a small protein with complex interaction patterns like Protein A, this predictive power (accuracy at 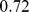, specificity at 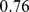, sensitivity at 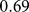 and positive predictive value at 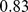) is highly accurate.

### Fragments, Motifs Discovery and Motifs Interaction in the AATPase Family

**Figure 5:**
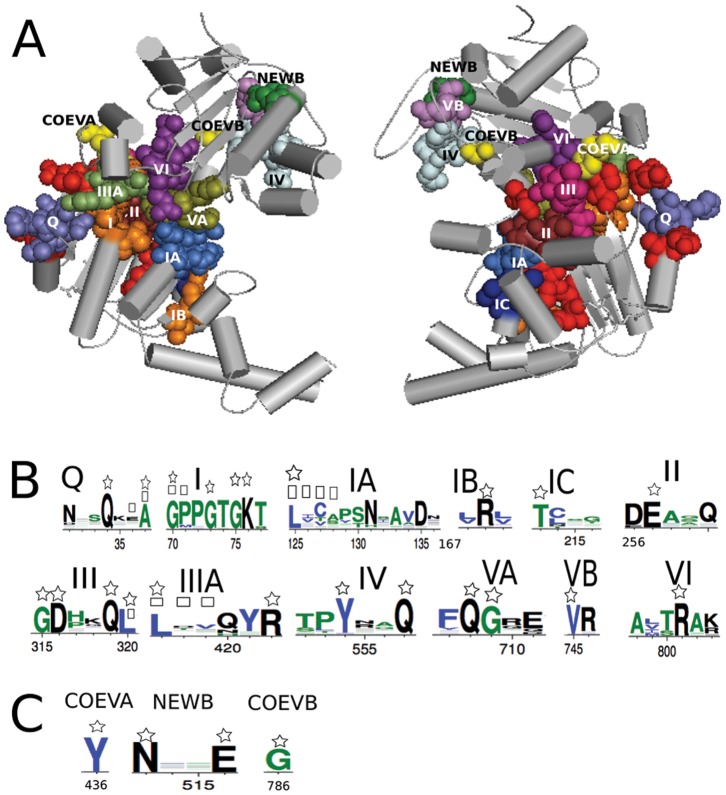
AATPase protein Upf1. A: Upf1 protein structure (2gjk) where all known motifs of the family appear in distinguished colors together with their extensions (in red), one new motif (green, named NEWB) and two more coevolving residues (yellow, named COEVA and COEVB), not know to play a functional role in Upf1, have been identified by BIS. Two domains (left) and their backside (right) are shown (Texts S8 and S15). B: protein logos of all known motifs and their extensions. Residues belonging to the extensions are marked by a square and a star marks those that are coevolving. BIS analysis detects 26 coevolved positions (20 for 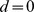 and 6 for 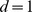) over 677 alignment positions. C: protein logos of the new motif and the two coevolved positions.

Fragments, along a protein sequence, might form functional motifs. Along evolution, these motifs might degenerate with sequence divergence and their identification might become a difficult task. If functionally important sites should remain relevant after evolutionary changes, it might be that coevolution analysis, rather than conservation analysis, could help to localize motifs along the sequence with high precision. In this respect, we show that coevolution analysis can fruitfully revisit already treated protein families to bring new biological insights into play. We carried out a complete coevolution analysis for helicases, that is enzymes that use ATP to bind and unwind a double stranded DNA into its component single-strand [55]. We considered eleven SF1 and SF2 helicase subfamilies that are very diversified in function and highly similar in structure, and for which a classification was proposed based on the C and N-terminal domains surrounding the structural core and on their associated functions [56]. Coevolution analysis of the two core domains highlights the coevolution of specific blocks within the domains and indicates, in this way, regions along the sequence that are susceptible to carry functional signals. Strikingly these blocks agree with the motifs that have been manually identified in [56], [57] and that have been grouped together accordingly to their biochemical function: ATP binding and hydrolysis (motifs are named Q, I, II, IIIa, VI), nucleic acid binding (Ia, Ib, Ic, IV, IVa, V, Vb), coordination between polynucleotide binding and ATPase activity (III, Va). Some of these motifs were originally highlighted in [58] (7 motifs were proposed) and extended in [59]–[62] (4 more motifs were added). See Fig. 5.

The large alignment length (of about 600 amino acids) and the small number of sequences associated to each protein subfamily (varying from 6 up to 67) did not prevent an insightful coevolution analysis. Motifs prediction based on coevolution is realized with a high accuracy (varying between 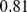 and 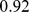) in all 11 protein subfamilies (Table 1). For each SF subfamily, the ratio between the number of coevolving residues belonging to all known motifs [56] and the total number of coevolving residues in the alignment vary between 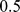 and 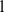 (Text S5 reports ratios and correlations between motifs). This means that a large part of coevolving residues of AATPase families is involved in known motifs but that there are a number of them that might have been missed and it is worth looking at their positions along the sequence to extend known motifs and to localize new motifs. We analyzed all subfamilies in SF1 and SF2. The SF2 families show ratios higher than 0.79 (Text S5) and all their coevolving residues, which do not already belong to a motif, are either located near to known motifs or grouped together along the sequence. This suggests that motifs can be extended and that new motifs can be defined. In the SF1 subfamily Upf1 illustrated in Fig. 5, we could extend motifs I, III, IIIa, Va and VI, and identify a new motif as well as two more residues as potentially playing some functional or structural role. Some of these motifs extensions are obtained on the analysis of other SF1 subfamilies and this supports their interest (Text S6). The full list of extensions and new motifs is reported in Text S6 for SF1 and SF2 subfamilies. Also, the alignment of all subfamilies highlights motifs positions and new motifs appearing in several subfamilies as, for instance, the SF2 subfamilies RecQ and Ski2-like.

**Figure 6:**
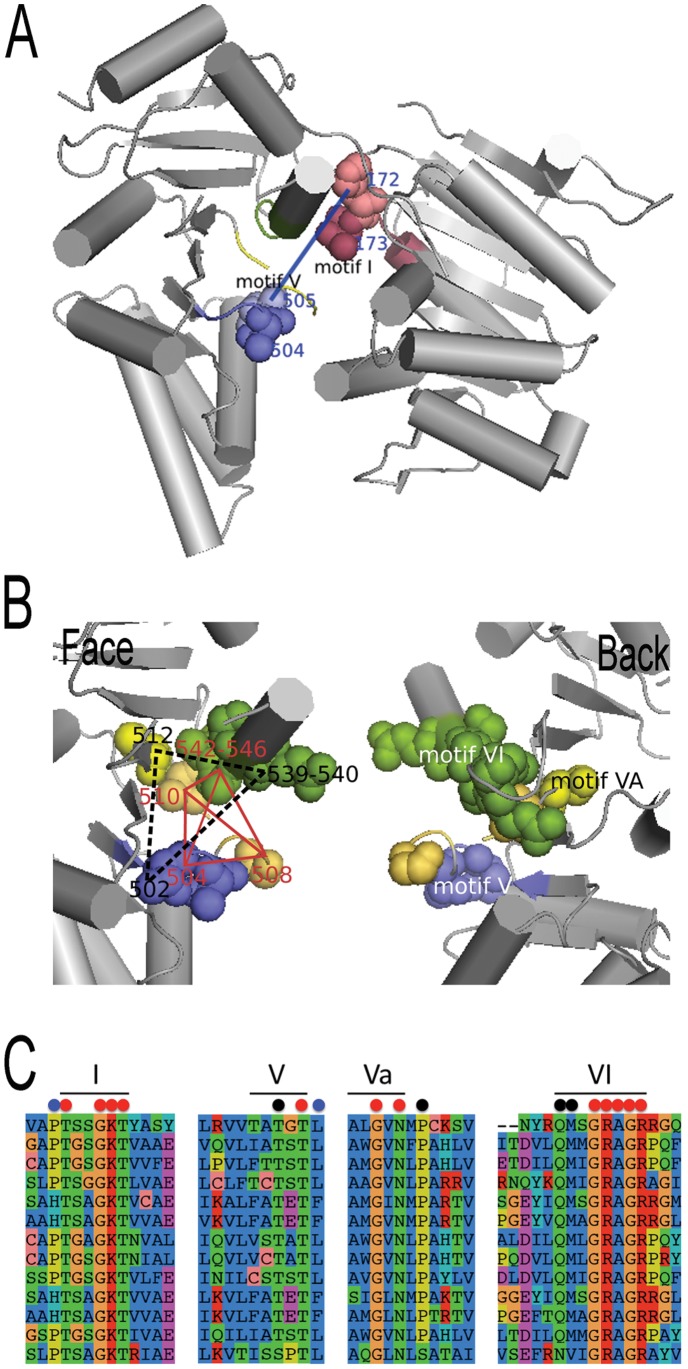
AATPase protein Ski2-like. A: Ski2-like protein structure (2xgj) where four motifs of the family appear in distinguished colors: I (dark red), V (violet), VA (yellow), VI (green). A pair of coevolving residues (172 and 505), belonging to motifs I and V, is highlighted by a blue link. These residues are hits of two coevolving blocks whose atoms are in spherical representation. The blocks belong to different structural domains. B. Coevolution analysis shows that motifs V, VA and VI are related by groups of coevolving residues (left). Residues are drawn in spherical representation and their groupings are highlighted with red and black links. The backside of the domain (right) indicates motifs names. C: four parts of the alignment of Ski2-like sequences describing the four motifs whose location is indicated by a black bar. Colored dots highlight positions with the same coevolution or conservation signal: red dots are completely conserved; black and blue dots display parallel mutations. Note that dots not included in a known motif (like the blue ones in I, V and the black one in Va) describe positions proposed as extensions of the motif. Color maps as in AB.

Across all AATPase subfamilies, we observed that coevolving residues might appear as parts of known motifs suggesting that not all residues in a motif might be functionally relevant. Also, different motifs might overlap several coevolving clusters of blocs suggesting an interaction among motifs. The Ski2-like family, for instance, shows multiple correlations between residues in motifs V, Va and VI (Fig. 6B) induced by three different clusters. One cluster contains completely conserved residues but the other two display simultaneous mutations (Fig. 6C). Spatial proximity supports the hypothesis of their interaction. Correlation among motifs belonging to different protein domains is also observed, for motifs I and V (Fig. 6A). This means that residues belonging to motifs involved in different biochemical functions affecting AATPases might constitute clusters. (A full account of this functional analysis is reported in Text S6 and Text S5.) In conclusion, functional relations between motifs are traceable through either coevolution signals or structural proximity, and for proteins with unknown three-dimensional structure, coevolution analysis becomes the major pathway to access this information.

### Coevolution Analysis Based on Physico-chemical Properties of Residues

Amino-acids changes in sequences are considered more or less important if realized within the same physico-chemical class or not [63]. We tested whether a coevolution analysis based on physico-chemical properties of residues provides sharper information on residue correlation. BIS based on physico-chemical classes, named here BIS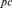, was run on the four protein families described above (Text S7). Overall one notices that coevolving blocks predicted by BIS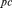 are essentially the same as those highlighted by BIS, but they are detected with a stronger clustering score. Also, the number of coevolving blocks has the tendency to augment for BIS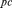 providing predictions that should be experimentally confirmed. In fact, with respect to the pools of residues that have been experimentally confirmed to be relevant, one notices that BIS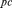 performs slightly more poorly than BIS (Text S7 and Table 2). It is worth noticing that for MukB, BIS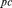 detects the Walker-A motif with the same precision as BIS. For Protein A and Ski2-like, BIS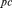 performs slightly better than BIS because of a few experimentally validated residues that have been successfully identified by BIS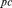. On the AATPase protein families, BIS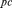 predicts most of the residues and blocks predicted by BIS and some new ones. The new predictions are usually not localized within nor close to known motifs contrary to BIS detections (Text S7), and could not be proposed as possible extensions of known motifs but rather as new potential motifs.

**Table 2:** Comparative analysis with co-evolution and conservation methods.

	API	Pos	#Exp	#CoRes	TP	FN	FP	TN	Prob	Sen*	Spe*	Acc*	PPV*
**Amyloid**													
BIS	0.87	43	25	27	22	3	5	13	7.79*e* ^−5^	**0.88**	0.72	**0.81**	**0.81**
MST	0.87	43	25	16	12	13	4	14	7.88*e* ^−2^	0.48	**0.78**	0.60	0.75
ELSC	0.87	43	25	28	20	5	8	10	1.82*e* ^−2^	0.80	0.56	0.70	0.71
MI	0.87	43	25	25	20	5	5	13	8.15*e* ^−4^	0.80	0.72	0.77	0.80
SCA-DB	0.87	43	25	–	–	–	–	–	–	–	–	–	–
SCA-TM	0.87	43	25	–	–	–	–	–	–	–	–	–	–
Rate4Site	0.87	43	25	26	21	4	5	13	2.75*e* ^−4^	0.84	0.72	0.79	**0.81**
ConSurf	0.87	43	25	17	13	12	4	14	4.77*e* ^−2^	0.52	0.78	0.63	0.76
ET	0.87	43	25	16	13	12	3	15	1.88*e* ^−2^	0.52	**0.83**	0.65	0.81
**MukB: Walker-A**													
BIS	0.84	234	8	8	7	1	1	225	**9.16** ***e*** **^−12^**	**0.88**	**1**	**0.99**	**0.88**
MST	0.84	234	8	–	–	–	–	–	–	–	–	–	–
ELSC	0.84	234	8	13	7	1	6	220	1.93*e* ^−9^	0.88	0.97	0.97	0.54
MI	0.84	234	8	-	-	-	-	-	-	-	-	-	-
SCA-DB	0.84	234	8	8	7	1	1	225	**9.16** ***e*** **^−12^**	**0.88**	**1**	**0.99**	**0.88**
SCA-TM	0.84	234	8	8	7	1	1	225	**9.16** ***e*** **^−12^**	**0.88**	**1**	**0.99**	**0.88**
Rate4Site	0.84	234	8	83	7	1	76	150	3.37*e* ^−3^	0.88	0.66	0.67	0.08
ConSurf	0.84	234	8	106	8	0	98	128	1.53*e* ^−3^	**1**	0.57	0.58	0.08
ET	0.84	234	8	109	8	0	101	125	1.92*e* ^−3^	**1**	0.55	0.57	0.07
**Protein A – B domain**													
BIS	0.82	57	36	30	25	11	5	16	9.89*e* ^−3^	**0.69**	0.76	**0.72**	**0.83**
MST	0.82	57	36	17	7	29	10	11	0.99	0.19	0.52	0.32	0.41
ELSC	0.82	57	36	33	24	12	9	12	6.99*e* ^−4^	0.67	0.57	0.63	0.73
MI	0.82	57	36	14	10	26	4	17	0.34	0.28	**0.81**	0.47	0.71
SCA-DB	0.82	57	36	29	22	14	7	14	3.97*e* ^−2^	0.61	0.67	0.63	0.76
SCA-TM	0.82	57	36	28	21	15	7	14	6.06*e* ^−2^	0.58	0.67	0.61	0.75
Rate4Site	0.82	57	36	0	0	36	0	21	1	0	1	0.63	0
ConSurf	0.82	57	36	26	22	14	4	17	2.13*e* ^−3^	0.61	**0.81**	0.68	**0.85**
ET	0.82	57	36	37	26	10	11	10	0.11	0.72	0.48	0.63	0.70
**AATPase: Upf1**													
BIS	0.58	677	64	26	18	46	8	605	2.93*e* ^−14^	0.28	**0.99**	**0.92**	**0.69**
MST	0.58	677	64	35	21	43	14	599	9.66*e* ^−15^	**0.33**	0.98	0.92	0.60
ELSC	0.58	677	64	22	14	50	8	605	2.00*e* ^−10^	0.22	0.99	0.91	0.64
MI	0.58	677	64	-	-	-	-	-	-	-	-	-	-
SCA-DB	0.58	677	64	8	4	60	4	609	3.82*e* ^−3^	0.06	0.99	0.91	0.50
SCA-TM	0.58	677	64	5	2	62	3	610	7.29*e* ^−3^	0.03	0.99	0.90	0.40
Rate4Site	0.58	677	64	23	13	51	10	603	7.96*e* ^−9^	0.20	0.98	0.91	0.57
ConSurf	0.58	677	64	110	57	7	53	560	**6.39** ***e*** **^−44^**	**0.89**	0.91	0.91	0.52
ET	0.58	677	64	135	54	10	81	532	2.19*e* ^−32^	0.84	0.87	0.87	0.40
**AATPase: Ski2-Like**													
BIS	0.51	686	62	35	22	40	13	611	1.47*e* ^−16^	0.35	0.98	0.92	**0.63**
MST	0.51	686	62	53	32	30	21	603	5.15*e* ^−24^	**0.52**	0.97	**0.93**	0.60
ELSC	0.51	686	62	70	13	49	57	567	6.13*e* ^−3^	0.21	0.91	0.85	0.19
MI	0.51	686	62	-	-	-	-	-	-	-	-	-	-
SCA-DB	0.51	686	62	6	3	59	3	621	1.16*e* ^−2^	0.05	**0.99**	0.91	0.50
SCA-TM	0.51	686	62	6	3	59	3	621	1.16*e* ^−2^	0.05	**0.99**	0.91	0.50
Rate4Site	0.51	686	62	36	20	42	16	608	1.49*e* ^−13^	0.32	0.97	0.92	0.56
ConSurf	0.51	686	62	134	62	0	72	552	7.85*e* ^−51^	**1**	0.88	0.90	0.46
ET	0.51	686	62	196	62	0	134	490	6.15*e* ^−38^	**1**	0.79	0.80	0.32

Columns are as in Table 1. Coevolution (white) and conservation (grey) analysis are realized on sets of 80 Amyloid sequences, 200 for MukB, 452 for Protein A, 18 for Upf1 and 13 for Ski2-like. All conservation analysis systems, ConSurf [49] and Rate4Site [65] are run on our sets of sequences, while ET uses its own dataset. Best performance is highlighted in bold; when neither a cluster nor a matrix could be constructed, the symbol - is used. For Amyloid, experimentally validated residues (#Exp) have been obtained from the sources cited in the text; for Protein A we considered hotspots together with the large peptide fragment 25–59 and residues 14, 17 cited in [52] to have high 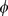-value; MukB analysis is evaluated on Walker-A detection; Upf1 and Ski2-like are evaluated on known motifs described in [56]. The number of (coevolving or conserved) residues detected by the methods are reported in column #CoRes. See SI Tables 3–4, 11, 19, 28, 36, 41–49. Sen, Spe, Acc, PPV have been evaluated with respect to experimentally validated residues; they are marked with * to remind this. The same is true for Prob.

### Comparison with Other Methods of Coevolution and Conservation Analysis

The evaluation of predictive methods of coevolution analysis is inherently difficult due to the very limited understanding of how proteins fold, function, behave mechanically. Nonetheless, by taking into consideration all residues that are experimentally known to have some functional or structural importance, we might be able to check, at some extent, the behavior of the system. Instead of measuring the precise accuracy, specificity, sensibility and positive predicted value of the predictions, which would demand the knowledge of the exact pool of residues that are structurally or functionally important for a protein, we attempt to measure the capability of a method to predict those signals that appear to be the easiest to detect by today's experimental approaches. There are intrinsic limits to this evaluation: on the one hand, the set of validated residues might miss some important ones, and on the other hand, a method might predict residues that have never been experimentally highlighted before. Therefore, the evaluation will possibly underestimate the performance of the predictive system. Aware of these limits, we realize a test against the set of proteins analyzed above, a rather well known set supported by several experimental studies.

We considered five systems of coevolution analysis and three of conservation analysis, and evaluated them on our 14 protein families (Texts S2, S4, S5, S8 and S9; see Methods) generating, in this manner, 112 experiments to be used for comparison. We considered two main comparative criteria, the convergence of a system and its performance. BIS answers on all 14 protein families while Statistical Coupling Analysis (SCA-DB and SCA-TM) [12], [64], Maximal SubTree Method (MST) [19] and Mutual Information (MI) [8], [64] do not. Over the 112 experiments (Table 2 and Text S10), only 9 showed other systems to display a better behavior than BIS and 4 experiments demonstrated a comparable behavior between BIS and other methods (Table S1). The 9 experiments concern 5 different systems (SCA-DB, SCA-TM, MST, ConSurf [49] and Rate4Site [65]) and 5 different AATPase families, and the 4 experiments highlight a different protein family for each system.

Compared to systems of coevolution analysis, BIS systematically obtains high specificity and accuracy, and maximum positive predictive values (Table 2). Statistical methods demonstrate difficulties in dealing with highly conserved sets of sequences and datasets made of few sequences while combinatorial methods appear suitable for this purpose. Among all statistical methods, ELSC [66] is the closest to BIS. It behaves a little less well than BIS on all experiments, but the differences between the two systems on each performance measure are almost always small (that is 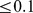).

Even though designed to primarily consider co-evolution signals, BIS has been evaluated against systems of conservation analysis (Table 2, Text S10 and Text S11). With respect to these systems, it outperforms on families of very conserved sequences, that is, on Amyloid, Protein A and MukB (Table S1). In particular, it detects the Walker-A motif in the MukB protein as a separate coevolving cluster. The prediction of this motif has been an important challenge for conservation analysis systems before and BIS shows being highly specific and accurate on it. On AATPase subfamilies, BIS is competitive, displaying much lower sensibility (it detects fewer true positives) than these systems in many experiments but a higher positive predictive value. It should be noticed that coevolved pairs of fragments, as opposed to conserved residues, bring to light relations between pairs of blocks within a sequence, and in the case of the AATPase families they highlight relations among specific motifs that cannot be detected by conservation analysis.

It is important to observe that blocks that match experimentally observed fragments have been ignored and this suggests an even better performance of our method.

Finally, it is worth mentioning here that the comparative analysis of the 14 protein families, highlighted a few false positives that are shared by several methods (Texts S2, S4, S8 and S9). These residues are not known to play an important biological role but appear as good candidates to be experimentally tested.

## Discussion

Coevolution analysis highlights the combined functional or structural role of groups of residues, possibly organized in fragments, in a protein and can suggest complex combinations (pairs, triplets or tuples) of amino acid mutations to be tested by experiments studying protein structural stability or functional activity. The whole space of combinations cannot be approached experimentally in a straightforward manner: an exhaustive testing would be impossible because of the large number of combinations, experimental difficulties and costs, and a random selection would be useless because of the extremely small chance to be successful. The existence of a predictive method that can suggest a small subset of combinations to try is of great help to biologists and structuralists. Based on BIS, hits in a cluster or hits shared by several blocks could define predicted pools of residues to try in mutagenesis experiments. Similarly, blocks either containing specific hits (for instance, hits with multiple occurrence) or appearing in several clusters could be the first ones to study in circular dichroism experiments in order to identify their individual role in folding.

It should be noticed that BIS does not analyze blocks of any given length, but it selects a pool of them for coevolution analysis. Such a selection, within the huge combinatorial space of fragments, is based on the conservation of amino acids within the block.

### A Mathematical Framework to Define Coevolution

The definition of a precise mathematical framework to analyze coevolution signals allowed us to demonstrate that fragments, and not just isolated residues, are the coevolving units for pools of conserved sequences. *In vitro* experiments revealed that these fragments play important functional and structural roles for protein families. For divergent pools of sequences, these fragments degenerate into single residues. This hints that accurate sequence evolution models might be based on fragment evolution and not on residue evolution. Also, the mathematical framework allowed us to define and automatize new formal notions describing the structure of the coevolution signal. Today, the biologist can investigate a fine structure of the signal, this being associated to single residues, blocks or clusters of blocks. Long and short distance correlations between residues, between fragments or between clusters can be evaluated.

### Analysis of Fragments Versus Analysis of Residues

Our approach to coevolution analysis considers blocks of positions corresponding to protein fragments. The basic observation is that, very often, fragments of consecutive residues display very similar coevolution patterns and because of this, one can directly address correlations between blocks instead of single positions in an alignment and study correlations between distant fragments in a sequence. As a consequence, one observes:

1. large protein fragments, known to display functional or structural roles, which are detected as blocks. Such fragments are formed by hits and by positions (typically conserved positions) that do not disrupt the word distribution of the hits. An example is the long fragment 29–42 of Protein A detected as a unique coevolving block by extending hit 41 with 29–40 and 42. Left and right extensions are made of conserved residues preserving word distribution at position 41. The fragment was shown by 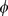-analysis to be part of an even longer fragment playing a role in protein stability [52]. Other important examples are the fragments highlighted for the Amyloid beta peptide, among which fragment 25–35 that is known to best bind 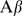.
2. residues that belong to different clusters. These residues might play multiple roles in the functional and structural constraints of the protein. This is the case for residue 29 in Protein A which belongs to clusters C1 and C2, where in C1 is coevolving with residues 23 and 27 both playing an important role on protein stabilization [52] and in C2 is coevolving within a large peptide fragment potentially involved in the formation of a (marginally) stable H2-H3-intermediate [52].
3. a reduction of the computational time in coevolution analysis with a very important effect in the treatment of very long protein sequences.

Note that 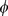-analysis strongly highlights the importance of studying protein folding through protein fragments, the need of identifying fragments and of measuring their stability. Similarly, we expect to detect structural and functional insights through the analysis of evolutionary signals coded within blocks of residues.

### Some Remarks on the Definition of Blocks

A naive post-processing of coevolving residues simply combining adjacent residues together does not allow for the identification of blocks. This is due to the fact that a block can be composed by alignment positions having different amino acid distributions and that not all residues in blocks coevolve together. In the Amyloid beta peptide, for instance, BIS block analysis detects fragment 18–20, known to correspond to motif VFF playing a crucial role in the diagnosis of amnestic impairement, while BIS run on positions identifies residues 18 and 20 missing residue 19. Fragment 18–20 could be reconstructed from the two isolated residues 18 and 20 by a trivial pre-processing that puts together close coevolving residues, but this suggestion, even if providing a suitable prediction of the fragment 18–20, will not work correctly on other circumstances. In fact, for the Amyloid fragment 16–20, known to be the region that most efficiently binds to 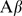, BIS analysis based on positions detects cluster 11 14 16 while BIS block analysis detects 11–12 14 16–20. A post-processing following the strategy of filling up proximal coevolving residues with adjacent residues could wrongly suggest the formation of the block 11–16 that is not known to be biologically meaningful. Protein A provides another example highlighting the importance of a careful definition of a block: the overlapping of some coevolving blocks in Protein A, involving helices H2 and H3, suggests a strong interacting role of the two helices during intermediate folding. A naive post-processing that would not produce blocks allowed to overlap would not help to identify the interactive role of protein fragments. These examples illustrate well potential ambiguities and simplifications introduced by a post-processing step that does not explicitly use our definition of a block (where a few residues are highlighted as hits and the ones around them, preserving the distribution of words, are associated to them to play a joint role). In particular, they highlight that coevolution analysis based on positions cannot replace block analysis.

Other finer approaches could be suggested to the formation of blocks based on coevolving positions. For instance, one could first compute mutual information or conditional entropy as the similarity between every two columns of the alignment, and then group columns together in descending order of similarity values. There are two main drawbacks to this proposition: 1. it demands the introduction of a threshold for blocks identification and the evaluation of this threshold, 2. it produces blocks that are not overlapping. It is worth noticing that coevolving blocks can be defined from coevolving alignment positions. This can be done by considering these latter as hits and by applying BIS definition of block to properly identify hit extensions. This way to generate blocks guarantees the identification of overlapping blocks that we showed to be important in protein analysis.

One could check whether other types of blocks in sequences, used in other studies on proteins, could be of interest for coevolution analysis. “Hydrophobic” blocks, that is blocks mainly composed of hydrophobic amino-acids, have been used for several purposes in the past as for guiding the folding process [67]–[70], the detection of secondary structures [71], the alignments of very diverged protein families [71], [72], the detection of new homologous sequences [73]. The mathematical framework developed here could be used to analyze coevolution of such blocks, but the biological relevance of the results should be carefully evaluated.

Blocks definition is sensitive to the protein sequence alignment. When wrong sequences are included by mistake in a sequence alignment, they might generate undesired effects in block identification. To avoid considering words included by ‘wrong’ sequences (in the distribution of words) in a block, we introduced the notion of exception that allows excluding such sequences from the analysis. The exclusion of these sequences does not affect signal detection and we could successfully extract precise coevolution signals even for families represented by less than a dozen sequences, like the AATPase families.

For each block, the definition of an exception asks for a word to be supported by at least 2 sequences in the alignment. The number 2 is a minimal condition required to detect the persistency of a signal and the smallest non trivial lower bound that one can ask for: if a word in a block presents more than 2 occurrences, it will be considered in the analysis. Ultimately, the evolutionary interest of the block will be evaluated by BIS coevolution analysis.

By varying the set of aligned sequences representing a protein family, one might ask whether the blocks identified for the family vary as well. It is simple to see that a set of aligned sequences 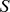 increased by the addition of one more sequence, say 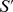, allows for the identification of the same blocks, but that a block computed for 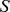 and 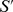 might be detected for different numbers of exceptions. To see this, one should observe that the definition of a block is dependent on a fixed number of exceptions and that exceptions count the number of sequences that support single words (words with no repetition) within the block. This means that if the new sequence in 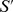 contains a new word for the block, it will introduce one more exception for the block. Hence, if the block was identified in 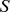 with a number of exceptions 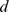, it will be identified in 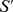 for 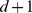 exceptions. In general, if 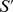 is a set of aligned sequences containing a smallest set 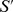, then a block detected in 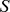 with 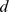 exceptions will be detected in 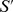 with 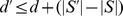 (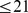) exceptions. Notice that BIS computes correlations between blocks identified with a number of exceptions 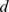, but also between blocks identified with a number of exceptions 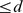, allowing for correlation analysis between blocks identified with different exceptions.

### Coevolution Analysis of Families Represented by Few Protein Sequences

Ideally, coevolution analysis is better realized over a large number of sequences but certain families (or subfamilies) are described by just a few sequences, and BIS tries to extract the existing signals of coevolution from them. The request that a word in a block should be repeated at least twice ensures that, in a block, all distinguished words bring a meaningful proportion of the signal. This proportion becomes less and less important with an increase in the number of sequences (remember that BIS asks just two sequences to support a word). But for a few sequences, it is relevant that such a condition be respected. For a set of 6 sequences, for instance, two occurrences of a word correspond to a contribution weight that is roughly a third of the signal. Depending on the number of sequences, one might want to estimate whether to trust or not the analysis. One way to do this, would be to estimate precisely the probability of obtaining a fixed distribution of 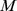 distinguished words over 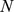 ordered sequences knowing the number of occurrences for each word. This is given by the formula 
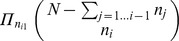
 where the values 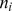 represent the number of occurrences of the word 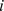. We consider only words with more than 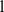 occurrence and fix the values 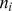 by decreasing order. It is easy to see that for a small set of 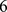 sequences, a block formed by 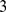 distinguished words of 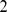 occurrences has a chance 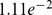 to be randomly generated. If the set is made of 7 sequences, the distribution of 3 distinguished words of 2 occurrences has probability 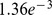. Based on this probability, one could decide to consider as statistically interesting only those blocks that can be generated randomly with a probability smaller than a certain threshold, say 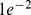. The user should evaluate the parameters in his/her data analysis.

For our 14 families, we checked the effect of family size on block size (Text S12), and observed that for small families (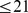 sequences, where 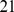 counts the number of amino-acids and a gap; this threshold represents the case of maximal variability for a position in a small set of sequences), BIS identifies many blocks of small sizes and no large block (maximal size for a block is 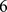 residues and average size is 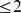 residues). The detection of small blocks rather than large ones is due to our requirement that blocks be defined on exceptions. In fact, if the condition on exceptions is dropped and all positions of an alignment are considered as hits, block size can strongly increase (Text S12). In particular, the chance of having positions with full variability is non negligeable for small families, and for such positions the associated blocks would have the size of the alignment length. Such blocks are obviously uninteresting. On the contrary, based on exceptions, we demonstrated BIS small blocks to overlap well known motifs on the AATPase subfamilies (BIS being highly specific; see Table 2 and Text S10).

One should be warned that large blocks are not necessarily uninteresting. In fact, BIS detected block 26–42 as coevolving with two other residues 21 and 22, all known to play an important role for Amyloid (see above). On the other hand, the B domain of protein A shows to contain a block of length 16 (with hit in alignment position 43 and terminating at position 44) that finally does not co-evolve with other positions. Families represented by conserved sequences might display a number of these blocks. BIS coevolution analysis shows to filter them successfully.

### Clusters Interaction

Coevolution analysis outputs one or several clusters of correlated blocks. It identifies them automatically through an unsupervised non-hierarchical clustering algorithm. This means that a structure among coevolved clusters, if any, might be identified. In fact, clustering predicts groups of residues possibly sharing some amino acids, and the multiple role of a given amino acid in several clusters can provide some explanation of its interaction with different residues during (intermediate steps in) folding or functional activation. BIS proposes two ways to determine clusters relationships, either through shared residues or through the overlapping between intervals hosting clusters along the sequence (that is, partitions). BIS is the first bioinformatics attempt to explore clusters interactions and it provides the first evidence that such an analysis can actually be done. An experimental and theoretical development going towards the same direction was done first by 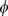-analysis [52].

### Large Scale Coevolution Analysis and Dataset Dependencies

Our comparison between BIS and existing systems of coevolution analysis (Table 2) highlights that the problem of a precise identification of coevolution signals is neither obvious nor solved. Predictions proposed by different methods highly depend on datasets properties, as the number of sequences and their rate of divergence. A summary of methods performances on different datasets is reported in Text S10. When aiming at the development of a large-scale system predicting coevolution signals for arbitrary protein families, a precise analysis of these properties should be realized to eventually run different coevolution approaches on different protein families. Properties such as sequence conservation or presence/absence of very divergent sequences determine the possibility for a method to propose a prediction. Statistical methods demonstrated systematic difficulties in dealing with highly conserved sets of sequences and with datasets made of few sequences while combinatorial methods appear suitable for this purpose (see Table 2). We tested whether the removal of highly similar (possibly identical) sequences from the datasets is crucial or not for coevolution detection and found that variability needs to be guaranteed for statistical methods, and that combinatorial methods succeed to analyze both kinds of sets. On the other hand, both types of methods seem to be highly affected by the presence of few divergent sequences in conserved datasets.

BIS amenability to treat especially conserved sequences makes it a favorable platform to search for coevolution signals between protein binding sites of protein partners, these regions being particularly conserved within a structure. Also, because of its potential to treat sub-represented protein families, BIS is an ideal approach to analyze specific signals of coevolution appearing in protein subfamilies where very few sequences are available. At the moment, we do not know of any method that can fill these expectations and BIS becomes a good starting point for the development of novel approaches to protein interaction and protein subfamilies exploration.

### Differentiation of the Functional and Structural Origins of Coevolution Signals

A basic question to which coevolution analysis should answer is about the functional and structural origin of the coevolution signal. Functional interactions, conformational changes and folding dynamics are reasons used to justify the presence of coevolving fragments, and the problem of automatically distinguish these complex evolutionary pressures is wide open today. For instance, as already argued in [12], [13], we observe that coevolving networks are often made of residues that are localized in close three-dimensional proximities. This is not always the case though since we can observe complex interactions inducing coevolution of different structural spots that become physically connected only through interacting ligands or conformational changes. A better understanding of the structural, that is physico-chemical and geometrical, patterns of coevolving regions might help to characterize and discriminate signals of structural origin. Another example is given by coevolving fragments that are identified for different protein subfamilies. Subfamilies might be characterized by functional diversities, as it is the case for the AATPase family or for sets of sequences that are phylogenetically proximal and within which one might want to investigate functional evolution. Often, coevolution analysis of subfamilies asks for analyzing a small number of protein sequences and possibly families of sequences that are rather conserved. It shows that coevolution analysis can be successfully done under these stringent hypotheses and that the structure between signals provides an important advancement towards this difficult and challenging goal.

*Towards a human map of coevolved residues.* Mutation of coevolving residues can have an important effect in structural destabilization or in weakening the functional activity of the protein with implications that are noticeable at the clinical level and that are identified as genetic diseases. The Amyloid Precursor Protein provides an example of this fact. The thorough study of coevolution signals on the human proteome scale will furnish a genetic map that can be of great interest for the geneticists investigating mutations that could be the cause of observed phenotypes or clinical pathologies. Using approaches like BIS, we expect to be able to identify an ensemble of deleterious mutations that are susceptible to be causing diseases. Typically, just a subset of these mutations is sufficient to imply deleterious effects and, today, we have no way yet to sharply identify and characterize such subsets.

## Methods

To describe the sequence analysis, we consider a sequence 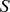 with a known three-dimensional structure and 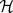, a set of 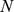 sequences that are homologous to 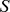. We consider an alignment of the sequences in 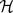, where 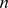 is its length.

### Exceptions for Consecutive Positions in an Alignment

Let us consider a group of 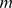 consecutive positions in the alignment, where 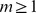. For each sequence in the alignment, there is a uniquely identified subword in it that belongs to the group of positions. There are 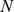 such subwords and we ask each of them to have at least two occurrences, with the *exception* of 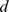 subwords, for 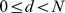. The value 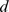 is uniquely associated to the group of subwords.

### Hits

Each position of an alignment, a *hit*, is uniquely associated to a number 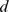 representing the number of residues with no repetitions along the alignment column. Notice that 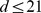, that is its value is bound by the number of amino acids (where a gap is considered as the 21st residue). Intuitively, the presence of several hits displaying no exceptions (that is 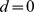), suggests the set of sequences in the alignment to be well represented. In fact, one expects the alignment of sequences that are homologous to 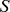 to have almost all hits at 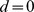. At the contrary, the alignment of sequences that are not homologous to 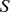 are expected to have almost all hits at 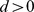. In particular, the presence of several hits with 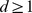 exceptions is an indicator either of sequences that are non-homologous to 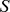 but erroneously occurring in 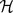 or of sequences in 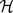 that are distantly related.

### Extension of a Hit Preserving the Number of Exceptions

**Figure 7:**
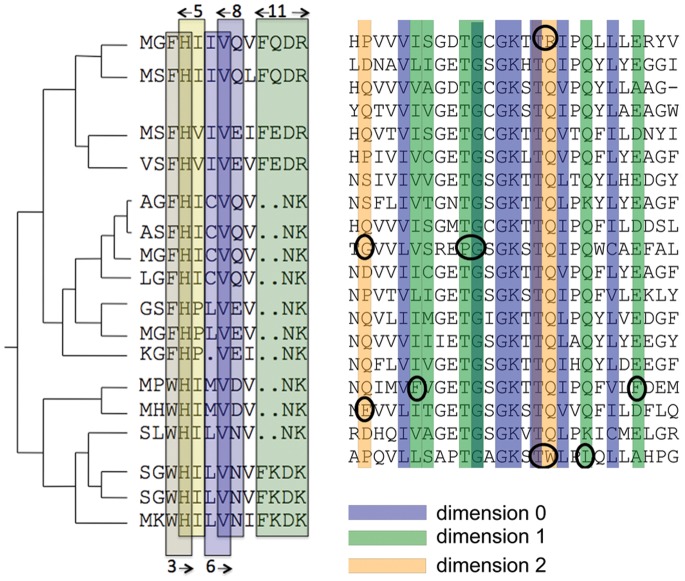
Hits, pseudo-blocks and blocks. Left: Aligned sequences and associated distance tree. Among 13 aligned positions, 3 4 5 7 8 10 11 12 13 are hits of dimension 0, 6 9 are hits of dimension 1, and 1 2 are hits of dimension 4. Position 6 is a hit of dimension 1 because of a gap, which is considered as a distinguished amino acid. Four hits can be extended to blocks of size 2: hit 3 extends to 3–4, 5 to 4–5, 8 to 7–8 in dimension 0, and 6 to 6–7 in dimension 1. Extensions are not symmetric (e.g. hit 7 does not extend to block 6–7). Hit 11 extends on the left and on the right to pseudo-block 10–13 of size 4; the associated block is 12–13 due to more than 60% of gaps in positions 10 11. Hits 1 2 4 7 9 10 11 are considered blocks of size 1. Right: For some of the blocks, the exceptions, identifying their dimension, are circled. Exceptions might be single residues or words. All hits of dimension 0, 1 or 2 are highlighted in color.

Given a hit 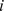 with 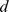 exceptions, we extend it into a group of two columns (by considering either the 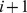 column or the 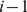 column) of at most 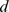 exceptions whenever: 1. the number of exceptions of the adjacent column is 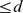, 2. the exceptions of the adjacent column (if any) occur in the same sequences collecting the exceptions of column 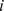, and 3. if two sequences have distinguished words in columns 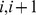 (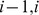), they must have different words in column 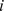. The three conditions ensure that the number of exceptions does not increase after a valid extension. Following the definition, whenever a column 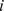 can be extended both on the right and on the left, we say that the group made of the three columns 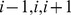 has 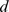 exceptions. The conditions to extend a hit 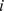 can be restated as conditions for extending a *group of consecutive positions* instead, since a hit can be considered as a “group” made of one position. The idea being that, given a column with 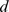 exceptions, we can extend it (step by step by adding a position at a time) to a group of consecutive positions from the left and the right until no larger extensions are possible. We refer to the maximal extension of a hit as a *pseudo-block*. Notice that different hits can generate overlapping pseudo-blocks. See Fig. 7.

### Handling of Gaps and Definition of a Block

Given a pseudo-block, we define a block out of it, possibly several, depending on the gaps that occur in the aligned words of the pseudo-block. Let us consider a pseudo-block with 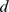 exceptions, defined from alignment position 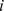 up to position 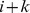 included, and consider the 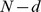 aligned words that, by definition, occur at least twice in the pseudo-block alignment. Based on this subset of aligned words we identify alignment columns (within the interval 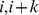) that contain less than 40% of gapped entries. Intuitively, we want to exclude from a pseudo-block, all positions that are significantly gapped. A *block* is the maximal interval corresponding to consecutive positions that: 1. satisfy the gap condition above, 2. preserve the number of exceptions 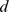 of the hit, 3. localize the exceptions in the hit within the same sequences, and 4. preserve the occurrences of distinguished words within the same sequences as in the hit. The last two conditions assure that the word distribution on a block is the same as the one of its original pseudo-block. For instance, consider the pseudo-block formed by several occurrences of the aligned words 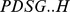 and 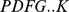 by an extension of hit at position 7. Then we identify the first four consecutive positions as a first block and the seventh position as forming a second block. If the pseudo-block is formed by several occurrences of the words 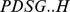 and 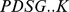, then we shall only keep the block formed by position 7 because the word distribution within the first four consecutive positions changes compared to the hit. In conclusion, a pseudo-block can generate one or more blocks with a given number of exceptions.

The length of a block might vary from 1 up to 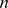, that is the length of the alignment. The length of a block depends on the percentage of identity of the alignment and on the number of exceptions 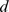. Namely, if the sequence identity of the alignment is high, the probability of having long blocks is also high. If the number of exceptions is high, long blocks can be present even though sequence identity is low. By increasing the number of exceptions, blocks get larger or collapse with other blocks and, in general, at some given number of exceptions, one expects a collapse of all blocks.

A block of consecutive positions 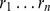 is denoted 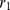
–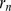, and a cluster grouping blocks 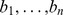 is denoted 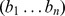.

### Handling of Gaps on Hits

Coevolution can be computed directly on hits for a given number of exceptions 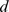 (an option exists in BIS). For this, we select those hits of 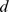 exceptions by filtering out those that are significantly gapped, that is those containing at least 60% of gaps, and compute coevolution only on the remaining ones. In this case, we say that coevolution analysis is realized on *alignment positions*.

### Pairing of Blocks/positions and Pairing Score

**Figure 8:**
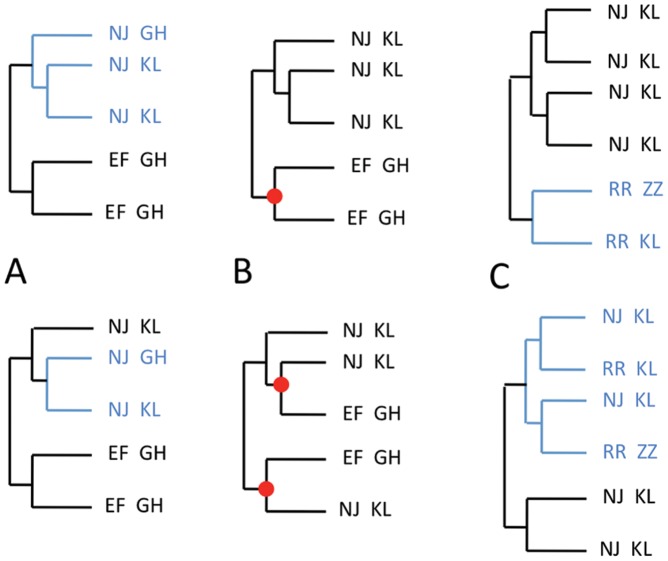
Pairs of blocks within trees. Distance trees describe distinct pairs of blocks 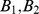, thought to belong to two fixed positions in an alignment. Minimal subtrees where correlation between blocks is not perfect, that is 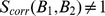, are highlighted in blue. In ABC, trees have the same topology and are characterized by a different labelling of the leaves with the same pairs of words. We call 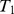 the trees on the top and 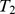 the trees on the bottom. A: the correlation score of 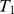 is greater than the correlation score of 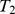 and this because for 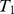 perfect correlation is disrupted on a larger subtree. B: both trees display scores of comparison and correlation 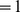. Notice that the method does not distinguish the existence of a single mutational event transforming NJ in EF and KL in GH (top) versus a double mutational event realizing the same transformation (bottom). C: the size of subtrees displaying no perfect correlation has an impact in the correlation score of the full tree. 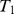 displays a greater correlation score than 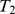, while scores of comparison are the same.

Let us consider two blocks or two alignment positions, say 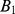 and 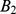, one spanning from position 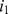 to 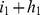 and the other from 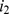 to 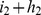 (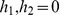 when single positions are considered). The two blocks/positions might have different number of exceptions. Suppose that 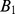 contains the set of distinguished words 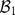 and that 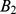 contains the set of distinguished words 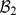. Then we define the *score of comparison* of 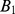 with respect to 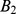 as
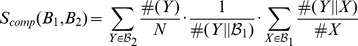
where 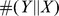 is the number of sequences where the word 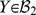 co-exists with the word 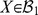, 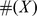 (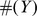) is the number of sequences where 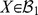 (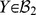), 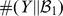 is the number of different words in 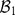 which co-exist with the word 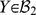, and 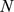 is the number of sequences.


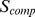
 measures the regularity in the distribution of words in 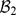 with respect to words in 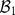. Ideally, in a perfect correlation signal, a word 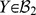 pairs only one word 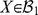, and 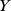 is the only word in 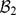 that pairs with 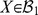. If this is not the case, the formula 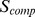 measures the amount of pairs associated to 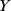. To illustrate the formula, let us consider the tree 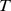 in Fig. 8C top, where we name 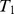 the black subtree of four leaves and 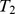 the blue subtree of two leaves. It is instructive to look at the computation of the comparison scores for the three trees, 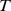, 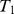 and 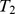. For 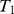, the word 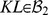 matches 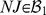 and only 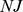. This amounts to a score of comparison 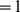. For 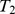, the word 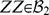 pairs with 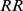 but it is not the only word in 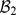 that pairs with 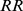, in fact 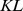 also pairs with 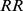. The comparison score takes value 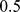 in this case. For 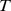, we can appreciate more explicitly the power of the formula. In fact, for each 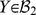, the formula computes the proportion of sequences that contain an 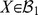 and 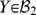 (factor 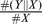), and weights the sum of these quantities in such a way that more distinguished sequences in 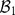 pair with 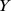, less the contribution of 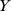 to the coevolution signal counts (factor 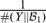). All words 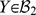 make a contribution to the final score of comparison, but this contribution is weighted by their frequency in the 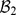 distribution (factor 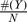). The score of comparison for 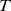 is 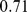.

The set of sequences can be thought to be organized on a distance tree 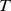. In this case we refer to the score of comparison with the symbol 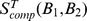. Given 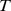, we assign a *score of correlated distribution* of 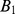 with respect to 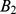 for the sequences of 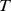 by recursion on the structure of 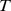 as follows. If 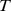 is a leaf, 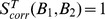. If 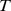 has immediate subtrees 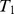 and 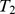, 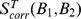 is defined as
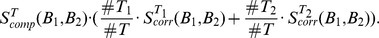



See the examples in Fig. 8. Fig. 8B shows trees displaying a perfect correlation between blocks, that is that distinguished words in a block correspond to distinguished words in the other block and vice-versa: 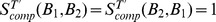 and 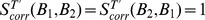, for 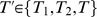. Figs. 8AC display examples of pairs of trees with the same scores of comparison but where correlation scores differ. For instance, in Fig. 8A, correlation scores are 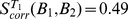 and 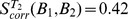 for the top (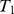) and bottom (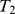) trees. Notice that minimal subtrees 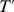 where  (colored blue in Fig. 8) are such that 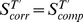. Correlation scores might be non symmetric while their associated comparison scores remain symmetric, as shown with trees in Fig. 8A: 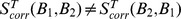, for 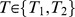. Fig. 8C shows trees whose comparison scores as well as correlation scores are non symmetric. Several general properties of 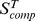 and 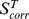 can be deduced. For instance, it can easily be shown that 1. if 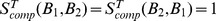 then 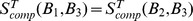 for all 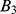, and 2. if 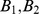 are blocks with 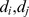 exceptions respectively, where 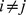, then 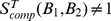 or 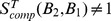.

### Clusters Identified with Respect to (at most) 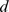 Exceptions

Given the matrix of scores of correlated distribution, we define clusters of coevolving blocks with 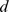 (at most 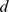, denoted 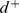,) exceptions by applying the automatic clustering algorithm CLAG [74] with default parameters (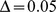 and maximal environmental and symmetric scores; predictions remain unchanged when 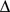 increases to 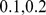) and no aggregation step. To these clusters, for each block with 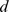 (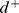) exceptions that does not appear in some CLAG's cluster already and that contains several hits, we add a new cluster defined by the hits of the block. Notice that all hits in a block display the same behavior with respect to other blocks, by definition. For a given 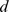, the set of blocks with 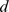 exceptions (also called 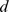
- *environment*) is contained in the set of blocks with at most 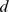 exceptions (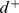
- *environment*). Clusters identified for 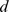 and 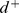 are not identical and they might be specific to the number of exceptions. This is due to two reasons: either to the combination of blocks/positions of mixed number of exceptions (helping to identify new clusters for 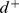), or to the reduction of the number of blocks/positions at 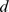 (helping to identify new clusters for 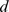). Both conditions are illustrated in Text S3.

### Algorithm

Given an alignment of protein sequences and an integer 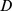 representing the maximal number of errors admitted for capturing the coevolution signals, the BIS method iteratively computes coevolution of either blocks or positions for all numbers of exceptions 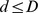. The successive steps of the algorithm are as follows: 1. it either defines blocks from hits or it filters gapped hits out to identify alignment positions. 2. it computes comparison scores and correlation distribution scores for each pair of blocks/positions at 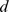 (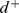). 3. it constructs a matrix of correlated distribution scores. 4. it analyzes the matrix issued in 3 and clusters it with CLAG [74]. 5. it identifies clusters and networks of coevolving blocks/hits. The algorithm outputs several files along the different steps among which the clustered matrix of correlated distribution scores, the list of networks and their numerical properties, the annotated PDB files for network visualization (Text S13). The code implementing BIS algorithm and sample datasets are available at http://www.ihes.fr/ 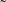
carbone/data10 for both Linux and Mac OSX. Instructions on how to run it and install it are provided in Text S13.

### Percentage of Identity of an Alignment

Given 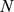 aligned sequences, the percentage of identity at a position 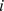 (block 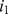
–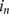) of the alignment, denoted 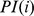 (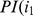
–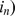), is the number of identical pairs of residues (words) at position 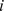 (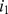
–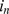) divided by the total number of pairs, that is 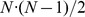. The percentage of identity for the alignment, denoted 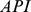, is the mean of the percentage of identity of all alignment positions. See Table 2 and Fig. S1.

### Sequence Alignments

Aligned sequences for all protein families (Table 1) were retrieved from PFAM database v23 (very divergent sequences were manually eliminated; see Text S14) and from [56]. The helicases family is made of the SF1 and SF2 core families [56]. We analyzed all subfamilies with the exception of four SF2 subfamilies that were made of only 2 or 3 sequences. The core alignment considered in our analysis was taken from [56], it puts together SF1 and SF2 families and its length is 864aa. For each subfamily, we extracted the alignment of the corresponding set of sequences. All alignments are available for download at http://www.ihes.fr/ 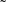
carbone/data10.

### Alignment Positions Versus PDB Positions for Describing Coevolved Residues

In Texts S2, S4, S7, S8, S9, S11, S15 and S16, coevolving blocks are described through residue positions in the PDB structure for the Amyloid, MukB and Protein A families. For the AATPase subfamilies, we considered alignment positions. The mapping between PDB positions and alignment positions is reported in Text S15.

### Tree Construction

Trees were constructed from alignments with PHYML [75]. They have been rooted manually by combining the pair of rooted subtrees with smaller distance that is issued by PHYML.

### Motifs Extension and New Motifs

Given a known motif, we extend it on the left and on the right by identifying residues that are coevolving and lie at 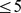 positions away from the original motif. All coevolving positions that are adjacent to the ones already detected will be included in the extension. New motifs are defined similarly by grouping coevolving residues lying at 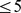 positions away from each other. All adjacent coevolving positions will be included as above. Notice that the handmade alignment of [56] is realized on subdomains (obtained by removing large insertions; subdomains are separated by fully gapped positions in Text S6). Motifs extension and novel identification are realized within each subdomain.

### BIS Based on Physico-chemical Properties

BIS analysis, either based on blocks or on residues, can be realized by considering physico-chemical amino acid properties. To do this, BIS transforms protein sequences into a simplified language of 8 letters VDKYNCGP, where each letter corresponds to a distinguished class: hydrophobic (VILMFWA), negatively charged (DE), positively charged (KR), aromatic (YH), polar (NSTQ), and C, G, P are considered as special. The amino acid classification is suggested in [63] according to biochemical similarities. BIS is then applied to protein families defined in the simplified language.

### Evaluation of Performance

To evaluate BIS (and other systems) performance on a given protein we rely on the following quantities: the number of residues correctly predicted as (structurally or functionally) important (true positives, TP), the number of residues correctly predicted as non-important (true negatives, TN), the number of non-important residues predicted as important (false positives, FP) and the number of important residues predicted as non-important (false negatives, FN). All experimentally validated residues known to play a structural or functional role for the protein constitute the set of important residues. Since the set is likely not to be complete, the evaluation likely underestimates the performance of the predictive system. Aware of this, we use four standard measures of performance: sensitivity 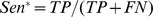, specificity 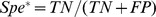, accuracy 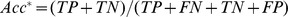 and positive predictive value 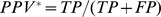, where the * symbol highlights the fact that important residues are the experimentally identified ones.

To compute the probability of predicting TP residues out of the set of experimentally confirmed residues (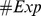) by selecting the number of residues identified by coevolution analysis (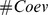) for an alignment length 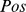, we used the following formula:
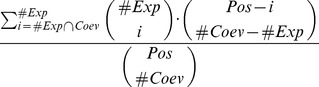
where 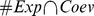 is the number of residues that are both predicted as coevolving and experimentally identified.

### Comparison with Other Systems

BIS is compared to several coevolution analysis systems: Statistical Coupling Analysis (SCA) [12] (we used the SCA implementation in [64], downloaded at http://coevolution.gersteinlab.org/coevolution/) where two functions (DB and TM) are proposed to compute a “symmetric score” and we compared to both of them; Explicit Likelihood of Subset Variation (ELSC) [66] (implementation in [64]); Mutual Information (MI) [8] (implementation in [64]); Maximal SubTree method (MST) [19] (downloaded at http://www.ihes.fr/ 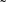
carbone/data7/MaxSubTree.tgz and run with default values for its parameters). The large-scale analysis CTMP method [16] was also run (Text S16). For comparison, coevolution score matrices issued by the methods were clustered with CLAG [74]. For MukB, Protein A and AATPase proteins analysis, CLAG clusters were obtained by setting stringent parameter values, 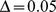 and environmental and symmetric scores 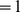. For the Amyloid analysis, CLAG parameters were 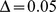 and environmental and symmetric scores 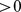. The same parameters have been used for all coevolution analysis methods.

Comparison with systems for conservation analysis is done online with: ET Viewer 2.0.

(http://mammoth.bcm.tmc.edu/) [50], [51], Consurf (http://consurf.tau.ac.il/; conserved residues have rank 8 and 9) [49] and Rate4Site (http://www.tau.ac.il/itaymay/cp/rate4site.html with score for conserved positions 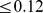) [65].

Performance of BIS and the systems above has been evaluated by using Sen^*^, Spe^*^, Acc^*^, PPV^*^. We say that a system is better than another when it performs better over at least three of the four performance measures. If BIS performs better over only two such measures, then we compute the sum of the differences for all measures (that is 
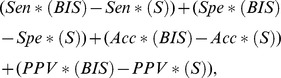
where 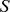 is one of the 8 systems) and evaluate BIS as successful only if the sum is positive. We say that two systems have comparable performance if the absolute value of the sum of the differences and of each difference is 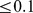.

## Supporting Information

Figure S1
**Information content of proteins considered in the article.** The value 0 corresponds to maximal conservation. The color scales are normalized on IC min and max values, where the maximum value varies from protein to protein. Amino-acids that are not considered in BIS analysis are colored black on the structure. Top: Amyloid beta peptide (for the alignment of 80 sequences); middle left: MukB protein (for the alignment of 200 sequences); middle right: protein A - B domain (for the alignment of 452 sequences); bottom left: Upf1 helicase subfamily; bottom right: Ski2-like helicase subfamily. We recall that given an alignment for a protein family and a position 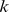, the Information Content of 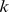 is defined as 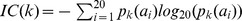 where 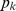 is the frequency of amino acid 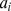 at position 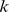. 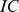 values span from 0 to at most 1, where 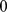 corresponds to a fully conserved position and 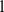 to a uniform amino-acids occurrence.(TIF)

Table S1
**Summary of BIS performance compared to other methods.** A summary of the BIS performance with respect to all other methods for co-evolution and conservation analysis considered in the article. For each method, we evaluate whether BIS performs better than another system with respect to criteria that combine Sensibility, Specificity, Accuracy and Positive Predictive Value of the performance. The criteria are introduced in the article. The symbol “ = ” denotes equal performance and the symbol “
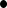
” denotes that the system performs better than BIS. No symbol indicates BIS better performance.(TEX)

Text S1 Analysis of conserved and gapped positions in Pfam families.
(PDF)

Text S2 Comparison of coevolution analysis methods on aligned sets of sequences representing the MukB protein family.
(PDF)

Text S3 MukB supplementary figures.
(PDF)

Text S4 Comparison of coevolution analysis methods on aligned sets of sequences representing the Protein A - B domain protein family.
(PDF)

Text S5 Analysis of SF motifs and extended SF motifs after coevolution analysis of the AATPase families.
(PDF)

Text S6 AATPase supplementary figures.
(PDF)

Text S7 BIS coevolution analysis based on physico-chemical properties.
(PDF)

Text S8 Comparison of coevolution analysis methods on aligned sets of sequences representing AATPase families.
(PDF)

Text S9Comparison of coevolution analysis methods on aligned sets of sequences representing the Amyloid 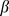-peptide.(PDF)

Text S10 Validation of performance of different coevolution analysis methods.
(PDF)

Text S11 Conservation analysis of the protein families analyzed in the article.
(PDF)

Text S12 Comparison between BIS analysis based on exceptions and BIS analysis on all hits.
(PDF)

Text S13 Instructions for running BIS.
(PDF)

Text S14 Systems of coevolution and conservation analysis used for comparison, sets of aligned sequences, and clustering.
(PDF)

Text S15 Mapping between PDB positions and alignment positions.
(PDF)

Text S16 BIS and CTMP comparison.
(PDF)

Text S17 Protein A-B domain supplementary figures.
(PDF)
