# Supplementary figures and images for: Protein Fragments: Functional and Structural Roles of Their Coevolution Networks
Source: PLoS One. 2012 Nov 5;7(11):e48124. doi: 10.1371/journal.pone.0048124 (PMC3489791; doi:10.1371/journal.pone.0048124)

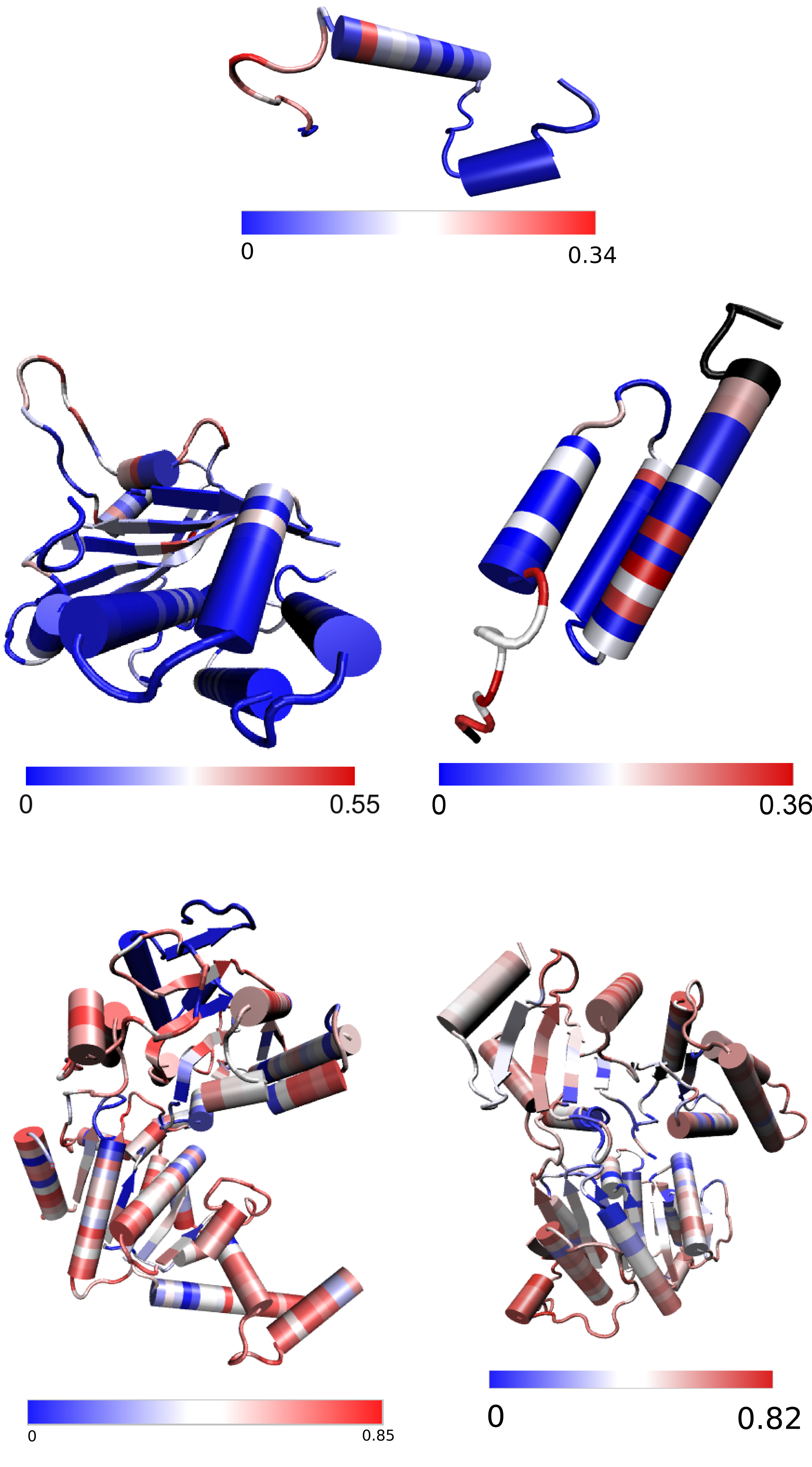

Supplement: Figure S1 — Information content of proteins considered in the article. The value 0 corresponds to maximal conservation. The color scales are normalized on IC min and max values, where the maximum value varies from protein to protein. Amino-acids that are not considered in BIS analysis are colored black on the structure. Top: Amyloid beta peptide (for the alignment of 80 sequences); middle left: MukB protein (for the alignment of 200 sequences); middle right: protein A - B domain (for the alignment of 452 sequences); bottom left: Upf1 helicase subfamily; bottom right: Ski2-like helicase subfamily. We recall that given an alignment for a protein family and a position , the Information Content of is defined as where is the frequency of amino acid at position . values span from 0 to at most 1, where corresponds to a fully conserved position and to a uniform amino-acids occurrence. (TIF) [file pone.0048124.s001.tif]
