# Supplementary material for: Protein Fragments: Functional and Structural Roles of Their Coevolution Networks
Source: PLoS One. 2012 Nov 5;7(11):e48124. doi: 10.1371/journal.pone.0048124 (PMC3489791; doi:10.1371/journal.pone.0048124)
Supplement: Text S1 — Analysis of conserved and gapped positions in Pfam families. (PDF) [file pone.0048124.s003.pdf]

Supporting Information Text S1: Analysis of conserved and gapped positions in Pfam

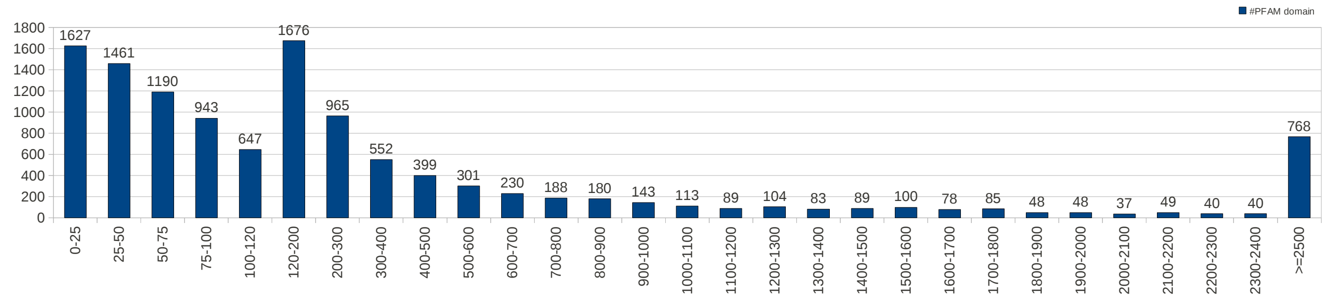

Figure 1: **Distribution of Pfam families: number of sequences**.
Distribution of Pfam families sizes, where the size of a family is the number of non identical sequences (x-axis). The number of families is given in the y-axis. The analysis has been done on PFAM database v25.

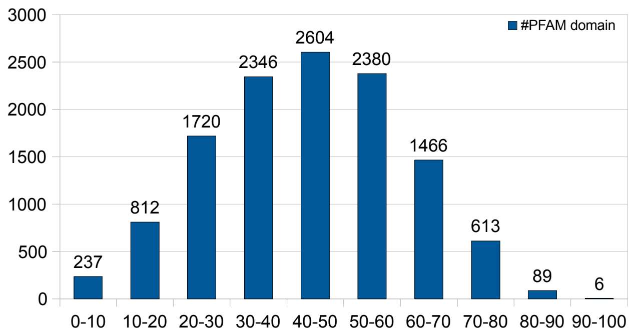

Figure 2: **Distribution of Pfam families: high conservation and high number of gaps**.
Distribution of Pfam families containing a percentage of positions which are neither gapped nor highly conserved (x-axis). A highly conserved position in a sequence alignment is such that at least the 75% of sequences in the alignment contain the same amino-acid. A gapped position is such that at least 60% of sequences contain a gap. The number of families is given in the y-axis. The analysis has been done on PFAM database v25.

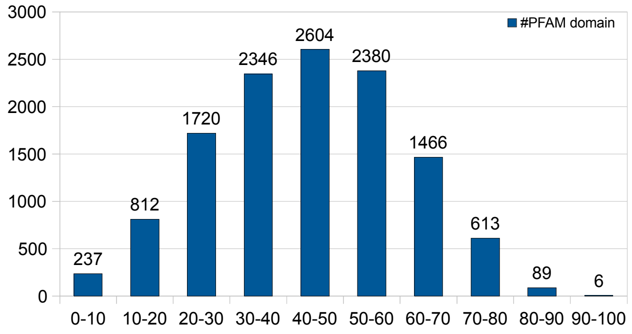

Figure 3: **Distribution of Pfam families: high conservation and high number of gaps**. Distribution of Pfam families containing a percentage of positions which are neither gapped nor highly conserved (x-axis). A highly conserved position in a sequence alignment is such that at least the 75% of sequences in the alignment contain the same amino-acid. A gapped position is such that at least 60% of sequences contain a gap. The number of families is given in the y-axis. The analysis has been done on PFAM database v25.

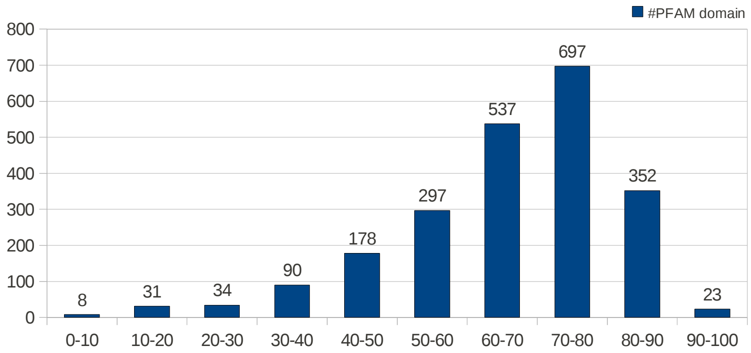

Figure 4: **Number of highly gapped positions in Pfam families with more than 1000 sequences**. Distribution of Pfam families containing a percentage of positions which are highly gapped (x-axis), that is at least 60% of sequences contain a gap at that position. The number of families is given in the y-axis. The analysis has been done on PFAM database v25.

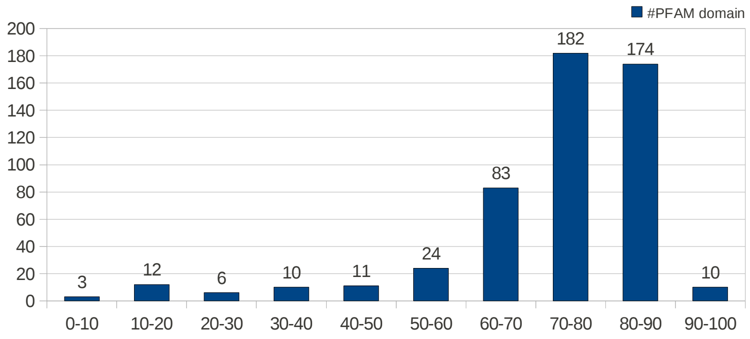

Figure 5: **Number of highly gapped positions in Pfam families with more than 5000 sequences**. Distribution of Pfam families containing a percentage of positions which are highly gapped (x-axis), that is at least 60% of sequences contain a gap at that position. The number of families is given in the y-axis. The analysis has been done on PFAM database v25.
