# Supplementary material for: Protein Fragments: Functional and Structural Roles of Their Coevolution Networks
Source: PLoS One. 2012 Nov 5;7(11):e48124. doi: 10.1371/journal.pone.0048124 (PMC3489791; doi:10.1371/journal.pone.0048124)
Supplement: Text S2 — Comparison of coevolution analysis methods on aligned sets of sequences representing the MukB protein family. (PDF) [file pone.0048124.s004.pdf]

Supporting Information Text S2: comparison of coevolution analysis methods on aligned sets of sequences representing the MukB protein family

Methods comparison - MukB on 200 sequences				
Method	Al	Cl	Positions	Sym
BIS, *pos*, $d = 0$	200	C1	4 6 9	1
		C2	14 15 16 17 18 19 20 22 23 26	1
		C3	**34 35 36 37 39 40 41** 42	1
BIS, *pos*, $d = 1$	200	C1	44 45 52 53 54 57 58 60 61 62 63 64 65 66 67 72 73 74 76 77 79 80 81 84 86 87 94 95 99 108 112 113 115 116 121 175 179 182 189 193 194 198 200 202 203 204 205 206 207 208 209 210 213 216 217 218 220 221 222 223 225 227	1
		C2	98 174	1
		C3	107 111 117 214 219	1
		C4	119 125	1
		C5	59 133	1
		C6	49 145 170	1
		C7	55 187	1
		C8	29 30 31 32 33	1
BIS, *blocks*, $d = 0$	200	C1	4 6 9	1
		C2	14-20 22-23 26	1
		C3	**34-37 39-41**-42	1
BIS, *blocks*, $d = 1$	200	C1	57-67 133	1
		C2	44-45 52-54 57-58 60-67 72-74 76-77 79-81 84 86-87 94-95 99 108 112-113 115-116 121 175 179 182 189 193-194 198 200 202-210 213 216-218 220-223 225 227	1
		C3	98-99 174-175	1
		C4	107-108 111-113 115-117 213-214 216-223	1
		C5	119 125	1
		C6	49 145 170	1
		C7	52-55 187	1
		C8	29 30 31 32 33	1
ELSC	200	C1	**107 111**	0.8
		C2	**214 219**	0.8
		C3	**44 45 52 53 54 57 58 77 79 80 81 84 86 87 94 95 99 108 112 113 115 116 121 175 179 182 189 193 194 198 200 202 203 204 205 206 207 208 209 210 213 216 217 218 220 221 222 223 225**	0.5
		C4	**4 6 9**	0.5
		C5	**29 30 31 32 33 34 35 36 37 39 40 41 42**	0.5
		C6	**14 15 16 17 18 19 20 22 23 26**	0.5

Table 1: **MukB protein: comparing methodologies run over 200 sequences. Part 1.** For each method, we report the clusters of co-evolving residues detected by the clustering algorithm CLAG (with environmental score equal 1, symmetric score > 0 and $\Delta = 0.05$). For BIS we selected clusters with symmetric score $= 1$ only. For each methodology we report the number of sequences (Al), the names of the clusters (Cl), the positions on the PDB structure (Positions). Results are reported for BIS run on blocks and on single alignment positions. SCA, ELSC, MI, MST and CTMP methods are compared to BIS execution when considering alignment positions (*pos*). Common residues predicted by both methodologies are highlighted in bold. Green residues are those belonging to the Walker-A. NO MATRIX means that the method did not output any co-evolution pair; NO CLUSTERS means that the method output co-evolution pairs that could not be clustered together.

**Methods comparison - MukB on 200 sequences**				
Method	Al	Cl	Positions	Sym
SCA-DB	200	C1	**119 125**	0.9
		C2	**98 174**	0.9
		C3	**107 111 117 214 219**	0.9
		C4	**49 145 170**	0.9
		C5	**55 187**	0.9
		C6	103 224	0.9
		C7	47 90 154 196	0.8
		C8	188 211	0.8
		C9	**34 35 36 37 39 40 41 42**	0.3
		C10	**44 45 52 53 54 57 58 77 79 80 81 84 86 87 94 95 99 108 112 113 115 116 121 175 179 182 189 193 194 198 200 202 203 204 205 206 207 208 209 210 213 216 217 218 220 221 222 223 225**	0.3
		C11	**29 30 31 32 33**	0.3
		C12	**14 15 16 17 18 19 20 22 23 26**	0.3
		C13	**4 6 9**	0.3
SCA-TM	200	C1	**55 187**	0.9
		C2	**98 174**	0.8
		C3	**107 111 117 214 219**	0.8
		C4	**49 145 170**	0.8
		C5	**119 125**	0.8
		C6	47 90 154 196	0.7
		C7	188 211	0.7
		C8	**44 45 52 53 54 57 58 77 79 80 81 84 86 87 94 95 99 108 112 113 115 116 121 175 179 182 189 193 194 198 200 202 203 204 205 206 207 208 209 210 213 216 217 218 220 221 222 223 225**	0.2
		C9	**14 15 16 17 18 19 20 22 23 26**	0.2
		C10	**4 6 9**	0.2
		C11	**34 35 36 37 39 40 41 42**	0.2
		C12	**29 30 31 32 33**	0.2
MI	200	C1	122 128 141	1
		C2	128 141	1
		C3	122 141	1
		C4	**55 187**	0.9
		C5	**119 125**	0.8
		C6	47 90 154 196	0.7
		C7	188 211	0.7
		C8	**98 174**	0.7
		C9	**49 145 170**	0.7
		C10	**107 111 117 214 219**	0.7
MST	200	NO MATRIX		
CTMP	200	NO MATRIX		

Table 2: **MukB protein: comparing methodologies run over 200 sequences. Part 2.**
See legend of Table 2-Text S2.

Methods comparison - MukB on 49 sequences				
Method	Al	Cl	Positions	Sym
BIS, *pos*, $d = 0$	49	C1	4 6 9	1
		C2	14 15 16 17 18 19 20 22 23 26	1
		C3	**36 37 39 40 41** 42	1
BIS, *pos*, $d = 1$	49	C1	44 45 52 53 54 57 58 60 61 62 63 64 65 66 67 72 73 74 76 77 79 80 81 84 86 87 94 95 99 106 108 112 113 115 116 121 175 179 182 189 193 194 198 200 202 203 204 205 206 207 208 209 210 213 216 217 218 220 221 222 223 225 227	1
		C2	98 174	1
		C3	107 111 117 214 219	1
		C4	119 125	1
		C5	59 133	1
		C6	55 187	1
		C7	29 30 31 32 33	1
BIS, *blocks*, $d = 0$	49	C1	4 6 9	1
		C2	14-20 22-23 26	1
		C3	**36-37 39-41**-42	1
BIS, *blocks*, $d = 1$	49	C1	57-67 133	1
		C2	44-45 52-54 57-58 60-67 72-74 76-77 79-81 84 86-87 94-95 99 106 108 112-113 115-116 121 175 179 182 189 193-194 198 200 202-210 213 216-218 220-223 225 227	1
		C3	98-99 174-175	1
		C4	106-108 111-113 115-117 213-214 216-223	1
		C5	119 125	1
		C6	52-55 187	1
		C7	29 30 31 32 33	1
ELSC	49	C1	**107 111**	0.9
		C2	**214 219**	0.9
		C3	**44 45 52 53 54 57 58 77 79 80 81 84 86 87 94 95 99 106 108 112 113 115 116 121 175 179 182 189 193 194 198 200 202 203 204 205 206 207 208 209 210 213 216 217 218 220 221 222 223 225**	0.6
		C4	**14 15 16 17 18 19 20 22 23 26**	0.6
		C5	**4 6 9**	0.6
		C6	**29 30 31 32 33 34 35 36 37 39 40 41 42**	0.6

Table 3: **MukB protein: comparing methodologies run over 49 sequences. Part 1.**
See legend of Table 2-Text S2.

Methods comparison - MukB on 49 sequences				
Method	Al	Cl	Positions	Sym
SCA-DB	49	C1	**119 125**	0.9
		C2	**107 111 117 214 219**	0.9
		C3	47 90 154 196	0.9
		C4	**98 174**	0.9
		C5	188 211	0.9
		C6	49 145 170	0.9
		C7	132 **133**	0.9
		C8	**44 45 52 53 54 57 58 77 79 80 81 84 86 87 94 95 99 106 108 112 113 115 116 121 175 179 182 189 193 194 198 200 202 203 204 205 206 207 208 209 210 213 216 217 218 220 221 222 223 225**	0.3
		C9	**4 6 9**	0.3
		C10	**34 35 36 37 39 40 41 42**	0.3
		C11	**29 30 31 32 33**	0.3
		C12	**14 15 16 17 18 19 20 22 23 26**	0.3
SCA-TM	49	C1	154 196	0.9
		C2	47 90	0.9
		C3	188 211	0.9
		C4	49 145 170	0.9
		C5	**98 174**	0.9
		C6	**107 117 219**	0.9
		C7	**111 214**	0.9
		C8	132 **133**	0.9
		C9	**44 45 52 53 54 57 58 77 79 80 81 84 86 87 94 95 99 106 108 112 113 115 116 121 175 179 182 189 193 194 198 200 202 203 204 205 206 207 208 209 210 213 216 217 218 220 221 222 223 225**	0.2
		C10	**34 35 36 37 39 40 41 42**	0.2
		C11	**4 6 9**	0.2
		C12	**29 30 31 32 33**	0.2
		C13	**14 15 16 17 18 19 20 22 23 26**	0.2
MI	49	C1	105 137	0.9
		C2	**55 187**	0.9
		C3	**98 174**	0.8
		C4	**107 111 117 214 219**	0.8
		C5	47 90 154 196	0.7
		C6	188 211	0.7
		C7	49 145 170	0.7
MST	49	NO MATRIX		
CTMP	49	NO CLUSTERS		

Table 4: **MukB protein: comparing methodologies run over 49 sequences. Part 2.**
See legend of Table 2-Text S2.

Methods comparison - MukB on 205 sequences				
Method	Al	Cl	Positions	Sym
BIS, *pos*, $d=0$	205	C1	16 20 22	1
		C2	**36 37 39 40**	1
BIS, *pos*, $d=1$	205	C1	45 61 64 66 77 200 202 203	1
		C2	44 60 62 76 84 204 210 222	1
		C3	54 74 81 99 179 182 207 217 221	1
		C4	80 205	1
		C5	52 193 220	1
		C6	31 33	1
BIS, *blocks*, $d=0$	205	C1	16-17 20 22	1
		C2	**36-37 39-40**	1
BIS, *blocks*, $d=1$	205	C1	44 60 62 76 84 204 210 221-222	1
		C2	44-45 60-62 64 66 76-77 200 202-205	1
		C3	54 74 81 99 179 182 207 217 221	1
		C4	80-81 205	1
		C5	52 193 220-221	1
		C6	31 33	1
ELSC	205	C1	**200 202 203**	0.9
		C2	**31 33**	0.9
		C3	**204 210**	0.8
		C4	208 209	0.8
		C5	15 19 26	0.8
		C6	**16 20 22**	0.7
		C7	32 **34 36 37 39 40**	0.2
		C8	**54 81 99 179 182 207 217 221**	0.2
SCA-DB	205	C1	**45** 57 **77** 94 113 175 189 198 **202 203** 206 216	0.9
		C2	113 175 189 198	0.9
		C3	94 113 189 206	0.9
		C4	**45** 94 113 189 **202 203** 206 216	0.9
		C5	**45** 94 113 189 **202 203** 216	0.9
		C6	**45 77** 94 113 189 **202 203** 216	0.9
		C7	**80 205** 223	0.9
		C8	**31 33**	0.9
		C9	**77** 113 189 **202** 216	0.9
		C10	57 113 189	0.9
		C11	19 26	0.8
		C12	209 218	0.8
		C13	208 209 218	0.8
		C14	208 218	0.8
		C15	79 **84 204 210 222**	0.8
		C16	**45** 57 **77 202 203** 216	0.8
		C17	57 **202**	0.8
		C18	57 216	0.8
		C19	**45** 57 **77 203**	0.8
		C20	**45** 57 **77**	0.8
		C21	57 **77 203**	0.8
		C22	**52 193 220**	0.8
		C23	**16 20 22**	0.6
		C24	53 121	0.6
SCA-TM	205	C1	113 189 **202**	0.8
		C2	108 113 **202**	0.8
		C3	108 113	0.8
		C4	175 189 **202**	0.8
		C5	94 175 189	0.8
		C6	94 175	0.8
		C7	**44** 79 **84 204 210**	0.7
		C8	**45 77**	0.7
		C9	57 **200 203**	0.7
		C10	**20 22**	0.5

Table 5: **MukB protein: comparing methodologies run over 205 sequences. Part 1.** See legend of Table 2-Text S2. Continued in Table 6-Text S2.

Methods comparison - MukB on 205 sequences				
Method	Al	Cl	Positions	Sym
MI	205	C1	35 223	0.8
		C2	18 108	0.7
		C3	189 198	0.7
		C4	57 94 113 175 216	0.7
		C5	11 23 **31 33 45 77 200 202 203**	0.7
		C6	41 **80 205**	0.7
		C7	15 19	0.7
		C8	**44 84 204 210 222**	0.6
		C9	**52 193 220**	0.6
		C10	208 209 218	0.6
		C11	42 194	0.5
		C12	9 **16 20 22** 115 213	0.5
		C13	53 121	0.2
MST	205	C1	**31** 32	1
		C2	189 198	1
		C3	**80 205**	1
		C4	53 121	1
		C5	**45 61 64 66 77 200** 201 **203**	1
		C6	10 **16 22**	1
		C7	**36 37 39 40**	1
		C8	32 34	1
		C9	**54 74 81 99 179 182 207 217 221**	1
		C10	**52 193 220**	1
		C11	**44 60 62** 67 **76 84 204** 208 209 **210** 211 218	1
CTMP	205	NO MATRIX		

Table 6: **MukB protein: comparing methodologies run over 205 sequences. Part 2.**
Table 5-Text S2 continued.

Methods comparison - MukB on 54 sequences				
Method	Al	Cl	Positions	Sym
BIS, *pos*, $d = 0$	54	C1	16 20 22	1
		C2	**36 37 39 40**	1
BIS, *pos*, $d = 1$	54	C1	45 61 64 66 77 200 202 203	1
		C2	44 60 62 76 84 204 210 222	1
		C3	54 74 81 99 179 182 207 217 221	1
		C4	80 205	1
		C5	52 193 220	1
		C6	31 33	1
BIS, *blocks*, $d = 0$	54	C1	16-17 20 22	1
		C2	**36-37 39-40**	1
BIS, *blocks*, $d = 1$	54	C1	44 60 62 76 84 204 210 221-222	1
		C2	44-45 60-62 64 66 76-77 200 202-205	1
		C3	54 74 81 99 179 182 207 217 221	1
		C4	80-81 205	1
		C5	52 193 220-221	1
		C6	31 33	1
ELSC	54	C1	15 19	0.9
		C2	**31 33**	0.9
		C3	**200 202 203**	0.9
		C4	208 209	0.9
		C5	**20 22**	0.8
SCA-DB	54	C1	117 219	0.9
		C2	**45** 57 **77 80** 94 108 113 175 189 198 **200 202 203 205** 206 216 223	0.9
		C3	86 225	0.9
		C4	18 23	0.9
		C5	**31 33**	0.9
		C6	**44** 79 **84 204** 208 209 **210** 218	0.9
		C7	**44** 79 **84** 106 **204** 208 209 **210** 218	0.9
		C8	**44** 58 79 **84** 106 **204** 208 209 **210** 218	0.9
		C9	79 **84** 106 **204** 208 209 **210** 218	0.9
		C10	19 26	0.9
		C11	15 19 26	0.9
		C12	58 209 218	0.9
		C13	15 19	0.9
		C14	**52 193 220**	0.9
		C15	**16 20 22**	0.8
		C16	58 79 **84** 106 **204** 208 209 **210** 218 **222**	0.8
		C17	58 79 **84** 106 **204** 208 **210 222**	0.8
		C18	58 **204 222**	0.8
		C19	218 **222**	0.8
		C20	209 **222**	0.8
		C21	58 **210 222**	0.8
		C22	58 208 **222**	0.8
		C23	58 **84 222**	0.8
		C24	58 79 **222**	0.8
		C25	58 106 **222**	0.8

Table 7: **MukB protein: comparing methodologies run over 54 sequences. Part 1.** See legend of Table 2-Text S2. Continued in Table 8-Text S2.

Methods comparison - MukB on 54 sequences				
Method	Al	Cl	Positions	Sym
SCA-TM	54	C1	117 219	0.9
		C2	86 225	0.9
		C3	**80** 94 108 113 175 189 **202**	0.9
		C4	**80** 94 108 113 175 189 198 **202** 223	0.9
		C5	**80** 94 108 113 175 189	0.9
		C6	**80** 94 108 113 175 189 198 206 223	0.9
		C7	**80** 94 108 113 **202**	0.9
		C8	94 108 175 198 216 223	0.9
		C9	175 206	0.9
		C10	198 216 223	0.9
		C11	**45 77 200 203 205**	0.9
		C12	**45 77 205**	0.9
		C13	**200 203 205**	0.9
		C14	18 23	0.9
		C15	58 79 **84** 106 **204** 208 209 **210 222**	0.8
		C16	58 106 209 **222**	0.8
		C17	**16 22**	0.8
		C18	**44 204** 208 **210**	0.8
		C19	**44** 79 **84 204** 208 209 **210**	0.8
		C20	79 **84 204** 208 209 **210**	0.8
MI	54	C1	18 108	0.9
		C2	189 198	0.9
		C3	57 94 113 216	0.9
		C4	35 223	0.9
		C5	11 **45 77 200 202 203**	0.9
		C6	11 **45**	0.9
		C7	11 **77**	0.9
		C8	11 **200**	0.9
		C9	11 **202**	0.9
		C10	11 **203**	0.9
		C11	11 23 **31 33 45 77 200 202 203**	0.8
		C12	23 **31 33 45 77 200 202 203**	0.8
		C13	11 23 **31 33**	0.8
		C14	41 **80 205**	0.8
		C15	15 19 79	0.7
		C16	106 208 209 218	0.7
		C17	**52 193 220**	0.6
		C18	**44 84 204 210 222**	0.6
		C19	9 **16 20 22** 213	0.5
		C20	9 **16**	0.5
		C21	9 **20**	0.5
		C22	9 **22**	0.5
		C23	9 213	0.5
		C24	42 194	0.4
		C25	**16 20 22** 213	0.4
		C26	53 121	0.1
MST	54	C1	**31 32**	0.90
		C2	63 206	0.90
		C3	**80 205**	0.90
		C4	**44 60 62 76 84 204 210 222**	0.90
		C5	**45 61 64 66 77 200 202 203**	0.90
		C6	**36** 38 **39 40**	0.90
		C7	**32 34**	0.90
		C8	**54 74 81 99 179 182 207 217 221**	0.90
CTMP	NO CLUSTERS			

Table 8: **MukB protein: comparing methodologies run over 54 sequences. Part 2.**
Table 7-Text S2 continued.
