# Supplementary material for: Protein Fragments: Functional and Structural Roles of Their Coevolution Networks
Source: PLoS One. 2012 Nov 5;7(11):e48124. doi: 10.1371/journal.pone.0048124 (PMC3489791; doi:10.1371/journal.pone.0048124)
Supplement: Text S3 — MukB supplementary figures. (PDF) [file pone.0048124.s005.pdf]

Supporting Information Text S3: MukB supplementary figures

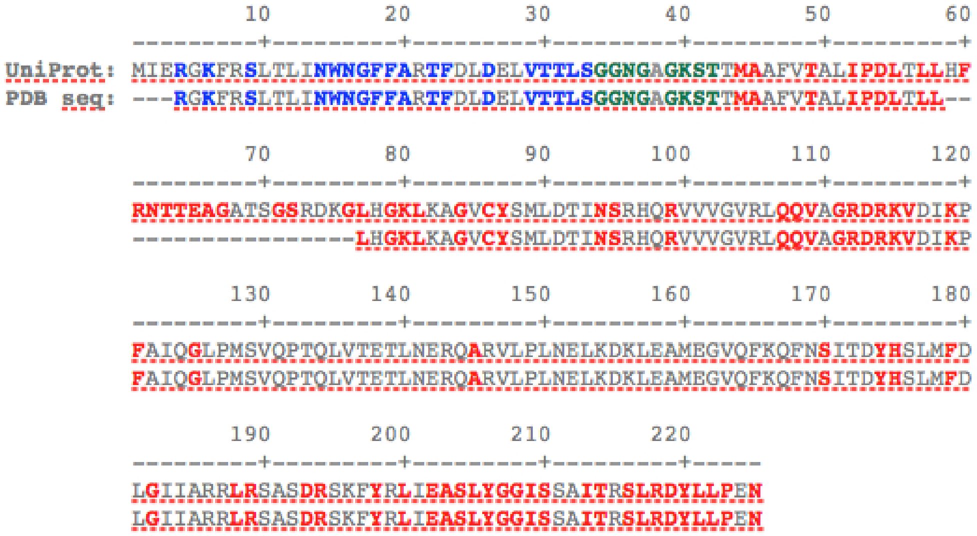

Figure 1: **MukB protein: partitions indicated on the sequence.**

Blue, green and red amino-acids correspond to MukB partitions obtained by running BIS (on blocks) over 200 sequences for $d = 0$ and $d = 1$ (with $\Delta = 0.05$) and highlighted in Fig. 3 in the main text. The pdb sequence (`1qhl:A`) is aligned against the UniProt sequence P22523 (*Escherichia coli* MukB).

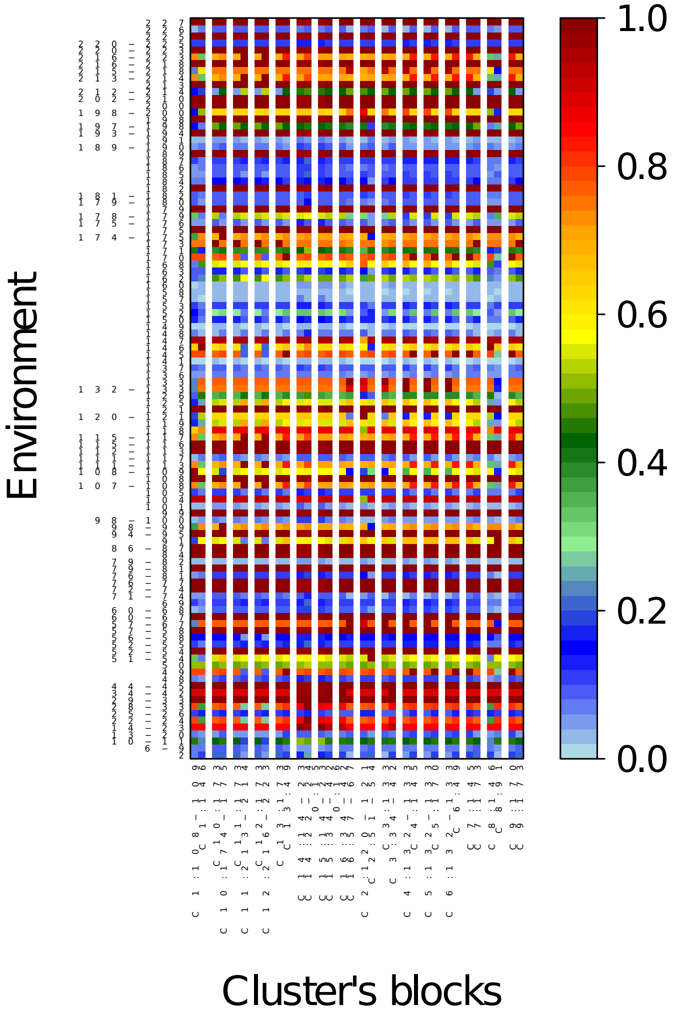

Figure 2: **MukB protein: clusters for $d = 1$ that are not clusters for d_1^+.**
Clusters of the MukB score matrix that are detected by CLAG for $d = 1$ but that are not detected for d_1^+ (that is, $d \leq 1$). (Notice that not all clusters for $d = 1$ are reported.) This is due to the environment (that is, the set of blocks) for $d = 1$ that differs from the one for $d \leq 1$. Recall that the environment for $d \leq 1$ contains all blocks for $d = 0$ and all blocks for $d = 1$.

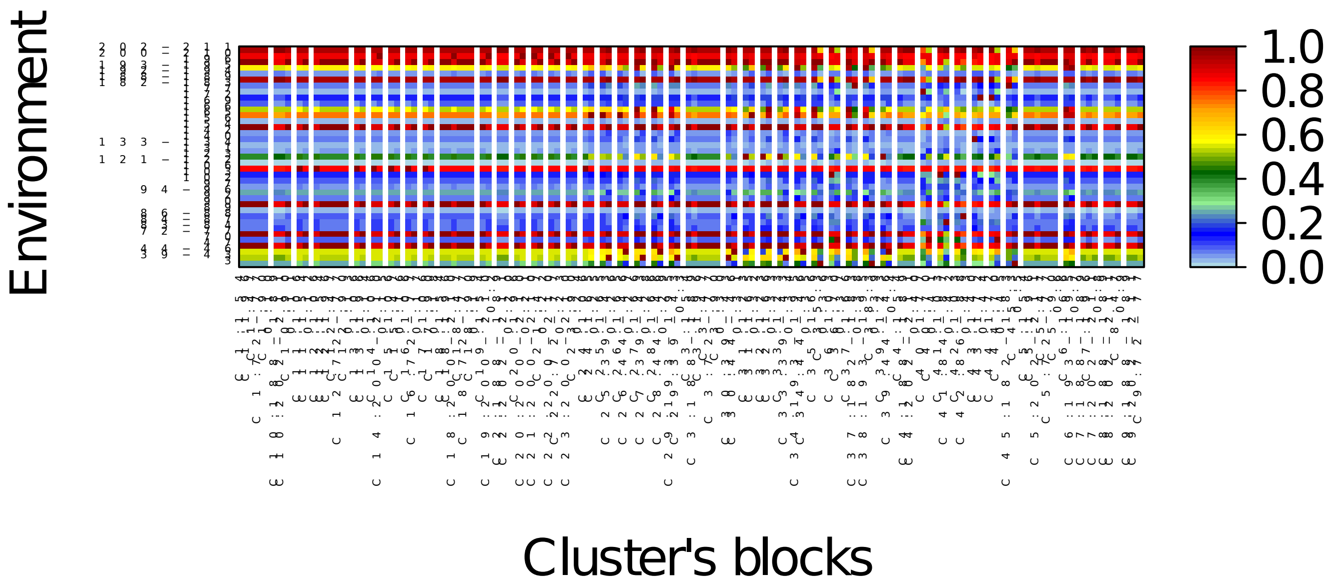

Figure 3: **MukB protein: clusters for d_1^+ that are not clusters for $d = 1$.**
See legend in Fig. 2-Text S3.
