# Supplementary material for: Protein Fragments: Functional and Structural Roles of Their Coevolution Networks
Source: PLoS One. 2012 Nov 5;7(11):e48124. doi: 10.1371/journal.pone.0048124 (PMC3489791; doi:10.1371/journal.pone.0048124)
Supplement: Text S4 — Comparison of coevolution analysis methods on aligned sets of sequences representing the Protein A - B domain protein family. (PDF) [file pone.0048124.s006.pdf]

Supporting Information Text S4: comparison of coevolution analysis methods on aligned sets of sequences representing the Protein A - B domain protein family

Methods comparison - Protein A-B domain on 452 sequences				
Method	Al	Cl	Positions	Sym
BIS, *pos*, $d = 0$	452	C1	22 23 27 29	1
		C2	30 31 32 33 34 35 36 37 38 39 40 42 46 48 49	1
		C3	41 44	1
		C4	51 52	1
BIS, *pos*, $d = 1$	452	C1	11 13 14 15 18	1
		C2	16 17	1
BIS, *blocks*, $d = 0$	452	C1	22-23 27 29	1
		C2	29-40 42 46 48-49	1
		C3	29-42 44	1
		C4	51 52	1
BIS, *blocks*, $d = 1$	452	C5	16 17	1
		C6	11 13-15 18	1
ELSC	452	S1	**16 17**	0.90
		S2	6 8	0.90
		S3	19 20	0.81
		S4	20 24	0.72
		S5	19 20 24	0.72
		S6	19 24	0.72
		S7	**11 13 14 15 18**	0.36
		S8	**22 23 27 29 30 31 32 33 34 35 36 37 38 39 40 42 46 48 49 51 52**	0.36
SCA-DB	452	S1	19 20	0.9
		S3	4 6 7	0.9
		S4	4 6 7 **17**	0.9
		S5	4 16 **17**	0.9
		S6	**16 17**	0.9
		S8	**41 44**	0.9
		S9	3 9	0.9
		S10	16 50	0.8
		S11	**30 31 32 33 34 35 36 37 38 39 40 42 46 48 49**	0.1
		S12	**51 52**	0.1
SCA-TM	452	S1	3 9	1
		S2	6 50	0.9
		S3	20 60	0.9
		S4	**41 44**	0.9
		S5	4 7	0.9
		S6	4 7 **16**	0.9
		S7	7 16	0.9
		S8	**30 31 32 33 34 35 36 37 38 39 40 42 46 48 49**	0
		S9	**51 52**	0
MI	452	S1	6 7 8	1
		S2	21 **30 33 34 38 46 49**	0.61
		S3	**36 23**	0.42
		S4	**27 35**	0.42
MST	452	S1	**22 23 27 29**	1
		S2	**16 17**	1
		S3	**11 13 14 15 18**	1
		S4	7 25	0.82
		S5	6 8 10	0.82
		S6	6 8	0.82
		S7	6 4	0.82
CTMP	452	NO CLUSTERS		

Table 1: **Protein A-B domain: comparing methodologies run over 452 sequences.**
For each method, we report the clusters of co-evolving residues detected by the clustering algorithm CLAG (with environmental score equal 1, symmetric score > 0 and $\Delta = 0.05$). For BIS we selected clusters with symmetric score $= 1$ only. For each methodology we report the number of sequences (Al), the names of the clusters (Cl), the positions on the PDB structure (Positions). Results are reported for BIS run on blocks and on single alignment positions. SCA, ELSC, MI, MST and CTMP methods are compared to BIS execution when considering alignment positions (*pos*). Common residues predicted by both methodologies are highlighted in bold. Green residues are those belonging to the Walker-A. NO MATRIX means that the method did not output any co-evolution pair; NO CLUSTERS means that the method output co-evolution pairs that could not be clustered together.

Methods comparison - Protein A-B domain on 490 sequences				
Method	Al	Cl	Positions	Sym
BIS, *pos*, $d = 0$	490	C1	32 39	1
BIS, *pos*, $d = 1$	490	C1	11 13 14	1
BIS, *blocks*, $d = 0$	490	C1	32 39	1
BIS, *blocks*, $d = 1$	490	C2	11 13-14	1
ELSC	490	S1	30 33	0.61
SCA-DB	490	S1	3 9 40	1
		S2	30 33 34 38 40	0.8
		S3	30 33 34 38 40 46	0.8
		S4	30 33 38 40 46	0.8
		S5	49 53	0.7
		S6	22 42	0.7
		S7	47 35	0.7
		S8	23 36	0.7
SCA-TM	490	S1	3 9	1
		S2	33 38	0.71
		S3	30 34 40	0.61
		S4	30 34 49 46	0.61
		S5	30 46	0.61
		S5	22 24	0.52
MI	490	S1	20 19	1
		S2	16 17 25	0.90
		S3	7 6 16 17 25	0.90
		S4	6 16 17	0.90
		S5	41 44	0.90
		S6	43 60	0.71
		S7	**11 13 14** 15 18	0.33
		S8	22 23 27 29	0.33
		S9	30 31 32 33 34 35 36 37 38 39 40 42 46 48 49	0.33
MST	490	S1	**11 13 14**	1
		S2	8 6	0.90
		S3	15 18	0.90
		S4	22 23	0.90
CTMP	490	NO CLUSTERS		

Table 2: **Protein A-B domain: comparing methodologies run over 490 sequences.** See legend of Table 1-Text S4.

Methods comparison - Protein A-B domain on 28 sequences				
Method	Al	Cl	Positions	Sym
BIS, *pos*, $d = 0$	28	C1	32 39	1
BIS, *pos*, $d = 1$	28	C1	11 13 14	1
BIS, *blocks*, $d = 0$	28	C1	32 39	1
BIS, *blocks*, $d = 1$	28	C1	11 13-14	1
ELSC	28	S1	30 33	0.9
		S2	**11 13 14**	0
		S2	**32 39**	0
SCA-DB	28	S1	11 13 14	0.3
		S1	32 39	0.3
SCA-TM	28	S1	**11 13 14**	0.3
		S2	**32 39**	0.3
MI	28	S1	20 19	1
		S2	16 17 25	0.9
		S3	6 7 16 17 25	0.9
		S4	6 16 17 25	0.9
		S5	41 44	0.7
		S6	**11 13 14** 18	0.3
		S7	22 23 27 29	0.3
		S8	30 31 32 33 34 35 36 37 38 39 40 42 46 48 49	0.3
MST	28	S1	**11 13 14**	1
		S2	**32 39**	0.80
CTMP	28	NO CLUSTERS		

Table 3: **Protein A-B domain: comparing methodologies run over 28 sequences.**
See legend of Table 1-Text S4.

Methods comparison - Protein A-B domain on 20 sequences				
Method	Al	Cl	Positions	Sym
BIS, *pos*, $d = 0$	20	C1	22 23 27 29	1
		C2	30 31 32 33 34 35 36 37 38 39 40 42 46 48 49	1
		C3	41 44	1
		C4	51 52	1
BIS, *pos*, $d = 1$	20	C1	11 13 14 15 18	1
		C2	16 17	1
BIS, *blocks*, $d = 0$	20	C1	22-23 27 29	1
		C2	29-40 42 46 48-49	1
		C3	29-42 44	1
		C3	51 52	1
BIS, *blocks*, $d = 1$	20	C1	11 13-15 18	1
		C2	16 17	1
		S1	**16 17**	1
		S2	**41 44**	0.90
ELSC	20	S3	**11 13 14 15 18**	0.61
		S4	**22 23 27 29 30 31 32 33 34 35 36 37 38 39 40 42 46 48 49 51 52**	0.61
SCA-DB	20	NO CLUSTERS		
SCA-TM	452	NO MATRIX		
MI	20	S1	**23** 36	0.2
		S1	**22 23 27 29**	1
MST	20	S2	**11 13 14 15 18**	1
		S3	**16 17**	1
		S4	8 4	0.63
CTMP	20	NO CLUSTERS		

Table 4: **Protein A-B domain comparing methodologies run over 20 sequences.**
See legend of Table 1-Text S4.
