# Supplementary material for: Protein Fragments: Functional and Structural Roles of Their Coevolution Networks
Source: PLoS One. 2012 Nov 5;7(11):e48124. doi: 10.1371/journal.pone.0048124 (PMC3489791; doi:10.1371/journal.pone.0048124)
Supplement: Text S5 — Analysis of SF motifs and extended SF motifs after coevolution analysis of the AATPase families. (PDF) [file pone.0048124.s007.pdf]

Supporting Information Text S5: analysis of SF motifs and extended SF motifs after coevolution analysis of the AATPase families

SF motifs			
SF family	# residues in SF motifs	# co-evolving positions	Ratio
Upf1	18	26	0.69
RecD	32	73	0.43
UvrD/Rep	30	38	0.78
Rad3	28	62	0.45
DEAD-box	16	16	1
RecQ	31	42	0.73
Ski2-like	22	35	0.62
RigI-like	40	104	0.38
DEAH-RHA	29	43	0.67
NS3/NPH-II	36	101	0.35
Swi2/Snf2	14	15	0.93
**Extended SF motifs**			
Upf1	23	26	0.88
RecD/Pif1	50	73	0.68
UvrD	37	38	0.97
Rad3	52	62	0.83
DEAD-box	16	16	1
RecQ	39	42	0.92
Ski2-like	26	35	0.74
RigI-like	70	104	0.67
DEAH-RHA	41	43	0.95
NS3/NPH-II	72	101	0.71
Swi2/Snf2	15	15	1

Table 1: **AATPase subfamilies: information about BIS analysis on SF motifs and extended SF motifs.**

BIS analysis of 11 helicase subfamilies (first column). BIS was run on blocks of $d \leq 1$. Top: we report the number of co-evolving positions that belong to the SF motifs as defined in (Fairman-Williams et al. 2010) (second column), the total number of coevolving positions (third column), the ratio of the second and third columns (fourth column). Whenever a residue belongs to one of the clusters in either $d = 0$ or $d = 1$, it will be counted once. Notice that a ratio equal 1 corresponds to co-evolving residues that belong to known SF-motifs. Bottom: as on top but considering an extension of SF motifs instead of SF motifs. As defined in the article, SF motifs are extended on their right and left hand side by 5 positions. The idea is to verify whether predicted co-evolving residues are essentially located around motifs or not.

SF families	total # res in SF motifs	MST #co-ev res in SF motifs	MST #co-ev pos	MST Ratio	ELSC #co-ev res in SF motifs	ELSC #co-ev pos	ELSC Ratio	SCA-DB #co-ev res in SF motifs	SCA-DB #co-ev pos	SCA-DB Ratio	SCA-TM #co-ev res in SF motifs	SCA-TM #co-ev pos	SCA-TM Ratio
**MST, ELSC, SCA-DB and SCA-TM analyses of SF motifs**													
Upf1	64	21	35	0.60	14	22	0.64	4	8	0.50	2	5	0.40
RecD/Pif1	55	26	63	0.41	20	87	0.23	7	12	0.58	6	10	0.60
UvrD	62	31	59	0.52	22	65	0.34	8	18	0.44	8	16	0.50
Rad3	55	31	110	0.28	10	25	0.40	5	22	0.23	1	7	0.14
DEAD-box	66	17	17	1	0	0	0	4	4	1	4	4	1.00
RecQ	65	38	81	0.47	25	35	0.71	18	47	0.38	18	47	0.38
Ski2-like	62	32	53	0.60	13	70	0.19	3	6	0.50	3	6	0.50
Rig1-Like	64	48	207	0.23	13	70	0.19	3	8	0.38	3	8	0.38
Deah-RHA	63	33	55	0.60	11	15	0.73	10	17	0.59	8	13	0.62
NS3/NPH-II	62	41	132	0.31	0	2	0	1	2	0.50	1	2	0.50
Swi2/Snf2	76	15	20	0.75	4	19	0.21	0	0	0	0	0	0.00
**MST, ELSC, SCA-DB and SCA-TM analyses of extended SF motifs**													
Upf1	129	24	35	0.69	15	22	0.68	5	8	0.63	3	5	0.60
RecD/Pif1	110	35	63	0.56	23	87	0.26	7	12	0.58	6	10	0.60
UvrD	122	39	59	0.66	24	65	0.37	13	18	0.72	11	16	0.69
Rad3	120	54	110	0.49	10	25	0.40	14	22	0.64	4	7	0.57
DEAD-box	136	17	17	1	0	0	0	4	4	1	4	4	1.00
RecQ	135	53	81	0.65	28	35	0.80	31	47	0.66	31	47	0.66
Ski2-like	132	43	53	0.81	15	70	0.21	5	6	0.83	5	6	0.83
Rig1-Like	134	86	207	0.42	15	70	0.21	5	8	0.63	5	8	0.63
Deah-RHA	133	43	55	0.78	14	15	0.93	13	17	0.76	10	13	0.77
NS3/NPH-II	125	63	132	0.48	1	2	0.50	1	2	0.50	1	2	0.50
Swi2/Snf2	146	17	20	0.85	4	19	0.21	0	0	0	0	0	0.00

Table 2: **AATPase subfamilies: information on MST, ELSC, SCA-DB and SCA-TM analyses of SF motifs and extended SF motifs.**
MST, ELSC, SCA-DB and SCA-TM analyses realized on 11 helicases subfamilies (first column). MI and CTMP did not provide any prediction. For each SF family, the total size of the SF motifs, as defined in (Fairman-Williams et al. 2010), is reported (second column). Top: for each method of coevolution analysis (MST, ELSC, SCA-DB and SCA-TM) we report the total number of coevolving positions detected by the method, the number of co-evolving positions detected by the method and belonging to the SF motif, the ratio of the two numbers. Notice that a ratio equal 1 corresponds to co-evolving residues that belong to known SF-motifs. Bottom: as on top but considering an extension of SF motifs instead of SF motifs. As defined in the article, SF motifs are extended on their right and left hand side by 5 positions. The idea is to verify whether predicted coevolving residues are essentially located around motifs or not. Compare to Table 1-Text S5.

S-Fam	Name	NAB	PNB	H/NAB	NAB/PNB	H/NAB/PNB	Conserved	Not Correlated	Not a motif
SF1	Upf1	Ib Ic					Q I II III IIIa IV Va Vb VI	Ia V	iVA
SF1	RecD/Pif1	Ia V		I Ia VI<br>II IIIa Ic Vb			Q I Ia II III V Va VI		IVA Ib
SF1	UvrD			Ia VI<br>Q V	IV IVA Va		I II III IIIa IV V VA VB VI		Ib
SF2	Rad3		III Va	Ia II	Ia Ib III IV		I II III IV Va Vb VI	Q IC IVA V	IIIa
SF2	DEAD-box	Ia Ic		Ia VI			Q I II III Va VI	Ib IV Iva V Vb	IIIa
SF2	RecQ			Q I Ia IV VI<br>I Ic V	III IV Va		Q I Ia II III V Va Vb VI	Ib Iva	IIIa IB
SF2	Ski2-like	Ic V		I V		I Ia V Va	I Ia II III V Va VI	Q IV IVa Vb	IIIa
SF2	RigI-like			Ia II Vb VI	III IV Iva<br>Ia V Va		Q I Ia Ic II III IV V Va VI	Ib Iva	Ib IIIA
SF2	DEAH-RHA	Ia IV				I Ia Ic II III V Va	Q I Ia II III V Vb VI	Iva	IIIa
SF2	NS3/NPH-II			Q Ia<br>I Ia<br>Ic II<br>IV V	III IV<br>III V Va Vb		I Ia Ib Ic II III Vb VI		IIIA IB
SF2	Swi2/Snf2					Ia Va VI	I Ia III VI	Ib Ic III IIIa IV IVa	

Table 3: **AATPase subfamilies: correlations between motifs for $d \leq 1$ in SF1 and SF2 subfamilies.** Coevolution analysis highlights that known SF motifs defined in (Fairman-Williams et al. 2010) are correlated. We say that two motifs are correlated when there is at least a pair of co-evolving residues that belongs to the motifs. Correlations are summarized in several columns identifying the function of the motifs: ATP binding and hydrolysis (H), nucleic acid binding (NAB) and coordination between polynucleotide binding and ATPase activity (PNB). The last three columns (from left to right) list: motifs forming a cluster of fully conserved residues, motifs that do not contain coevolving residues, known motifs that do not belong to the specific subfamily.
