# Supplementary material for: Protein Fragments: Functional and Structural Roles of Their Coevolution Networks
Source: PLoS One. 2012 Nov 5;7(11):e48124. doi: 10.1371/journal.pone.0048124 (PMC3489791; doi:10.1371/journal.pone.0048124)
Supplement: Text S6 — AATPase supplementary figures. (PDF) [file pone.0048124.s008.pdf]

Supporting Information Text S6: AATPase supplementary figures

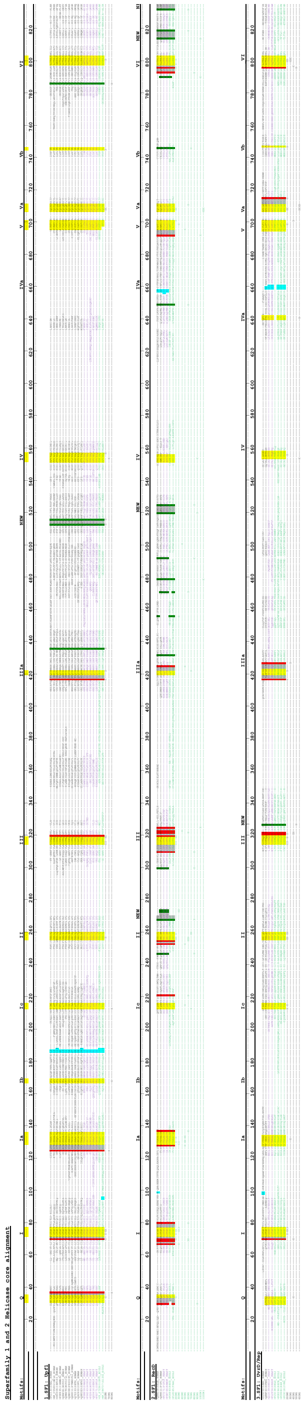

Figure 1: **AATPase: alignment and co-evolution of motifs in the SF1 family**.

Alignments and motif extensions for SF1 helicases families. Alignments are given in (Fairman-Williams et al. 2010). Each known motif is reported in yellow; red positions are co-evolved positions that are used to identify extensions of known motifs; green positions are co-evolved positions that lie distant from known motifs and that, when grouped together (with an allowed distance of ≤ 5 positions), form newly identified motifs. In grey, positions belonging to a new motif or to an extension of a known motif. Below each alignment, distinguished lines describe positions (denoted X) belonging to different co-evolving networks. Note that large insertions are removed from the alignment. These sections are marked by cyan highlights of the number of deleted residues.

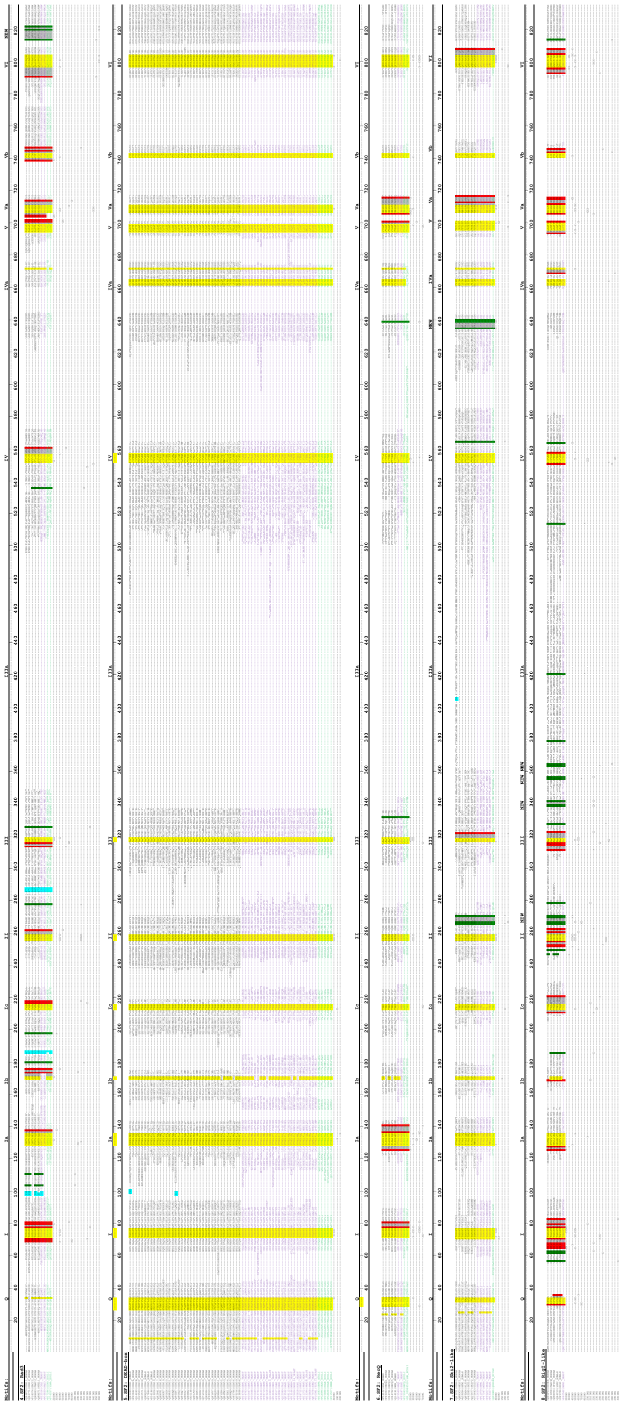

Figure 2: **AATPase: alignment and co-evolution of motifs in the SF2 family, part 1**. Alignments and motif extensions for SF2 helicases families. Legend as in Fig. 1-Text S6.

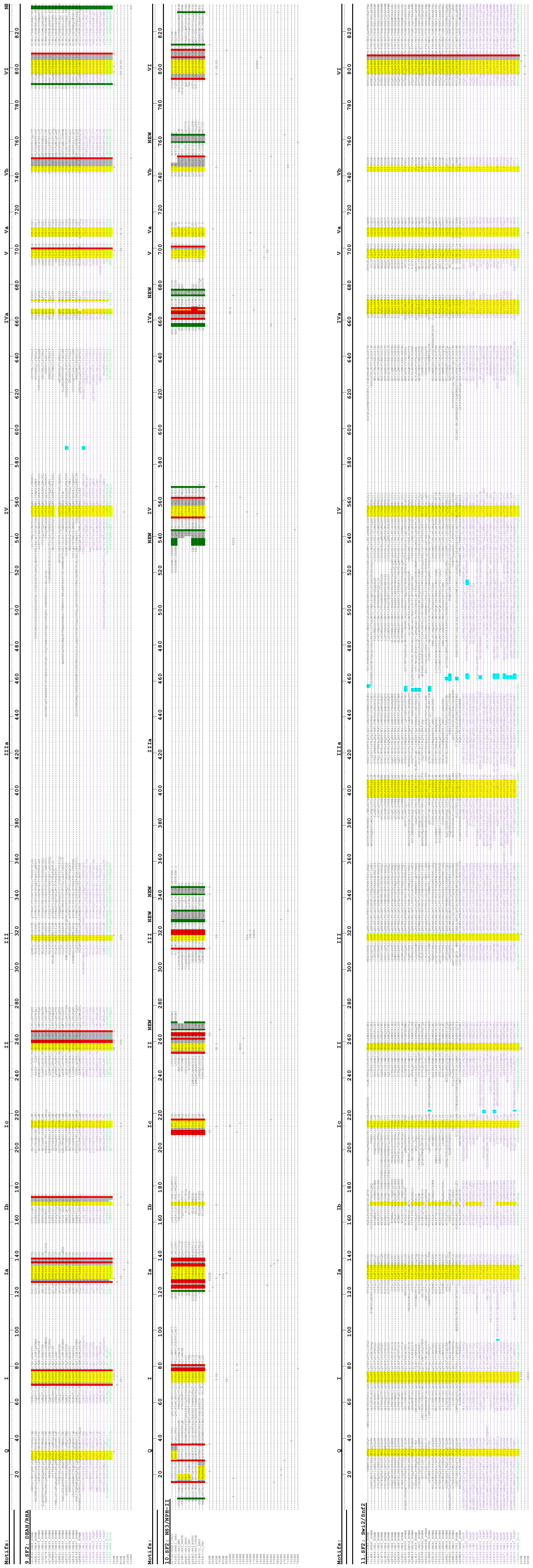

Figure 3: **AATPase: alignment and co-evolution of motifs in the SF2 family, part 2**.

Figure 2-Text S6, continued.

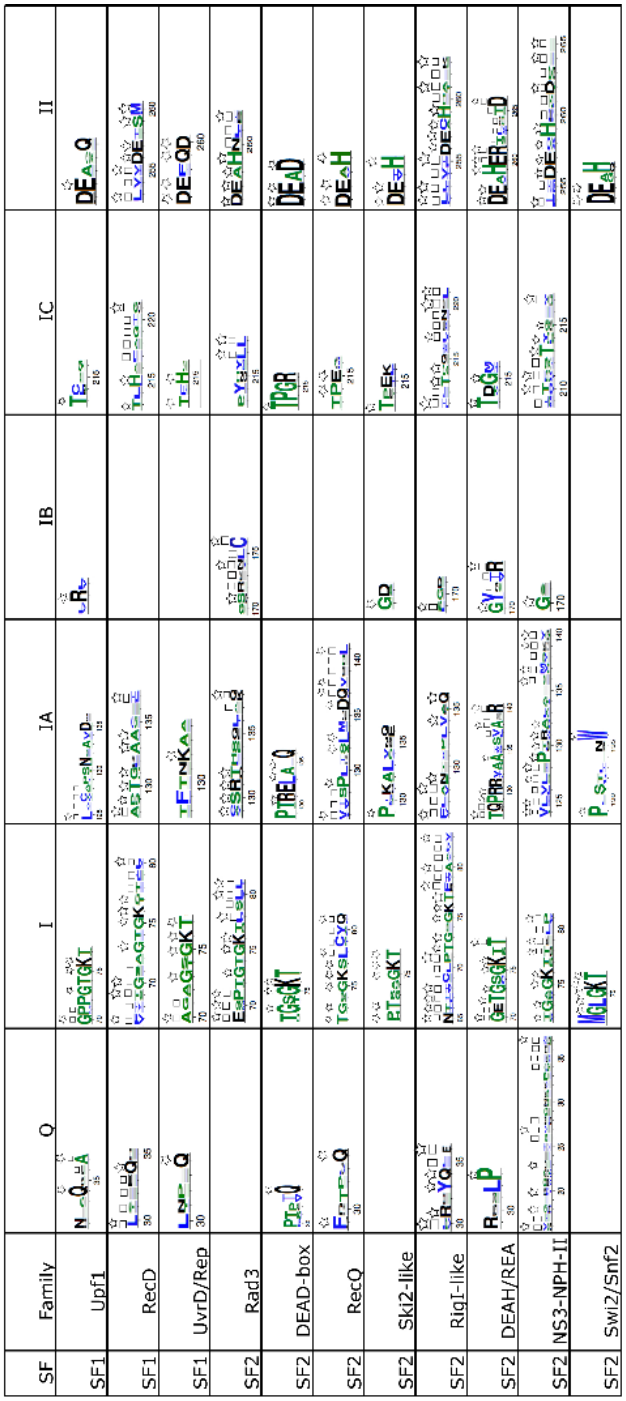

Figure 4: **AATPase: logos of protein sequence motifs, part 1**.
Extension of known motifs identified for SF1 and SF2 helicase families in (Fairman-Williams et al. 2010) and new motifs. Columns in the table correspond to motifs (the name of a known motif is reported on the top; NEW indicates that the motif is predicted by co-evolution signals) and rows correspond to distinguished families (class and family names are reported). Co-evolving residues are labelled by a star and extended residues by a square. Nine motifs are new. For motif localisation along the sequence, refer to Figs 2 and 3-Text S6. Logos are realized with the package WebLogo (Crooks GE, Hon G, Chandonia JM, Brenner SE, WebLogo: A sequence logo generator, *Genome Research*, 14:1188–1190, 2004).

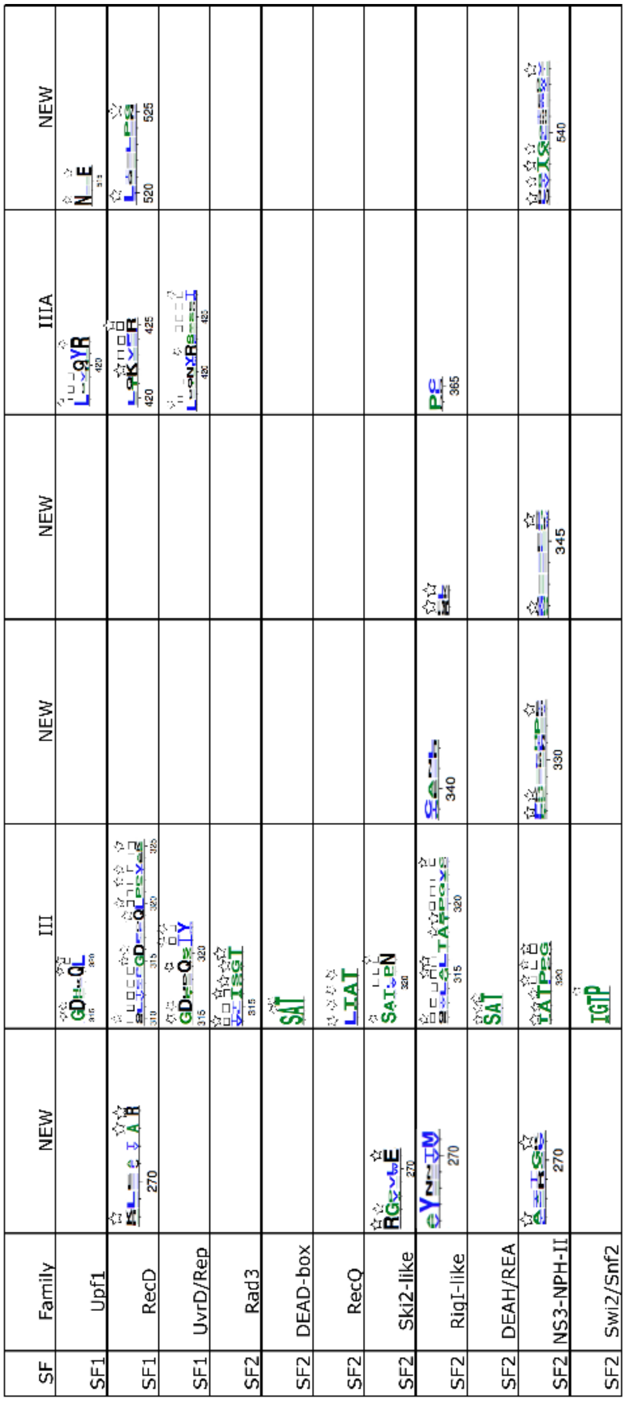

Figure 5: **AATPase: logos of protein sequence motifs, part 2**.
Figure 4-Text S6 continued.

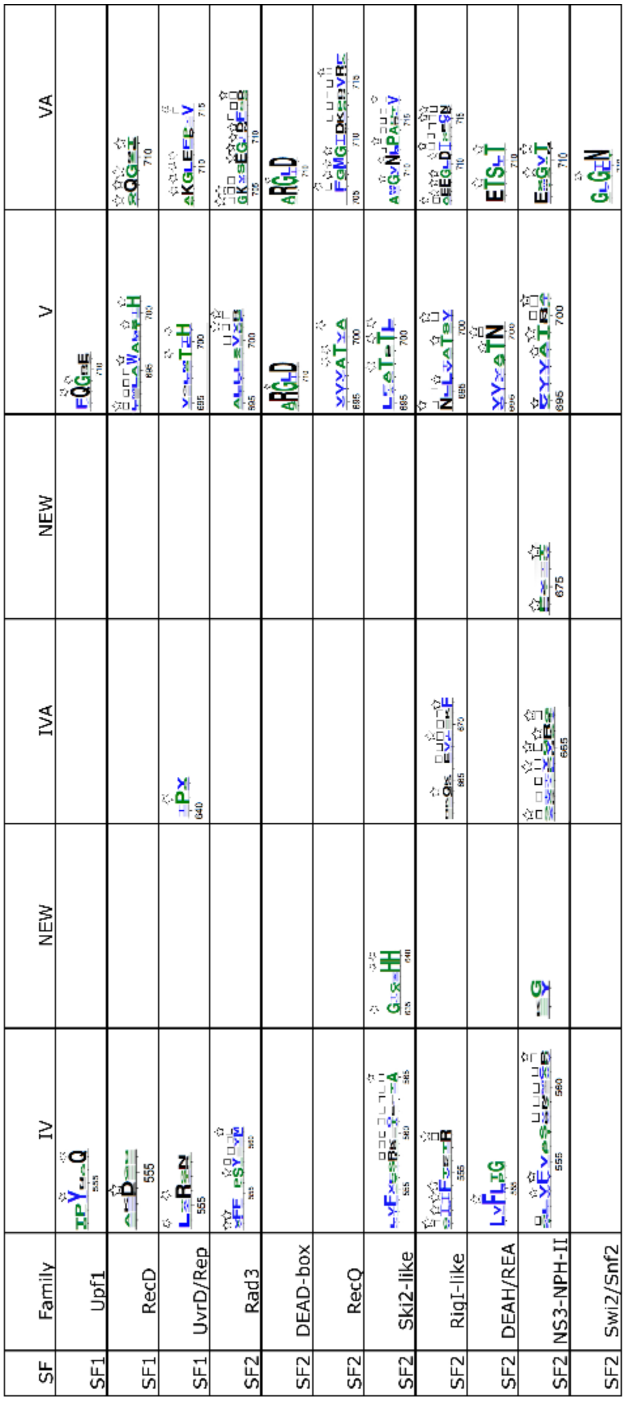

Figure 6: **AATPase: logos of protein sequence motifs, part 3**.
Figure 4-Text S6 continued.

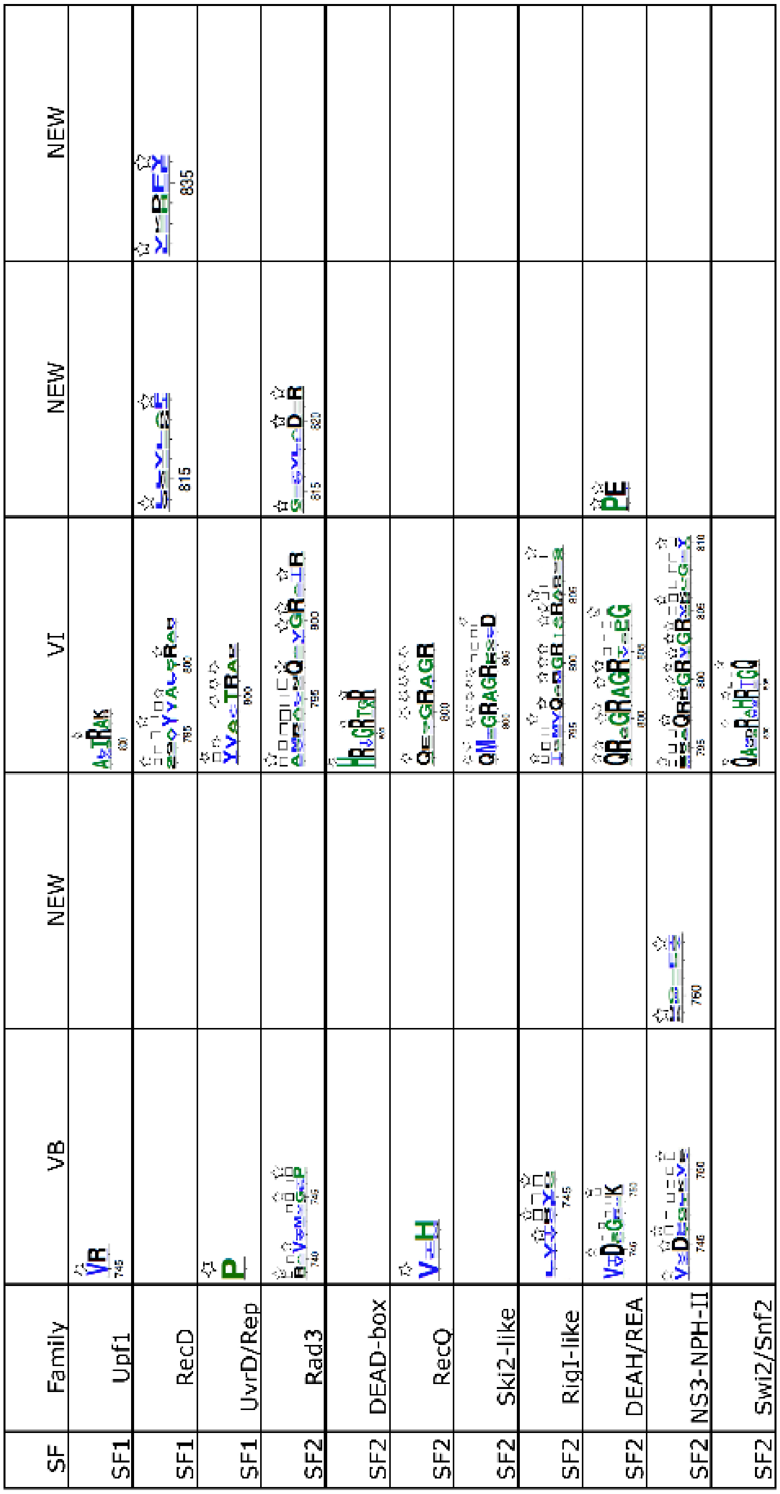

Figure 7: **AATPase: logos of protein sequence motifs, part 4**.

Figure 4-Text S6 continued.
