# Supplementary material for: Protein Fragments: Functional and Structural Roles of Their Coevolution Networks
Source: PLoS One. 2012 Nov 5;7(11):e48124. doi: 10.1371/journal.pone.0048124 (PMC3489791; doi:10.1371/journal.pone.0048124)
Supplement: Text S7 — BIS coevolution analysis based on physico-chemical properties. (PDF) [file pone.0048124.s009.pdf]

Supporting Information Text 7: BIS coevolution analysis based on physico-chemical properties

Protein A-B domain			
Method	Cl	Positions	Sym
BIS, *block*, $d = 0$	C1	20 22-24 27 29	1
	C2	29-40 42 45-46 48-49	1
	C3	29-42 44-46	1
	C4	51 52	1
BIS, *block*, $d = 1$	C5	11-15 17-18	1
	C6	53 54	1

Table 1: **Coevolution analysis based on physico-chemical properties: protein A-B domain.** BIS analysis based on physico-chemical properties was run on the protein A-B domain family of 452 sequences.

MukB protein			
Method	Cl	Positions	Sym
BIS, *block*, $d = 0$	C1	4 6 9	1
	C2	13-20 22-26	1
	C3	34-37 39-42	1
	C4	10 11	1
BIS, *block*, $d = 1$	C5	44-45 47-48 51-54 56-58 60-67 69 72-81 84 86-87 90-91 94-95 99 101 104 106 108-109 112-113 115-116 118 121 123 126 135-136 140 146-147 150 152-154 157 165 170-171 173 175 178-180 182-183 186-189 193-194 196 198-211 213-223 225 227	1
	C6	98-99 173-175	1
	C7	56-67 101-102 132-133	1
	C8	104-109 111-119 165-166 182-184 186-190	1
	C9	51-58 135-137	1
	C10	47-49 145-147	1
	C11	28 29 30 31 32 33	1

Table 2: **Coevolution analysis based on physico-chemical properties: MukB protein.** BIS analysis based on physico-chemical properties was run on the MukB family of 200 sequences.

Amyloid beta peptide			
BIS, *block*, $d = 0$	C1	11-12 16-20	1
	C2	18-20 23-24 26-38	1
BIS, *block*, $d = 1$	C3	21 22	1
	C4	39 40 41 42	1

Table 3: **Coevolution analysis based on physico-chemical properties: Amyloid beta peptide.** BIS analysis based on physico-chemical properties was run on the Amyloid family of 80 sequences.

AATPase Upf1			
BIS, *block*, $d = 0$	C1	34 37-38 70 73 75-77 83-84 88 125 130 134 168-169 210 213 254-257 267 312-316 319-320 362-363 417 422 427 436 513 516 551 557 561 634 700 708-709 715 741-743 745 788 795 799-801 806 809 820 833	1
BIS, *block*, $d = 1$	C2	124-125 440	1
	C3	137 141 797	1
	C4	209-210 270 361-363 554	1
	C5	432 516-517	1
	C6	67-68 548 550-551 697 786	1

Table 4: **Coevolution analysis based on physico-chemical properties: AATPase Upf1.** BIS analysis based on physico-chemical properties was run on the Upf1 family of 18 sequences.

AATPase Ski2-like			
BIS, *block*, $d = 0$	C1	71-72 127	1
	C2	67 72 75-77 129 133 141 211 250 253-257 259 261 314 316-317 320 326 329-330 632 635-636 639-640 643 665 692 694 698 709-712 717 741-742 797-798 800-804 808	1
	C3	84 209	1
	C4	700 701	1
BIS, *block*, $d = 1$	C5	67-68 131 145 168 672	1
	C6	66-67 170 671	1
	C7	80 213 252-257 266 271 552-553 558 565 568 818	1
	C8	253-259 267 270 322 554 694-695 708-713 819	1

Table 5: **Coevolution analysis based on physico-chemical properties: AATPase Ski2-like.** BIS analysis based on physico-chemical properties was run on the Ski2-like family of 13 sequences.

AATPase RecD			
Method	Cl	Positions	Sym
BIS, *block*, $d = 0$	C1	79-80 127 247 440	1
	C2	34 37 40-41 67 69-70 73 75-77 80 83-84 87 130 133-134 137 253-257 259-261 266 269 311-313 315-316 319-320 323 421-422 432 435 496-497 520 528 554 699-701 708-709 746 795-796 798-801 819 832 835	1
	C3	65-67 97 129-130 132-134 272-274 299 310-313 417 454 456-458 462 465 476 520-521 551 553-554 806 808 811 819-820 835-836 839	1
	C4	133-135 852 855	1
	C5	239 813	1
	C6	249 845	1
	C7	311-316 323-324 641	1
	C8	439 501 552 712 790 814 817	1
	C9	484 745-746	1
	C10	668 818-819	1
	C11	67-70 803	1
	C12	708-710 812	1
	C13	711 795-801	1
BIS, *block*, $d = 1$	C14	90 94 124 252-257	1
	C15	30 125 421-423 643 692 824	1
	C16	126 128 130-131 214-215 265-266 334 420-422 425 646-647 696 740	1
	C17	213 268-269 442 479 512 523 530 623 645 698-701 798-802	1
	C18	218 832-833	1
	C19	221 269-270 322-323 471 646-649	1
	C20	238 480	1
	C21	300 305 318-320 492 525 633 685 687 793	1
	C22	302 325 417-418 426 435-436 561 650	1
	C23	336 823	1
	C24	36-37 510 697	1
	C25	406 672	1
	C26	482 504	1
	C27	483 506	1
	C28	499 670	1
	C29	75-78 92 502 681	1
	C30	240 241	1

Table 6: **Coevolution analysis based on physico-chemical properties: AATPase RecD.** BIS analysis based on physico-chemical properties was run on the RecD family of 6 sequences.

**AATPase UvrD**			
Method	Cl	Positions	Sym
BIS, *block*, $d = 0$	C1	68 73-77 125 129 213 215 253 256-257 259-260 268-269 313 315-316 319 321 417 420 422 427 434-435 463 553-554 556 697 699-701 708-709 715 717 747 791 795 800-801 825	1
	C2	125-126 133	1
	C3	128-129 635	1
	C4	81 312-313	1
	C5	745 793	1
	C6	37 38	1
BIS, *block*, $d = 1$	C7	34 70 80 124-125 141 209 213-215 217 222 253-257 313-316 321-322 325 746-747 794-795 797	1
	C8	68-69 132 712 800-802 806	1
	C9	142 665-666	1
	C10	88 145 311 695	1
	C11	237 427-428 641 651 708-711	1
	C12	250 328	1
	C13	327 795-796 799-801	1
	C14	94 340	1
	C15	450 485 742	1
	C16	496 697-701	1
	C17	558 679	1
	C18	629 635-636	1
	C19	631 634-635	1
	C20	632 644	1
	C21	674 720	1
	C22	839 842	1
	C23	239 240	1

Table 7: **Coevolution analysis based on physico-chemical properties: AATPase UvrD.** BIS analysis based on physico-chemical properties was run on the UvrD family of 8 sequences.

AATPase Rad3			
Method	Cl	Positions	Sym
BIS, *block*, $d = 0$	C1	105 329	1
	C2	111 115	1
	C3	80-81 126 130-132 143 171 318-320 330 633 700 704-710 739 745-746	1
	C4	128 174	1
	C5	34 67-69 72-73 75-77 81 85 130-131 136 139 176 218 252-257 259 261 273 306 313-315 319-320 538 551 553 557 561 568 697 708-710 712 741 746 756 797 799 801	1
	C6	67-70 133 138-139 198	1
	C7	139-140 640	1
	C8	213 714	1
	C9	261-262 702 704-712	1
	C10	269 291 313-317 708-712 814	1
	C11	87 326	1
	C12	564 748	1
BIS, *block*, $d = 1$	C13	102 106 335	1
	C14	104 107 111-112	1
	C15	113 761	1
	C16	117 282 305-306	1
	C17	125-126 143-144	1
	C18	13 846	1
	C19	134 165	1
	C20	71-73 75-78 169 210 217-218 323 549-551 553-554	1
	C21	175-176 639 743	1
	C22	38 72-77 176-177 201 209 252-261 273-274 278 557-558 697-698 741-742 746-747 759 808	1
	C23	199 565	1
	C24	204 297 819	1
	C25	211 542 695	1
	C26	223 845	1
	C27	23 31	1
	C28	85-86 249	1
	C29	298 300	1
	C30	92 326-327	1
	C31	520 629-630	1
	C32	540 661	1
	C33	571 740-741	1
	C34	669 696-697	1
	C35	795 799-801 803-804 816 818 820 822	1

Table 8: **Coevolution analysis based on physico-chemical properties: AATPase Rad3.** BIS analysis based on physico-chemical properties was run on the Rad3 family of 9 sequences.

AATPase DEAD-box			
Method	Cl	Positions	Sym
BIS, *block*, $d = 0$	C1	33-34 38 72-73 75-77 85 127 133 209 213 217 254-257 259 315 318-319 552 672 699 708-710 715 741 797 804	1
BIS, *block*, $d = 1$	C2	130 696	1
	C3	136 801	1
	C4	317-319 712	1

Table 9: **Coevolution analysis based on physico-chemical properties: AATPase DEAD-box.** BIS analysis based on physico-chemical properties was run on the Ski2-like family of 67 sequences.

AATPase RecQ			
Method	Cl	Positions	Sym
BIS, *block*, $d = 0$	C1	24 34 67-68 72 75-78 125 127 129 133 136 141 210 217 253 255-259 314 317-320 552-553 568 639 696 699 706 708-710 715 797 801-804	1
	C2	75-79 125-127 255-260 327 332 552-554 668 699-700 706-710 712 715-716 795	1
	C3	160 338-339	1
	C4	518 564	1
	C5	80 695-696	1
BIS, *block*, $d = 1$	C6	124-127 263	1
	C7	42 70 72-73 129-130 209-210 214-215 268 316-320 328 698-699	1
	C8	29 81 133-134 136-137 212 264 555 665 672 746 792 800-804	1
	C9	207 792-793	1
	C10	213 250	1
	C11	244 815	1
	C12	65 267	1
	C13	82 84 562 717	1
	C14	669 744	1
	C15	85 693	1

Table 10: **Coevolution analysis based on physico-chemical properties: AATPase RecQ.** BIS analysis based on physico-chemical properties was run on the RecQ family of 9 sequences.

AATPase RigI-like			
Method	Cl	Positions	Sym
BIS, *block*, $d = 0$	C1	31 33-34 57 65 67 70 72-73 75-77 79 83 125-126 128 136 213 217 250 253-257 259 267 270-271 317-319 379 383 552-554 558 561 564 567-568 581 669 672 694 696-697 699 701 707-712 741-743 796-797 800-801 804 815-817	1
	C2	128-129 131 252-259 343 706-712 799-801 804-805 808	1
	C3	65-67 75-79 130 746	1
	C4	139 522 854	1
	C5	145 169 275 321	1
	C6	36 176 261 279 312 315 334 341	1
	C7	90 219-220	1
	C8	245 247-248 264 803-804	1
	C9	30-31 266-267 400 552-555 661	1
	C10	322 333	1
	C11	345 356 545 796-801	1
	C12	421 540 641	1
	C13	452 501 512	1
	C14	57-58 453	1
	C15	87 745	1
	C16	715 716	1
BIS, *block*, $d = 1$	C17	70-73 124-126 133 168 181 210-211 216-217 221 313-314 316-319 331 364 387 538 793	1
	C18	33-35 60-61 67-68 81 125-128 335	1
	C19	79-80 134 336 449 560-561 815-818	1
	C20	141 146	1
	C21	143 439 458	1
	C22	23 147 392	1
	C23	148-149 365 551-554 671-672 741-744 747 814-818	1
	C24	166 575 829	1
	C25	171 509 520 845	1
	C26	63 186 209-211 263 274 278-279 323 339-343 349 357 386-387 557-558 748	1
	C27	20 827	1
	C28	213-214 223 548 806	1
	C29	217-218 462	1
	C30	224 503 549 813	1
	C31	62 225 324 418	1
	C32	262 430	1
	C33	28 325 412 556 571 636	1
	C34	311-312 511 842	1
	C35	328 361 383-384 433 672-673	1
	C36	329 640	1
	C37	347 644 664 668-669 694-697 699-701	1
	C38	351 665	1
	C39	9 353	1
	C40	359 416 497	1
	C41	381 856	1
	C42	385 836	1
	C43	401 500 794	1
	C44	406-407 826 840	1
	C45	411 850	1
	C46	72-77 434 564-565 570	1
	C47	531 844	1
	C48	534 832	1
	C49	547 662 749	1
	C50	715 716	1

Table 11: **Coevolution analysis based on physico-chemical properties: AATPase RigI-like.** BIS analysis based on physico-chemical properties was run on the RigI-like family of 6 sequences.

AATPase DEAH-RHA			
Method	Cl	Positions	Sym
BIS, *block*, $d = 0$	C1	32-33 41 67-68 73 75-78 80 124 129 132 137 140-141	
		173 209 217 253-258 260-261 266 268 272 313 315 318-319	
		696-697 699 701 710-712 744-745 791 795 798 801 808 817	
		835	1
	C2	265-266 833	1
BIS, *block*, $d = 1$	C3	70 127 840	1
	C4	72-73 129-132 173-174 213 215 253-261 266-269 311 313-315	
		317-319 325 699-701 708 750 797-798 800-801 803-804	1
	C5	134 551-552 554-555 561	1
	C6	137-138 170	1
	C7	216-217 701-702 707	1
	C8	247 693	1
	C9	271-272 847	1
	C10	743-745 760	1
	C11	833-835 837	1

Table 12: **Coevolution analysis based on physico-chemical properties: AATPase DEAH-RHA.** BIS analysis based on physico-chemical properties was run on the DEAH-RHA family of 24 sequences.

AATPase NS3-NPH-II			
Method	Cl	Positions	Sym
BIS, *block*, $d = 0$	C1	68 73 75-77 125-126 129 141 170 209 213 253-254 256-257 259 267 314 316-319 552-553 557 568 696-697 699 711 740 743 745 797-798 800-801 803-804	1
	C2	75-78 124-126 708	1
	C3	80 125-127 137 266-267 316-320 322 695-697 701 709	1
	C4	8-9 13 35 79-80 536-539 674	1
	C5	129-130 316-322 834	1
	C6	131 212-213	1
	C7	132 314-319 552-554 797-801	1
	C8	140-141 665	1
	C9	17-18 211 538	1
	C10	209-210 806	1
	C11	72-73 213-214 664 810	1
	C12	215 562 856	1
	C13	217 751	1
	C14	218 262 666	1
	C15	39 219 566 794	1
	C16	22 256-259 264	1
	C17	84 253-257 267-268 711-712 800-804	1
	C18	26 37	1
	C19	9 265-267 792 841	1
	C20	30 327	1
	C21	312 539 559 661 759	1
	C22	338 543	1
	C23	342 667	1
	C24	346 659 847	1
	C25	347 540 747	1
	C26	55 540-541	1
	C27	544 822	1
	C28	551-553 677	1
	C29	70 658	1
	C30	759-761 838	1
	C31	795 813	1
	C32	845 850	1
BIS, *block*, $d = 1$	C33	121 259-260 832	1
	C34	133 710-711 754	1
	C35	136-137 844	1
	C36	14 334	1
	C37	15 821-822	1
	C38	20 23 324	1
	C39	250 313-314	1
	C40	271 811	1
	C41	28 311	1
	C42	51-52 325	1
	C43	38 693	1
	C44	49 750	1
	C45	757 848	1
	C46	829 849	1
	C47	790 791	1

Table 13: **Coevolution analysis based on physico-chemical properties: AATPase NS3-NPH-II.** BIS analysis based on physico-chemical properties was run on the NS3-NPH-II family of 11 sequences.

AATPase Swi2-Snf2			
Method	Cl	Positions	Sym
BIS, *block*, $d = 0$	C1	67-68 72-77 126 136 140 256-257 261 314 316 319-320 334 695-697 711-712 745 747 804 814	1
BIS, *block*, $d = 1$	C2	129 316-317 797 801 807	1
	C3	70-77 217 395	1
	C4	319-321 396 710-712	1
	C5	332 792 827	1
	C6	84 791	1

Table 14: **Coevolution analysis based on physico-chemical properties: AATPase Swi2-Snf2.**
BIS analysis based on physico-chemical properties was run on the Swi2-Snf2 family of 45 sequences.

BIS vs BIS$_{pc}$ analysis of AATPase SF motifs						
	BIS$_{pc}$			BIS		
SF family	# residues in SF motifs	# co-evolving positions	Ratio	# residues in SF motifs	# co-evolving positions	Ratio
UPF1	26	76	0.34	18	26	0.69
RECD	45	198	0.23	32	73	0.44
UvrD/rep	38	117	0.32	30	38	0.79
Rad3	43	169	0.25	28	62	0.45
DEAD-box	27	38	0.71	16	16	1
RecQ	42	104	0.40	31	42	0.74
Ski2-like	34	83	0.41	22	35	0.63
RigI-like	56	266	0.21	40	104	0.38
DEAH-RHA	43	95	0.45	29	43	0.67
NS3-NPH-II	45	172	0.26	36	101	0.36
Swi2-Snf2	22	45	0.49	14	15	0.93
**Extended SF motifs**						
UPF1	35	76	0.46	19	26	0.73
RECD	67	198	0.34	64	197	0.32
UvrD/rep	52	117	0.44	46	76	0.61
Rad3	71	169	0.42	63	157	0.40
DEAD-box	32	38	0.84	16	16	1
RecQ	63	104	0.61	56	92	0.61
Ski2-like	44	83	0.53	26	37	0.70
RigI-like	96	266	0.36	88	259	0.34
DEAH-RHA	60	95	0.63	41	48	0.85
NS3-NPH-II	73	172	0.42	68	152	0.45
Swi2-Snf2	30	45	0.67	17	17	1

Table 15: **BIS vs BIS based on physico-chemical properties in AATPase motifs analysis**
Analysis of BIS and BIS based on physico-chemical properties (BIS$_{pc}$) of the 11 helicases subfamilies. BIS and BIS$_{pc}$ were run on blocks of $d \leq 1$. Top: we report the number of coevolving motifs that belong to the SF motifs as defined in (Fairman-Williams et al. 2010) (second and fifth columns), the total number of coevolving positions (third and sixth columns), the ratio of the second and third/fifth and sixth columns (fourth and eigth columns). Whenever the residue belongs to one of the clusters in either $d = 0$ or $d = 1$, it will be counted once. Notice that a ratio equal 1 corresponds to co-evolving residues that belong to known SF-motifs. Bottom: as on top but considering an extension of SF motifs instead of SF motifs. As defined in the article, SF motifs are extended on their right and left hand side by 5 positions. The idea is to verify whether predicted coevolving residues are essentially located around motifs or not.

BIS analysis of co-evolution based on physico-chemical properties

	Size	API	Pos	#Exp	#CoRes	TP	FN	FP	TN	Prob	Sen*	Spe*	Acc*	PPV*
Amyloid	80	0.90	43	25	28	22	3	6	12	$2.99e^{-4}$	0.88	0.67	0.79	0.79
MukB: Walker-A	200	0.89	234	8	8	7	1	1	255	$9.16e^{-12}$	0.88	1	0.99	0.88
Protein A-B domain	452	0.86	57	36	35	29	7	6	15	$1.42e^{-4}$	0.81	0.71	0.77	0.83
Upf1	18	0.68	677	64	76	26	38	50	563	$7.68e^{-11}$	0.41	0.92	0.87	0.34
Ski2-like	13	0.61	686	62	83	34	28	49	575	$2.19e^{-18}$	0.55	0.92	0.89	0.41
RecD	6	0.58	642	55	198	45	10	153	434	$3.50e^{-16}$	0.82	0.74	0.75	0.23
UvrD/Rep	8	0.60	661	62	117	38	24	79	520	$3.44e^{-16}$	0.61	0.87	0.84	0.32
Rad3	9	0.62	592	55	169	43	12	126	411	$1.06e^{-15}$	0.78	0.77	0.77	0.25
DEAD-box	67	0.69	624	66	38	27	39	11	547	$9.35e^{-21}$	0.41	0.98	0.92	0.71
RecQ	9	0.68	514	65	104	42	23	62	387	$2.25e^{-17}$	0.65	0.86	0.83	0.40
RigI-like	6	0.53	656	64	266	56	8	210	382	$3.62e^{-16}$	0.88	0.65	0.67	0.21
DEAH-RHA	24	0.73	562	63	95	43	20	52	447	$6.35e^{-23}$	0.68	0.90	0.87	0.45
NS3-NPH-II	11	0.66	479	62	172	45	17	127	290	$3.10e^{-10}$	0.73	0.70	0.70	0.26
Swi2-Snf2	45	0.62	714	76	45	22	54	23	615	$1.26e^{-11}$	0.29	0.96	0.89	0.49

Table 16: **Validation of performance of coevolution analysis on physico-chemical properties.** Percentage of identity computed on residue physico-chemical properties (API), number of sequences (Seq), alignment length (where all gapped positions, if any, are eliminated; Pos), number of experimentally confirmed residues (#Exp), number of residues identified by coevolution analysis (BIS), number of true positives (TP) computed by intersecting #Exp and BIS, probability of predicting TP residues out of #Exp by selecting BIS residues within Len residues (Prob), Sensitivity (Sen), Specificity (Spe), Accuracy (Acc), Positive Predictive Value (PPV) are given.). For Amyloid, experimentally validated residues (#Exp) have been obtained from the sources cited in the text; for Protein A we considered hotspots together with the large peptide fragment 25-59 and residues 14, 17 cited in (Sato et al. 2006) to have high ϕ-value; MukB analysis is evaluated on Walker-A detection; Upf1 and Ski2-like are evaluated on known motifs described in (Fairman-Williams et al. 2010). The number of coevolving residues detected by the methods are reported in column #CoRes. Compare with Table 2 in the article. Sen, Spe, Acc, PPV have been evaluated with respect to experimentally validated residues; they are marked with * to remind this. The same is true for Prob.
