# Supplementary material for: Protein Fragments: Functional and Structural Roles of Their Coevolution Networks
Source: PLoS One. 2012 Nov 5;7(11):e48124. doi: 10.1371/journal.pone.0048124 (PMC3489791; doi:10.1371/journal.pone.0048124)
Supplement: Text S8 — Comparison of coevolution analysis methods on aligned sets of sequences representing AATPase families. (PDF) [file pone.0048124.s010.pdf]

Supporting Information Text S8: comparison of coevolution analysis methods on aligned sets of sequences representing AATPase families

**AATPase Upf1 comparing methodologies**			
Method	Cl	Position Alignment	Sym
BIS, *pos*, $d = 0$	C1	34 70 73 75 76 257 315 316 319 320 417 422 436 513 516 557 708 709 745 801	1
BIS, *pos*, $d = 1$	C1	125 554	1
	C2	37 168 213 786	1
BIS, *blocks*, $d = 0$	C1	34 70 73 75-76 257 315-316 319-320 417 422 436 513 516 557 708-709 745 801	1
BIS, *blocks*, $d = 1$	C3	37 168 213 786	1
	C4	125 554	1
ELSC	C1	**34 70 73 75 76** 186 187 **257 315 316 319**	0
		**320 417 422 436 513 516 557 708 709 745 801**	
	C1	256 796	1
SCA-DB	C2	**125 554**	0.9
	C3	**37 168 213 786**	0.9
SCA-TM	C1	256 796	1
	C2	**37 213 786**	0.9
MI	NO MATRIX		
	C1	**34 37 70 73 75 76 168** 186 187 **213 257 315 316 319 320 417 422 436**	1
		**513 516 557 708 709 745 786 801**	1
	C2	**125 554** 746	0.81
MST	C3	135 440	0.71
	C4	131 263	0.71
	C5	743 798	0.71
CTMP	NO MATRIX		

Table 1: **AATPase Upf1: comparing methodologies.**
For each method, we report the clusters of co-evolving residues detected by the clustering algorithm CLAG (with environmental score equal 1, symmetric score > 0 and $\Delta = 0.05$). For BIS we selected clusters with symmetric score $= 1$ only. For each methodology we report the number of sequences (Al), the names of the clusters (Cl), the positions on the PDB structure (Positions). Results are reported for BIS run on blocks and on single alignment positions. SCA, ELSC, MI, MST and CTMP methods are compared to BIS execution when considering alignment positions (*pos*). Common residues predicted by both methodologies are highlighted in bold. Green residues are those belonging to the Walker-A. NO MATRIX means that the method did not output any co-evolution pair; NO CLUSTERS means that the method output co-evolution pairs that could not be clustered together.

AATPase RecD comparing methodologies			
Method	Cl	Positions	Sym
BIS, *pos*, $d = 0$	C1	67 77 129 273 274 456 699	1
	C2	34 70 73 75 76 130 133 256 257 259 315 316 319 320 432 701 708 709 796 798 801	1
	C3	323 501	1
	C4	797 799 800	1
BIS, *pos*, $d = 1$	C1	128 131 134 215 253 425 696	1
	C2	69 80 137 221 255 322 471 649 711 819 832	1
	C3	213 260 422 746	1
	C4	247 268 300 310 492 525 793 836	1
	C5	30 520 554	1
	C6	325 692 790 814 839	1
	C7	479 707	1
BIS *blocks*, $d = 0$	C1	34 70 73 75-76 130 133 256-257 259 315-316 319-320 432	1
		701 708-709 796 798 801	1
	C2	67 75-77 129-130 273-274 456 699	1
	C3	323 501	1
	C4	797 799 800	1
BIS, *blocks*, $d = 1$	C1	128 130-131 133-134 215 253 425 696	1
	C2	69-70 80 137 221 255-257 322 471 649 711 819 832	1
	C3	213 259-260 422 746	1
	C4	47 268 300 310 492 525 793 836	1
	C5	30 520 554	1
	C6	325 692 790 814 839	1
	C7	479 707-709	1
ELSC	C1	**797 799 800**	1
	C2	**215 253**	1
	C3	**131 134**	1
	C4	643 644 645 646	0.9
	C5	210 211	0.9
	C6	258 317	0.9
	C7	639 640	0.9
	C8	684 686	0.9
	C9	454 455	0.7
	C10	460 465	0.7
	C11	458 462 464	0.7
	C12	217 218	0.7
	C13	453 457	0.2
	C14	748 749 750 751 752	0.2
	C15	445 459	0.2
	C16	568 569 572 573	0.2
	C17	105 106 107 108 109 110 116 117 118 119 120 121 122 123	0.2
	C18	429 430	0.2
	C19	444 452	0.2
	C20	461 463	0.2
	C21	567 574	0.2
	C22	861 862	0.2
	C23	850 851	0.2
	C24	**130 133 256 257 259 315 316 319 320 432 701 708 709 796 798 801** 860 863	0.2
	C25	570 571	0.2
SCA-DB	C1	**128 131 134 215 253 696**	1
	C2	**213 260**	1
	C3	475 697	0.9
	C4	31 212	0.5
SCA-TM	C1	**128 131 134 215 253 696**	1
	C2	**213 260**	1
	C3	31 212	0.5
MI	NO MATRIX		

Table 2: **AATPase RecD: comparing methodologies. Part 1.**

See legend of Table 1-Text S8. Continued in Table 3-Text S8.

AATPase RecD comparing methodologies			
Method	Cl	Positions	Sym
MST	C1	**34 70 73 75 76 128 130 131 133 134 215 253 256 257 259 315 316 319 320 425 432 696 701 708 709 796 798 801**	1
	C2	528 740	1
	C3	36 212	0.65
	C4	266 745 834	0.65
	C5	313 680	0.65
	C6	214 697	0.65
	C7	254 312 475	0.65
	C8	**30** 127 311 321 334 **520 554** 657 658 700 835	0.65
	C9	515 695	0.39
	C10	326 514	0.39
	C11	261 829	0.39
	C12	38 82 216 857	0.04
CTMP	NO MATRIX		

Table 3: **AATPase RecD: comparing methodologies. Part 2.**

Table 2-Text S8 continued.

AATPase UvrD/Rep comparing methodologies			
Method	Cl	Positions	Sym
BIS, *pos*, $d = 0$	C1	73 75 76 77 213 215 256 257 259 260 315 316 319 321 417 422 556 699 708 709 715 747 800 801	1
BIS, *pos*, $d = 1$	C1	132 427 802	1
	C2	34 70 322 701 797	1
	C3	327 796	1
	C4	554 641 710 711	1
BIS, *blocks*, $d = 0$	C1	73 75-77 213 215 256-257 259-260 315-316 319 321 417 422 556 699 708-709 715 747 800-801	1
BIS, *blocks*, $d = 1$	C1	132 427 800-802	1
	C2	34 70 321-322 701 797	1
	C3	327 796	1
	C4	554 641 708-711	1
ELSC	C1	742 743	0.9
	C2	**710 711**	0.9
	C3	628 629 630	0.7
	C4	631 633	0.7
	C5	488 490 491	0.7
	C6	838 839	0.7
	C7	683 684 685	0.7
	C8	680 682	0.7
	C9	205 206	0.3
	C10	664 667 668	0.3
	C11	**73 75 76 77 213 215** 239 240 **256 257 259 260 315 316 319 321 417 422 556** 659 660 661 **699 708 709 715 747 800 801** 833	0.3
	C12	671 675	0.3
	C13	663 666	0.3
	C14	21 27	0.3
	C15	19 22 25	0.3
	C16	672 673	0.3
SCA-DB	C1	420 423	1
	C2	**70 322 701 797**	1
	C3	**554 710 711**	1
	C4	140 328	1
	C5	**327 796**	1
	C6	**132 427 802**	1
	C7	141 799	0.6
SCA-TM	C1	**70 322 701 797**	1
	C2	**710 711**	1
	C3	**554 710 711**	1
	C4	**554 711**	1
	C5	**327 796**	1
	C6	**132 427 802**	1
	C7	**554 710**	0.9
	C8	434 697	0.7
	C9	141 799	0.7
MI	NO MATRIX		
MST	C1	**73 75 76 77 213 215** 239 240 **256 257 259 260 315 316 319 321 417 422 556 699 708 709 715 747 800 801**	1
	C2	39 43 44 808	1
	C3	**327 796**	0.71
	C4	**554 641 710 711**	0.71
	C5	**132** 139 **427 802**	0.71
	C6	**34 70 322 701 797**	0.71
	C7	552 806	0.62
	C8	211 312	0.62
	C9	557 745 826	0.62
	C10	659 660	0.62
	C11	91 330 421 426 466	0.33
CTMP	NO MATRIX		

Table 4: **AATPase UvrD/Rep: comparing methodologies.**

See legend of Table 1-Text S8.

AATPase Rad3 comparing methodologies			
Method	Cl	Positions	Sym
BIS, pos, $d = 0$	C1	80 132 171 318 553 704 705 739 745	1
	C2	138 198	1
	C3	69 73 75 76 176 256 257 259 319 557 708 709 741 797 801	1
	C4	262 701	1
	C5	314 561	1
	C6	81 316 317 711	1
	C7	70 714	1
BIS, pos, $d = 1$	C1	104 111	1
	C2	129 174	1
	C3	131 218 258 278 747	1
	C4	180 536	1
	C5	71 217 549	1
	C6	78 326	1
	C7	551 710	1
	C8	791 814	1
	C9	800 804 820 822	1
BIS, $blocks$, $d = 0$	C1	80 132 171 318-319 553 704-705 739 745	1
	C2	138 198	1
	C3	69 73 75-76 176 256-257 259 319 557 708-709 741 797 801	1
	C4	262 701-702	1
	C5	314 561	1
	C6	81 316-317 711	1
	C7	69-70 714	1
BIS, $blocks$, $d = 1$	C1	104 111	1
	C2	129-130 174	1
	C3	131 218 256-259 278 747	1
	C4	180 536	1
	C5	71 217 549	1
	C6	78 326	1
	C7	551 708-710	1
	C8	791 814	1
	C9	800-801 804 820 822	1
ELSC	C1	506 507	0.8
	C2	503 504	0.5
	C3	496 497 498 499 500 501 502	0.2
	C4	**73 75 76** 186 187 **256 257 259 557 708 709 741**	0.2
	C5	**704 705**	0.2
SCA-DB	C1	**80** 169 181 214 253 260 558 672 707 712 **739 745**	1
	C2	60 695 700	1
	C3	**71 217 549**	0.9
	C4	**218 258 278 747**	0.9
SCA-TM	C1	**71 217 549**	1
	C2	**218 747**	1
	C3	**218 258 278 747**	1
	C4	**258 747**	1
	C5	**278 747**	1
	C6	**218 258 278**	0.9
MI	NO MATRIX		

Table 5: **AATPase Rad3: comparing methodologies. Part 1.**
See legend of Table 1-Text S8. Continued in Table 6-Text S8.

AATPase Rad3 comparing methodologies			
Method	Cl	Positions	Sym
MST	C1	**69 71 73 75 76 176** 187 **217 256 257 259** 286 287 288 **319 549 557 708 709 741 797 801**	1
	C2	**81 316 317 711**	1
	C3	**800 804 820 822**	0.91
	C4	26 **80 131 132** 169 **171** 181 186 195 196 197 201 214 **218** 253 **258** 260 270 **278 318** 322 **553** 558 568 672 694 **704 705** 707 712 **739 745 747**	0.91
	C5	68 143 179 216	0.91
	C6	178 274 702	0.91
	C7	330 697	0.65
	C8	60 142 172 539 695 700	0.65
	C9	803 832	0.65
	C10	31 38	0.65
	C11	125 207	0.65
	C12	88 222 296	0.65
	C13	513 742	0.65
	C14	**180** 219 268 562	0.65
	C15	204 294	0.39
	C16	252 546	0.39
	C17	302 321 706	0.39
	C18	511 527	0.39
	C19	540 740	0.39
	C20	58 292	0.30
	C21	829 849	0.30
	C22	538 671	0.30
CTMP	NO MATRIX		

Table 6: **AATPase Rad3: comparing methodologies. Part 2.**

Table 5-Text S8 continued.

AATPase DEAD-box comparing methodologies			
Method	Cl	Position in Alignment	Sym
BIS, *pos*, $d = 0$	C1	34 73 75 76 256 257 259 318 319 709 797 804	1
BIS, *pos*, $d = 1$	C1	133 213	1
	C2	136 801	1
BIS, *blocks*, $d = 0$	C1	34 73 75-76 256-257 259 318-319 709 797 804	1
BIS, *blocks*, $d = 1$	C2	133 213	1
	C3	136 801	1
ELSC	NO CLUSTERS		
SCA-DB	C1	**136 801**	0.9
	C2	**133 213**	0.9
SCA-TM	C1	**136 801**	0.9
	C2	**133 213**	0.9
MI	NO MATRIX		
MST	C1	**34 73 75 76** 214 **256 257 259 318 319 709 797 804**	1
	C2	**133 213**	1
	C3	**136 801**	1
CTMP	NO CLUSTERS		

Table 7: **AATPase DEAD-box: comparing methodologies.**

See legend of Table 1-Text S8.

AATPase RecQ comparing methodologies			
Method	Cl	Positions	Sym
BIS, *pos*, $d = 0$	C1	126 317 332 554 706 707 708 716	1
	C2	34 72 75 76 78 129 133 136 256 257 259 318 319 552 699 709 741 801 803 804	1
BIS, *pos*, $d = 1$	C1	29 77 81 137 555 639 797 800 802	1
	C2	73 141 214 215 316 698	1
BIS, *blocks*, $d = 0$	C1	126 317-319 332 554 706-709 716	1
	C2	34 72 75-76 78 129 133 136 256-257 259 318-319 741 552 699 709 801 803-804	1
BIS, *blocks*, $d = 1$	C1	75-78 81 136-137 29 555 639 797 800-804	1
	C2	72-73 141 214-215 316 698-699	1
ELSC	C1	260 262	1
	C2	268 269	1
	C3	640 641	1
	C4	**706 707 708**	1
	C5	**800 802**	0.9
	C6	**214 215**	0.9
	C7	513 514	0.9
	C8	**34 72 75 76 78 129 133 136 256 257 259 318 319 552 699 709 741 801 803 804**	0.3
SCA-DB	C1	79 83 **126** 130 260 262 264 266 268 269 **317** 320 327 **332 554** 640 641 **706 707 708 716** 743 795 805	1
	C2	82 711	1
	C3	128 806	1
	C4	642 665	1
	C5	**29 81 137 555 639 797**	1
	C6	**29 77 81 137 555 639 797 800**	1
	C7	71 715	1
	C8	**77 555**	1
	C9	**555 800**	1
	C10	**73 141 214 215 316 698**	0.9
	C11	**29 77 81 137 555 639 797 800 802**	0.9
	C12	**29 77 81 137 639 797 800 802**	0.9
	C13	**77 639 800 802**	0.9
	C14	**77 81 800 802**	0.9
	C15	**77 137 800 802**	0.9
	C16	**555 802**	0.9
	C17	**77 797 800 802**	0.9
	C18	**29 77 800 802**	0.9

Table 8: **AATPase RecQ: comparing methodologies. Part 1.**

See legend of Table 1-Text S8.

AATPase RecQ comparing methodologies			
Method	Cl	Positions	Sym
SCA-TM	C1	79 83 **126** 130 260 262 264 266 268 269 **317** 320 327 **332 554** 640 641 **706 707 708 716** 743 795 805	1
	C2	79 83 **126** 260 262 264 266 268 269 **317** 320 327 **554** 640 641 **706 707 708 716** 743 795 805	1
	C3	79 83 **126** 260 262 264 266 268 269 **317** 320 327 **554** 640 **706 707 708 716** 743 795 805	1
	C4	79 83 260 262 264 266 268 269 **317** 327 **554** 640 **707 708 716** 743 795 805	1
	C5	79 83 **126** 260 262 264 266 269 **317** 327 **554** 640 **707 708 716** 743 795 805	1
	C6	79 83 260 262 264 266 269 **317** 327 **554** 640 **706 707 708 716** 743 795 805	1
	C7	79 83 260 262 264 266 269 **317** 320 327 **554** 640 **707 708 716** 743 795 805	1
	C8	79 83 260 264 266 269 327 **554** 640 641 **716** 743 795 805	1
	C9	82 711	1
	C10	**332 716**	1
	C11	130 **716**	1
	C12	128 806	1
	C13	642 665	1
	C14	71 715	1
	C15	79 83 **126** 130 260 262 264 266 268 269 **317** 320 327 **332 554** 640 641 **706 707 708** 743 795 805	0.9
	C16	130 269 **332**	0.9
	C17	130 **332** 795	0.9
	C18	**126** 130 262 268 **317** 320 **332** 641 **706 707 708**	0.9
	C19	130 **317 332** 641	0.9
	C20	130 262 **332** 641	0.9
	C21	**126** 130 268 320 **332** 641 **706**	0.9
	C22	130 **332** 641 **707**	0.9
	C23	130 **332** 641 **708**	0.9
	C24	130 **332** 743	0.9
	C25	130 266 **332**	0.9
	C26	130 327 **332**	0.9
	C27	130 **332** 805	0.9
	C28	79 130 **332**	0.9
	C29	83 130 **332**	0.9
	C30	130 260 **332**	0.9
	C31	130 264 **332**	0.9
	C32	130 **332 554**	0.9
	C33	130 **332** 640	0.9
	C34	**73 141 214 215 316 698**	0.9
	C35	**73 214 215 698**	0.9
	C36	**73 141 214 316 698**	0.9
	C37	**29 77 81 137 555 639 797 800**	0.9
	C38	**29 77 81 137 555 639 797 800 802**	0.9
	C39	**29 77 555 639 800 802**	0.9
MI	NO MATRIX		
MST	C1	**29 34 72 75 76 77 78 81 129 133 136 137 256 257 259 318 319 552 555 639 699 709 741 797 800 801 802 803 804**	1
	C2	**132** 793	1
	C3	**73** 79 83 **126** 130 **141 214 215** 260 262 264 266 268 269 **316 317** 320 327 **332 554** 640 641 **698 706 707 708 716** 743 795 805	0.82
	C4	80 84	0.82
	C5	815 840	0.64
	C6	44 82 711	0.64
	C7	71 124 272 668 715	0.64
	C8	304 513 514 518	0.55
	C9	695 859	0.55
	C10	263 638	0.55
CTMP	NO MATRIX		

Table 9: **AATPase RecQ: comparing methodologies. Part 2.**
See legend of Table 1-Text S8.

**AATPase Ski2-like comparing methodologies**			
Method	Cl	Positions	Sym
BIS, *pos*, $d = 0$	C1	72 75 76 77 129 256 257 259 317 635 639 640 700 709 711 800 801 802 803 804	1
	C2	71 701	1
BIS, *pos*, $d = 1$	C1	170 808	1
	C2	213 266 271 565	1
	C3	267 322 698 713 717 797 798	1
BIS, *blocks*, $d = 0$	C1	129 256-257 259 317 635 639-640 700 709 711 72 75-77 800-804	1
	C2	71-72 700-701	1
BIS, *blocks*, $d = 1$	C1	170 808	1
	C2	213 266 271 565	1
	C3	267 322 698 713 717 797-798	1
ELSC	C1	339 340 342	1
	C2	667 668	0.9
	C3	87 88	0.9
	C4	350 351	0.9
	C5	22 74	0.7
	C6	121 122	0.7
	C7	418 419 426	0.7
	C8	358 361	0.7
	C9	412 414 424	0.7
	C10	18 24	0.7
	C11	308 309	0.7
	C12	1 7	0.2
	C13	622 623	0.2
	C14	286 287 289 292	0.2
	C15	**565** 566	0.2
	C16	2 6	0.2
	C17	285 288 290 291 293 294 295 296 297 298 299 300 301 302 303 304 305 306	0.2
	C18	16 19	0.2
	C19	**72** 73 **75 76 77 256 257** 318 696 699 708 **709** 864	0.2
SCA-DB	C1	**322 698 713 717 797 798**	0.9
SCA-TM	C1	**322 797**	1
	C2	**322 698 713 717 797 798**	0.9
	C3	**322 698 713 717 798**	0.9
	C4	**698 713 717 797 798**	0.9
MI	NO MATRIX		
MST	C1	**71** 330 **701**	1
	C2	**72 75 76 77 129 256 257 259 267 317 322 635 639 640 698 700 709 711 713 717 797 798 800 801 802 803 804**	1
	C3	318 319	1
	C4	133 672	0.83
	C5	742 805	0.83
	C6	**170 808**	0.83
	C7	**213 266 271** 554 **565** 642	0.83
	C8	80 557	0.83
	C9	169 171 695	0.74
	C10	34 675	0.65
	C11	743 796	0.57
CTMP	NO MATRIX		

Table 10: **AATPase Ski2-like: comparing methodologies.**

See legend of Table 1-Text S8.

AATPase RigI-like comparing methodologies			
Method	Cl	Positions	Sym
BIS, pos, $d = 0$	C1	126 66 78	1
	C2	128 247 796 803	1
	C3	67 129 252 258 270 271 564 715 716 742 805 808	1
	C4	31 33 34 65 72 73 75 76 77 136 213 256 257 259 267 318 554 558 672 694 696 699 708 709 711 712 797 800 801 804	1
	C5	36 250 261 315 319 701	1
	C6	30 266 356	1
	C7	421 514	1
BIS, pos, $d = 1$	C1	134 553 707 80	1
	C2	169 217 279	1
	C3	63 68 186 211 263 312 323 339 340 342 357 379 706 799	1
	C4	214 806	1
	C5	71 221 316 364 793	1
	C6	62 253 746	1
	C7	255 328	1
	C8	317 552 664	1
	C9	365 551 744 814	1
	C10	555 669	1
	C11	57 83	1
BIS, $blocks$, $d = 0$	C1	75-78 126 65-66	1
	C2	128 247 796-797 803-804	1
	C3	67 129 252 256-259 270-271 564 715-716 742 804-805 808	1
	C4	31 65 72-73 75-77 136 213 256-257 259 267 318 33-34 554 558 672 694696 699 708-709 711-712 797 800-801 804	1
	C5	36 250 261 315 318-319 701	1
	C6	30-31 266-267 356	1
	C7	421 514	1
BIS, $blocks$, $d = 1$	C1	80 134 553-554 707-709	1
	C2	169 217 279	1
	C3	186 211 263 312 323 339-340 342 63 68 357 379 706 779-801	1
	C4	213-214 806	1
	C5	71-73 221 316 364 793	1
	C6	62 253 746	1
	C7	255-257 328	1
	C8	317-318 552 664	1
	C9	365 551 744 814	1
	C10	554-555 669	1
	C11	57 83	1
ELSC	C1	**339 340 342**	1
	C2	667 668	0.9
	C3	87 88	0.9
	C4	350 351	0.9
	C5	22 74	0.7
	C6	121 122	0.7
	C7	418 419 426	0.7
	C8	358 361	0.7
	C9	412 414 424	0.7
	C10	18 24	0.7
	C11	308 309	0.7
	C12	1 7	0.2
	C13	622 623	0.2
	C14	286 287 289 292	0.2
	C15	565 566	0.2
	C16	2 6	0.2
	C17	285 288 290 291 293 294 295 296 297 298 299 300 301 302 303 304 305 306	0.2
	C18	16 19	0.2
	C19	**72 73 75 76 77 256 257 318 696 699 708 709** 864	0.2

Table 11: **AATPase RigI-like: comparing methodologies. Part 1.**

See legend of Table 1-Text S8. Continued in Table 12-Text S8.

AATPase RigI-like comparing methodologies			
Method	Cl	Positions	Sym
	C1	**317 664**	1
	C2	**80 707**	1
	C3	**316 364**	1
	C4	**80 316 317 364 664**	0.9
	C5	**80 316 317 664 707**	0.9
SCA-DB	C6	**80 317 364 664 707**	0.9
	C7	**316 317 364 664 707**	0.9
	C8	**80 316 317 364 707**	0.9
	C9	**80 316 364 664 707**	0.9
	C10	254 **701**	0.8
	C1	**317 664**	1
	C2	**316 364**	1
	C3	**80 707**	1
	C4	**316 317 364 664 707**	0.9
	C5	**80 316 317 364 664**	0.9
SCA-TM	C6	**80 317 364 664 707**	0.9
	C7	**80 316 317 664 707**	0.9
	C8	**80 316 317 364 707**	0.9
	C9	**80 316 364 664 707**	0.9
	C10	254 **701**	0.8
	C1	**31 33 34** 60 **65 72 73 75 76 77 136 213 256 257 259 267 318 554 558 672 694 696**	1
		**699 708 709 711 712 797 800 801 804**	
	C2	**128** 561 **803**	1
	C3	**66 78 126**	1
	C4	**68 250 261 315 319 379** 745	1
	C5	70 **80 134 553 707 796**	0.82
	C6	**71** 124 170 **221 316** 321 **364 701 793**	0.82
	C7	**67 129 252 258 270 271 317** 343 **552 564 664** 698 **715 716 742 805 808**	0.82
MST	C8	**214** 396 548 **806** 841 853	0.82
	C9	**30** 82 **266 356** 383 400 **555** 581 639 **669** 817	0.82
	C10	61 **62** 225 **253** 362 **746** 860	0.82
	C11	**36** 59 **63** 64 87 88 165 **169 186 211 217 263** 268	0.73
		274 275 278 **279** 311 **312 323** 325 331 338 **339 340 342** 347 **357**	
		366 377 387 538 557 567 632 634 636 644 660 **706** 791 795 **799**	
	C12	85 127 **247 365 551** 667 668 671 695 **744** 747 **814** 818 828 852	0.73
	C13	133 314 320 550 560 577 638 675 710 821 846	0.73
	C14	56 84 94 131 149 168 171 178 228 251 260 324 327 334 336 341 378 390 418 419	0.55
		426 498 509 559 633 635 661 792 798 822 831 835 838 845 848 856 857 861	
MI	NO MATRIX		
CTMP	NO MATRIX		

Table 12: **AATPase RigI-like: comparing methodologies. Part 2.**
Table 11-Text S8 continued.

AATPase DEAH/RHA comparing methodologies			
Method	Cl	Positions	Sym
BIS, *pos*, $d = 0$	C1	33 73 75 76 78 129 140 256 257 260 261 266 319 699 745 791 798 801 808	1
BIS, *pos*, $d = 1$	C1	70 127	1
	C2	72 130 174 213 215 259 317 318 700 708 711 797 800 803 804	1
	C3	134 554	1
	C4	138 170	1
	C5	750 834	1
BIS, *blocks*, $d = 0$	C1	33 73 75-76 78 129 140 256-257 260-261 266 319 699 745 791 798 801 808	1
BIS, *blocks*, $d = 1$	C1	70 127	1
	C2	129-130 174 213 215 259-261 317-319	1
		72-73 699-700 708 711 797-798 800-801 803-804	1
	C3	134 554	1
	C4	138 170	1
	C5	750 833-834	1
ELSC	C1	702 707	0.9
	C2	**708 711**	0.8
	C3	**800 803 804**	0.8
	C4	**129 140 699 745 791 798 801 808**	0.1
SCA-DB	C1	**750 834** 837	1
	C2	**130 700 708 711 797 800 803 804**	0.9
	C3	141 833 845	0.9
	C4	131 702 707	0.9
SCA-TM	C1	**750 834** 837	1
	C2	131 707	1
	C3	**130 797 804**	0.9
	C4	**708 711 800**	0.9
	C5	141 845	0.9

Table 13: **AATPase DEAH/RHA: comparing methodologies. Part 1.**

See legend of Table 1-Text S8.

AATPase DEAH/RHA comparing methodologies			
Method	Cl	Positions	Sym
MST	C1	**33 70 73 75 76 78 127 129 140 256 257 260 261 266 319 699 745 791 798 801 808**	1
	C2	141 833 845	1
	C3	**72 130 174 213 215 259 317 318 700 708 711 797 800 803 804**	1
	C4	128 **138 170**	1
	C5	**134 554**	1
	C6	79 171	1
	C7	160 709	1
	C8	**33 70 73 75 76 78 127 129 140** 141 **256 257 260 261 266 319 699 745 791 798 801 808**	0.91
	C9	**33 70 73 75 76 78 127 129 140 256 257 260 261 266 319 699 745 791 798 801 808** 833	0.91
	C10	**33 70 73 75 76 78 127 129 140 256 257 260 261 266 319 699 745 791 798 801 808** 845	0.91
	C11	**33** 141 833 845	0.91
	C12	141 **257** 833 845	0.91
	C13	141 **260** 833 845	0.91
	C14	131 325 702 707	0.91
	C15	141 **261** 833 845	0.91
	C16	141 **266** 833 845	0.91
	C17	141 **319** 833 845	0.91
	C18	141 **699** 833 845	0.91
	C19	141 **745** 833 845	0.91
	C20	141 **791** 833 845	0.91
	C21	141 **798** 833 845	0.91
	C22	141 **801** 833 845	0.91
	C23	**70** 141 833 845	0.91
	C24	141 **808** 833 845	0.91
	C25	**73** 141 833 845	0.91
	C26	**75** 141 833 845	0.91
	C27	**76** 141 833 845	0.91
	C28	**78** 141 833 845	0.91
	C29	**127** 141 833 845	0.91
	C30	**129** 141 833 845	0.91
	C31	**140** 141 833 845	0.91
	C32	141 **256** 833 845	0.91
	C33	**750 834** 837	0.91
	C34	**72 750 834** 837	0.82
	C35	**130 750 834** 837	0.82
	C36	**174 750 834** 837	0.82
	C37	**213 750 834** 837	0.82
	C38	**215 750 834** 837	0.82
	C39	**259 750 834** 837	0.82
	C40	**317 750 834** 837	0.82
	C41	**318 750 834** 837	0.82
	C42	**700 750 834** 837	0.82
	C43	**708 750 834** 837	0.82
	C44	**711 750 834** 837	0.82
	C45	**750 797 834** 837	0.82
	C46	**750 800 834** 837	0.82
	C47	**750 803 834** 837	0.82
	C48	**750 804 834** 837	0.82
	C49	**72 130 174 213 215 259 317 318 700 708 711 750 797 800 803 804**	0.82
	C50	**72 130 174 213 215 259 317 318 700 708 711 797 800 803 804 834**	0.82
	C51	**72 130 174 213 215 259 317 318 700 708 711 797 800 803 804** 837	0.82
MI	NO MATRIX		
CTMP	NO CLUSTERS		

Table 14: **AATPase DEAH/RHA: comparing methodologies. Part 2.**

See legend of Table 1-Text S8.

AATPase NS3/NPH-II comparing methodologies			
Method	Cl	Positions	Sym
BIS, *pos*, $d = 0$	C1	22 37 122 128 211 264 265 312 342 346 551 813 838	1
	C2	124 711	1
	C3	73 75 76 129 170 213 256 257 259 318 319 568 745 797 800 801 803 804	1
	C4	130 327	1
	C5	131 267	1
	C6	72 132 810	1
	C7	140 214 664 667	1
	C8	8 18 536 537 674	1
	C9	78 81 210	1
	C10	215 258 562	1
	C11	254 262	1
	C12	317 554	1
	C13	320 322 699 709 743	1
	C14	321 666	1
	C15	553 802	1
	C16	677 806	1
	C17	695 701	1
BIS, *pos*, $d = 1$	C1	125 698	1
	C2	136 217 271 657 751 862	1
	C3	16 137	1
	C4	139 831 844	1
	C5	20 23 328	1
	C6	28 763	1
	C7	333 746	1
	C8	39 794	1
	C9	544 661	1
	C10	79 759	1
BIS, *blocks*, $d = 0$	C1	22 37 122 128-132 210-211 264-265 312 342 346 551 813 838	1
	C2	124 711	1
	C3	129 170 213 256-257 259 318-319	1
		73 75-76 568 745 797 800-801 803-804	
	C4	131 267	1
	C5	129-131 327	1
	C6	72-73 132 810	1
	C7	140 213-214 664-665 667	1
	C8	8 18 536-539 674	1
	C9	78 81 210	1
	C10	215 256-259 562	1
	C11	254 262	1
	C12	317-319 554	1
	C13	318-320 322 699 709 743	1
	C14	318-322 666	1
	C15	553 800-804	1
	C16	677 806	1
	C17	695 701	1
BIS, *blocks*, $d = 1$	C1	125 698-699	1
	C2	136 217 271 657-658 751 862	1
	C3	16 137	1
	C4	139 831 844	1
	C5	20 23 328	1
	C6	28 763	1
	C7	333 745-746	1
	C8	39 794	1
	C9	544 661	1
	C10	79 759	1
ELSC	C1	324 325	0.9
SCA-DB	C1	250 798	0.9
SCA-TM	C1	250 798	0.9

Table 15: **AATPase NS3/NPH-II: comparing methodologies. Part 1.**
See legend of Table 1-Text S8. Continued in Table 16-Text S8.

AATPase NS3/NPH-II comparing methodologies			
Method	Cl	Positions	Sym
MST	C1	**20 23** 71 84 **328**	1
	C2	**22 37** 60 **122 128 211** 255 **264 265 312** 330 **342 346** 544 **551** 661 **813**	1
	C3	121 **139**	1
	C4	**140 214 664 667**	1
	C5	**78 81 210**	1
	C6	314 **695 701**	1
	C7	**131 267**	1
	C8	127 270 565 799	1
	C9	126 658	1
	C10	**320 322 699 709 743**	1
	C11	**317 554**	1
	C12	**73 75 76 129 170 213 256 257 259 318 319 568 745 797** 798 **800 801 803 804**	1
	C13	43 795	1
	C14	68 **254 262**	1
	C15	**72 132 810**	1
	C16	**124 711**	1
	C17	**553 802**	1
	C18	**18** 57 **674**	1
	C19	**321 666**	1
	C20	**16 137**	1
	C21	39 794	1
	C22	331 **677 806** 819	1
	C23	**130 327**	1
	C24	**215 258 562**	1
	C25	144 247	0.90
	C26	209 694	0.90
	C27	**136 217** 324 325 **657** 811	0.90
	C28	710 747	0.90
	C29	659 752	0.90
	C30	249 334	0.90
	C31	42 266 339	0.90
	C32	123 796	0.81
	C33	19 34 35 66 260 338 341 540 542 543 815	0.81
	C34	33 69	0.81
MI	NO MATRIX		
CTMP	NO MATRIX		

Table 16: **AATPase NS3/NPH-II: comparing methodologies. Part 2.**
Table 15-Text S8 continued.

AATPase Swi2/Snf2 comparing methodologies			
Method	Cl	Positions	Sym
BIS, *pos*, $d = 0$	C1	73 75 76 77 136 256 257 320 804	1
BIS, *pos*, $d = 1$	C1	129 797 801 807	1
	C2	74 709	1
BIS, *blocks*, $d = 0$	C1	73 75-77 136 256-257 320 804	1
BIS, *blocks*, $d = 1$	C1	129 797 801 807	1
	C2	73-77 709	1
ELSC	C1	613 614 615 616 617 618 619	0.2
	C2	48 49	0
	C3	**76 77 256 257**	0
	C4	605 606 607 608 610 611	0
SCA-DB	NO CLUSTERS		
SCA-TM	NO CLUSTERS		
MST	C1	**73 74 75 76 77 136 256 257 320 709** 792 **804**	1
	C2	**73 75 76 77 136 256 257 320** 792 **804**	1
	C3	**74 709** 792	1
	C4	642 834	1
	C5	72 **129** 318 **797 801 807**	1
MI	NO MATRIX		
CTMP	NO CLUSTERS		

Table 17: **AATPase Swi2/Snf2: comparing methodologies.**

See legend of Table 1-Text S8.
