# Supplementary material for: Protein Fragments: Functional and Structural Roles of Their Coevolution Networks
Source: PLoS One. 2012 Nov 5;7(11):e48124. doi: 10.1371/journal.pone.0048124 (PMC3489791; doi:10.1371/journal.pone.0048124)
Supplement: Text S9 — Comparison of coevolution analysis methods on aligned sets of sequences representing the Amyloid -peptide. (PDF) [file pone.0048124.s011.pdf]

Supporting Information Text S9: comparison of coevolution analysis methods on aligned sets of sequences representing the Amyloid β-peptide

Amyloid beta peptide on 80 sequences				
Method	Cl	Positions	Sym	Env
BIS, *pos*, d_2^+	C1	12 17	1	1
	C2	11 14 16	1	1
	C3	19 26 27 28 29 30 31 32 33 34 35 36 37 38	1	1
	C4	18 20 21	0.47	0.08
	C5	12 18 20	0.42	0.5
	C6	17 18 20	0.42	0.5
	C7	22 39	0.05	0.08
	C8	40 41 42	1	1
	C9	18 20	1	1
BIS, *blocks*, d_2^+	C1	12 17	1	1
	C2	11-12 14 16-20	1	1
	C3	19 26-38	1	1
	C4	18-20 21	0.47	0.08
	C5	12 18-20	0.42	0.5
	C6	17 18-20	0.42	0.5
	C7	21-22 26-42	0.05	0.08
	C8	40 41 42	1	1
	C9	18 20	1	1
	S1	4 8	0.9	1
	S2	5 10	0.9	1
ELSC	S3	**11 14 16**	0.9	1
	S4	**18 20**	0.9	1
	S5	**12 17 19 26 27 28 29 30 31 32 33 34 35 36 37 38 40 41 42**	0.09	1
SCA-DB	NO CLUSTERS			
SCA-TM	NO CLUSTERS			
MST	S1	**12 17**	1	1
	S2	**19 26 27 28 29 30 31 32 33 34 35 36 37 38**	1	1
MI	S1	**11 14 15 16 18 20**	0.8	1
	S2	**12 17 19 26 27 28 29 30 31 32 33 34 35 36 37 38 40 41 42**	0.3	1
CTMP	NO CLUSTERS			

Table 1: **Amyloid beta peptide: comparing methodologies run over 80 sequences.**
For each method, we report the clusters of co-evolving residues detected by the clustering algorithm CLAG (with environmental score equal 1, symmetric score >0 and $\Delta = 0.05$). For BIS we selected clusters with symmetric score $= 1$ only. For each methodology we report the number of sequences (Al), the names of the clusters (Cl), the positions on the PDB structure (Positions). Results are reported for BIS run on blocks and on single alignment positions. SCA, ELSC, MI, MST and CTMP methods are compared to BIS execution when considering alignment positions (*pos*). Common residues predicted by both methodologies are highlighted in bold. Green residues are those belonging to the Walker-A. NO MATRIX means that the method did not output any co-evolution pair; NO CLUSTERS means that the method output co-evolution pairs that could not be clustered together. Notice that in this analysis, for all methods (including BIS), we report the clusters of coevolving residues detected by the clustering algorithm CLAG with environmental and symmetric scores >0 and $\Delta = 0.05$.

**Amyloid beta peptide on 16 sequences**				
Method	Cl	Positions	Sym	Env
BIS, *pos*, d_2^+	C1	18 20	1	1
	C2	19 26 27 28 29 30 31 32 33 34 35 36 37 38	1	1
	C3	11 14 16	1	1
	C4	12 17	1	1
	C5	40 41 42	1	1
BIS, *blocks*, d_2+	C1	18 20	1	1
	C2	19 26-38	1	1
	C3	11-12 14 16-20	1	1
	C4	12 17	1	1
	C5	40 41 42	1	1
SCA-DB	NO CLUSTERS			
SCA-TM	NO CLUSTERS			
ELSC	S1	**11 14 16**	1	1
	S2	**18 20**	1	1
	S3	13 **17**	0.90	1
	S4	**12 17 19 26 27 28 29 30 31 32 33 34 35 36 37 38 40 41 42**	0.14	1
MI	S1	**11 14 16 18**	0.71	1
	S2	**11 14 16 18 20**	0.71	1
	S3	**11 14 16 20**	0.71	1
	S4	**18 20**	0.52	1
MST	S1	**19 26 27 28 29 30 31 32 33 34 35 36 37 38**	1	1
	S2	**12 17**	0.14	1
	S3	**39 40 41 42**	0.14	1
	S4	**12** 25	0.04	1
	S5	**12 17** 25	0.04	1
	S6	25 **17**	0.04	1
CTMP	NO CLUSTERS			

Table 2: **Amyloid beta peptide: comparing methodologies run over 16 sequences.**
See legend of Table 1-Text S9.
