# Supplementary material for: Protein Fragments: Functional and Structural Roles of Their Coevolution Networks
Source: PLoS One. 2012 Nov 5;7(11):e48124. doi: 10.1371/journal.pone.0048124 (PMC3489791; doi:10.1371/journal.pone.0048124)
Supplement: Text S10 — Validation of performance of different coevolution analysis methods. (PDF) [file pone.0048124.s012.pdf]

Supporting Information Text S10: validation of performance of different coevolution analysis methods

	Size	API	Pos	#Exp	BIS	TP	FN	FP	TN	Prob	Sen*	Spe*	Acc*	PPV*	Alignment	pdb
Amyloid	80	0.87	43	25	27	22	3	5	13	$7.79e^{-5}$	0.88	0.72	0.81	0.81	PFAM PF03494	1aml
	16	0.77	43	25	24	20	5	4	14	$2.16e^{-4}$	0.80	0.78	0.79	0.83		
MukB - Walker A	200	0.84	234	8	8	7	1	1	225	$9.16e^{-12}$	0.88	1	0.99	0.88	PFAM PF04310	1qhl:A
	205	0.81	274	8	4	4	4	0	266	$3.05e^{-7}$	0.5	1	0.99	1		
	49	0.77	234	8	13	7	1	6	220	$1.92e^{-9}$	0.88	0.97	0.97	0.54		
	54	0.6	274	8	4	4	4	0	266	$3.05e^{-7}$	0.50	1	0.99	1		
Protein A - B domain	20	0.71	57	36	30	25	11	5	16	$9.89e^{-4}$	0.69	0.76	0.72	0.83	PFAM PF02216	1bdd:A
	28	0.6	62	36	5	3	33	2	24	0.65	0.08	0.92	0.44	0.60		
	452	0.82	57	36	30	25	11	5	16	$9.89e^{-4}$	0.69	0.76	0.72	0.83		
	490	0.75	62	36	5	3	33	2	24	0.65	0.08	0.92	0.44	0.60		
Upf1	18	0.58	677	64	26	18	46	8	605	$2.93e^{-14}$	0.28	0.99	0.92	0.69	[FW *et al.* 2010]	2gjk
RecD	6	0.51	642	55	73	32	23	41	546	$1.63e^{-19}$	0.58	0.93	0.90	0.44	[FW *et al.* 2010]	1p4d
UvrD/rep	8	0.53	661	62	38	30	32	8	591	$1.89e^{-27}$	0.48	0.99	0.94	0.79	[FW *et al.* 2010]	2is1
Rad3	9	0.52	592	55	62	28	27	34	503	$3.53e^{-16}$	0.51	0.94	0.90	0.45	[FW *et al.* 2010]	3crv
DEAD-box	67	0.61	624	66	16	16	50	0	558	$4.11e^{-17}$	0.24	1	0.92	1	[FW *et al.* 2010]	3i5x
RecQ	9	0.61	514	65	42	31	34	11	438	$1.25e^{-22}$	0.48	0.98	0.91	0.74	[FW *et al.* 2010]	2v1x
Ski2-like	13	0.51	686	62	35	22	40	13	611	$1.47e^{-16}$	0.35	0.98	0.92	0.63	[FW *et al.* 2010]	2xgj
RigI-like	6	0.47	658	64	104	40	24	64	530	$9.42e^{-20}$	0.63	0.89	0.87	0.38	[FW *et al.* 2010]	1wp9
DEAH-RHA	24	0.65	562	63	43	29	34	14	485	$8.06e^{-21}$	0.46	0.97	0.91	0.67	[FW *et al.* 2010]	3kx2
NS3-NPH-II	11	0.6	479	62	101	36	26	65	352	$4.83e^{-12}$	0.58	0.84	0.81	0.36	[FW *et al.* 2010]	1cu1
Swi2-Snf2	45	0.51	714	76	15	14	62	1	637	$1.05e^{-13}$	0.18	1	0.91	0.93	[FW *et al.* 2010]	3dmq

Table 1: **Validation of performance of coevolution analysis realized with BIS**.
Percentage of identity for the alignment (API), number of sequences (Size), alignment length (where all gapped positions, if any, are eliminated; Pos), number of experimentally confirmed residues (#Exp), number of residues identified by coevolution analysis (BIS), number of true positives (TP) computed by intersecting #Exp and BIS, probability of predicting TP residues out of #Exp by selecting BIS residues within Len residues (Prob), Sensitivity (Sen), Specificity (Spe), Accuracy (Acc), Positive Predictive Value (PPV) are given. For Amyloid, experimentally validated residues (#Exp) have been obtained from the different sources cited in the text; for Protein A we considered hotspots together with the large peptide fragment [25-59] and residues 14, 17 to have high Φ-value; MukB analysis is evaluated on Walker-A detection; the AATPase family is evaluated on all motifs described in (Fairman-Williams et al. 2010). Sen, Spe, Acc, PPV have been evaluated with respect to experimentally validated residues; they are marked with * to remind this. The same is true for Prob.

	Size	API	Pos	#Exp	MST	TP	FN	FP	TN	Prob	Sen*	Spe*	Acc*	PPV*	Alignment	pdb
Amyloid	80	0.87	43	25	16	12	13	4	14	0.08	0.48	0.78	0.60	0.75	PFAM PF03494	`1aml`
	16	0.77	43	25	21	16	9	5	13	0.02	0.64	0.72	0.67	0.76		
MukB -	200	0.84	234	8	-	-	-	-	-	-	-	-	-	-	PFAM PF04310	`1qhl:A`
Walker A	205	0.81	274	8	6	5	3	1	265	$2.68e^{-8}$	0.63	1.00	0.99	0.83		
	49	0.77	234	8	-	-	-	-	-	-	-	-	-	-		
	54	0.6	274	8	6	5	3	1	265	$2.68e^{-8}$	0.63	1.00	0.99	0.83		
Protein A	20	0.71	57	36	13	6	30	7	14	0.96	0.17	0.67	0.35	0.46	PFAM PF02216	`1bdd:A`
B domain	28	0.6	62	36	5	3	33	2	24	0.65	0.08	0.92	0.44	0.60		
	452	0.82	57	36	17	7	29	10	11	0.99	0.19	0.52	0.32	0.41		
	490	0.75	62	36	9	3	33	6	20	0.98	0.08	0.77	0.37	0.33		
Upf1	18	0.58	677	64	35	21	43	14	599	$9.66e^{-15}$	0.33	0.98	0.92	0.60	[FW*et al.* 2010]	`2gjk`
RecD	6	0.51	642	55	63	26	29	37	550	$1.48e^{-14}$	0.47	0.94	0.90	0.41	[FW*et al.* 2010]	`1p4d`
UvrD/rep	8	0.53	661	62	59	31	31	28	571	$4.01e^{-20}$	0.50	0.95	0.91	0.53	[FW*et al.* 2010]	`2is1`
Rad3	9	0.52	592	55	110	31	24	79	458	$3.10e^{-11}$	0.56	0.85	0.83	0.28	[FW*et al.* 2010]	`3crv`
DEAD-box	67	0.61	624	66	17	17	49	0	558	$3.38e^{-18}$	0.26	1.00	0.92	1.00	[FW*et al.* 2010]	`3i5x`
RecQ	9	0.61	514	65	81	38	27	43	406	$4.21e^{-18}$	0.58	0.90	0.86	0.47	[FW*et al.* 2010]	`2v1x`
Ski2-like	13	0.51	686	62	53	32	30	21	603	$5.15e^{-24}$	0.52	0.97	0.93	0.60	[FW*et al.* 2010]	`2xgj`
RigI-like	6	0.47	658	64	207	48	16	159	435	$4.98e^{-14}$	0.75	0.73	0.73	0.23	[FW*et al.* 2010]	`1wp9`
DEAH-RHA	24	0.65	562	63	55	33	30	22	477	$1.29e^{-21}$	0.52	0.96	0.91	0.60	[FW*et al.* 2010]	`3kx2`
NS3-NPH-II	11	0.6	479	62	132	41	21	91	326	$8.67e^{-12}$	0.66	0.78	0.77	0.31	[FW*et al.* 2010]	`1cu1`
Swi2-Snf2	45	0.51	714	76	20	15	61	5	633	$6.81e^{-12}$	0.20	0.99	0.91	0.75	[FW*et al.* 2010]	`3dmq`

Table 2: **Validation of performance of coevolution analysis realized with MST**.
See legend of Table 1-Text 10.

	**Size**	**API**	**Pos**	**#Exp**	**ELSC**	**TP**	**FN**	**FP**	**TN**	**Prob**	**Sen***	**Spe***	**Acc***	**PPV***	**Alignment**	**pdb**
Amyloid	80	0.87	43	25	28	20	5	8	10	$1.83e^{-2}$	0.80	0.56	0.70	0.71	PFAM PF03494	`1aml`
	16	0.77	43	25	25	21	4	4	14	$6.68e^{-5}$	0.84	0.78	0.81	0.84		
MukB	200	0.84	234	8	13	7	1	6	220	$1.93e^{-9}$	0.88	0.97	0.97	0.54	PFAM PF04310	`1qhl:A`
Walker A	205	0.81	274	8	6	5	3	1	265	$2.68e^{-8}$	0.63	1	0.99	0.83		
	49	0.77	234	8	13	7	1	6	220	$1.93e^{-9}$	0.88	0.97	0.97	0.54		
	54	0.6	274	8	-	-	-	-	-	-	-	-	-	-		
Protein A	20	0.71	57	36	30	25	11	5	16	$9.89128e^{-4}$	0.69	0.76	0.72	0.83	PFAM PF02216	`1bdd:A`
B domain	28	0.6	62	36	7	5	31	2	24	0.36	0.14	0.92	0.47	0.71		
	452	0.82	57	36	33	24	12	9	12	0.07	0.67	0.57	0.63	0.73		
	490	0.75	62	36	2	2	34	0	26	0.33	0.06	1	0.45	1		
Upf1	18	0.58	677	64	22	14	50	8	605	$2.00e^{-10}$	0.22	0.99	0.91	0.64	[FW*et al.* 2010]	`2gjk`
RecD	6	0.51	642	55	87	20	35	67	520	$5.78e^{-6}$	0.36	0.89	0.84	0.23	[FW*et al.* 2010]	`1p4d`
UvrD/rep	8	0.53	661	62	65	22	40	43	556	$3.90e^{-9}$	0.35	0.93	0.87	0.34	[FW*et al.* 2010]	`2is1`
Rad3	9	0.52	592	55	25	10	45	15	522	$2.36e^{-5}$	0.18	0.97	0.90	0.40	[FW*et al.* 2010]	`3crv`
DEAD-box	67	0.61	624	66	-	-	-	-	-	-	-	-	-	-	[FW*et al.* 2010]	`3i5x`
RecQ	9	0.61	514	65	35	25	40	10	439	$2.47e^{-17}$	0.38	0.98	0.90	0.71	[FW*et al.* 2010]	`2v1x`
Ski2-like	13	0.51	686	62	70	13	49	57	567	$6.13e^{-3}$	0.21	0.91	0.85	0.19	[FW*et al.* 2010]	`2xgj`
RigI-like	6	0.47	658	64	70	13	51	57	537	0.01	0.20	0.90	0.84	0.19	[FW*et al.* 2010]	`1wp9`
DEAH-RHA	24	0.65	562	63	15	11	52	4	495	$1.46e^{-8}$	0.17	0.99	0.90	0.73	[FW*et al.* 2010]	`3kx2`
NS3-NPH-II	11	0.6	479	62	2	0	62	2	415	1	0	1	0.87	0	[FW*et al.* 2010]	`1cu1`
Swi2-Snf2	45	0.51	714	76	19	4	72	15	623	0.13	0.05	0.98	0.88	0.21	[FW*et al.* 2010]	`3dmq`

Table 3: **Validation of performance of coevolution analysis realized with ELSC**. See legend of Table 1-Text 10.

	Size	API	Pos	#Exp	MI	TP	FN	FP	TN	Prob	Sen*	Spe*	Acc*	PPV*	Alignment	pdb
Amyloid	80	0.87	43	25	25	20	5	5	13	$8.15e^{-4}$	0.80	0.72	0.77	0.80	PFAM PF03494	1aml
	16	0.77	43	25	5	5	20	0	18	0.06	0.20	1	0.53	1		
MukB - Walker A	200	0.81	274	8	-	-	-	-	-	-	-	-	-	-	PFAM PF04310	1qhl:A
	205	0.81	274	8	5	2	6	3	263	0.007	0.25	0.99	0.97	0.40		
	49	0.77	234	8	-	-	-	-	-	-	-	-	-	-		
	54	0.6	274	8	5	2	6	3	263	0.007	0.25	0.99	0.97	0.40		
Protein A B domain	20	0.71	57	36	2	2	34	0	21	0.39	0.06	1	0.40	1	PFAM PF02216	1bdd:A
	28	0.6	62	36	32	25	11	7	19	$1.02e^{-3}$	0.69	0.73	0.71	0.78		
	452	0.82	36	14	10	26	4	17	0.34	0.28	0.81	0.47	0.71			
	490	0.75	62	36	35	26	10	9	17	$3.4e^{-3}$	0.72	0.65	0.69	0.74		

Table 4: **Validation of performance of coevolution analysis realized with MI**.
See legend of Table 1-Text 10.

	Size	API	Pos	#Exp	SCA-DB	TP	FN	FP	TN	Prob	Sen*	Spe*	Acc*	PPV*	Alignment	pdb
Amyloid	80	0.87	43	25	-	-	-	-	-	-	-	-	-	-	PFAM PF03494	`1aml`
	16	0.77	43	25	-	-	-	-	-	-	-	-	-	-		
MukB - Walker A	200	0.84	234	8	8	7	1	1	225	$9.16e^{-12}$	0.88	1	0.99	0.88	PFAM PF04310	`1qhl:A`
	205	0.81	274	8	-	-	-	-	-	-	-	-	-	-		
	49	0.77	234	8	8	7	1	1	225	$9.16e^{-12}$	0.88	1	0.99	0.88		
	54	0.6	274	8	-	-	-	-	-	-	-	-	-	-		
Protein A - B domain	20	0.71	57	36	-	-	-	-	-	-	-	-	-	-	PFAM PF02216	`1bdd:A`
	28	0.6	62	36	5	3	33	2	24	0.65	0.08	0.92	0.44	0.60		
	452	0.82	57	36	29	22	14	7	14	$3.97e^{-2}$	0.61	0.67	0.63	0.76		
	490	0.75	62	36	16	13	23	3	23	$2.70e^{-2}$	0.36	0.88	0.58	0.81		
Upf1	18	0.58	677	64	8	4	60	4	609	$3.82e^{-3}$	0.06	0.99	0.91	0.50	[FW*et al.* 2010]	`2gjk`
RecD	6	0.51	642	55	12	7	48	5	582	$1.32e^{-5}$	0.13	0.99	0.92	0.58	[FW*et al.* 2010]	`1p4d`
UvrD/rep	8	0.53	661	62	18	8	54	10	589	$7.95e^{-5}$	0.13	0.98	0.90	0.44	[FW*et al.* 2010]	`2is1`
Rad3	9	0.52	592	55	22	5	50	17	520	0.04	0.09	0.97	0.89	0.23	[FW*et al.* 2010]	`3crv`
DEAD-box	67	0.61	624	66	4	4	62	0	558	$1.15e^{-4}$	0.06	1	0.90	1	[FW*et al.* 2010]	`3i5x`
RecQ	9	0.61	514	65	47	18	47	29	420	$1.92e^{-6}$	0.28	0.94	0.85	0.38	[FW*et al.* 2010]	`2v1x`
Ski2-like	13	0.51	686	62	6	3	59	3	621	0.016	0.05	1	0.91	0.50	[FW*et al.* 2010]	`2xgj`
RigI-like	6	0.47	658	64	8	3	61	5	589	0.03	0.05	0.99	0.90	0.38	[FW*et al.* 2010]	`1wp9`
DEAH-RHA	24	0.65	562	63	17	10	53	7	492	$1.64e^{-6}$	0.16	0.99	0.89	0.59	[FW*et al.* 2010]	`3kx2`
NS3-NPH-II	11	0.6	479	62	2	1	61	1	416	0.24	0.02	1	0.87	0.50	[FW*et al.* 2010]	`1cu1`
Swi2-Snf2	45	0.51	714	76	-	-	-	-	-	-	-	-	-	-	[FW*et al.* 2010]	`3dmq`

Table 5: **Validation of performance of coevolution analysis realized with SCA-DB**.
See legend of Table 1-Text 10.

	Size	API	Pos	#Exp	SCA-TM	TP	FN	FP	TN	Prob	Sen*	Spe*	Acc*	PPV*	Alignment	pdb
Amyloid	80	0.87	43	25	-	-	-	-	-	-	-	-	-	-	PFAM PF03494	`1aml`
	16	0.77	43	25	-	-	-	-	-	-	-	-	-	-		
MukB - Walker A	200	0.84	234	8	8	7	1	1	225	$9.16e^{-12}$	0.88	1	0.99	0.88	PFAM PF04310	`1qhl:A`
	205	0.81	274	8	-	-	-	-	-	-	-	-	-	-		
	49	0.77	234	8	8	7	1	1	225	$9.16e^{-12}$	0.88	1	0.99	0.88		
	54	0.6	274	8	-	-	-	-	-	-	-	-	-	-		
Protein A B domain	20	0.71	57	36	-	-	-	-	-	-	-	-	-	-	PFAM PF02216	`1bdd:A`
	28	0.6	62	36	5	3	33	2	24	0.65	0.08	0.92	0.44	0.60		
	452	0.82	57	36	28	21	15	7	14	$6.05e^{-2}$	0.58	0.67	0.61	0.75		
	490	0.75	62	36	11	7	29	4	22	0.47	0.19	0.85	0.47	0.64		
Upf1	18	0.58	677	64	5	2	62	3	610	0.073	0.03	1	0.90	0.40	[FW*et al.* 2010]	`2gjk`
RecD	6	0.51	642	55	10	6	49	4	583	$4.88e^{-5}$	0.11	0.99	0.92	0.60	[FW*et al.* 2010]	`1p4d`
UvrD/rep	8	0.53	661	62	16	8	54	8	591	$2.73e^{-5}$	0.13	0.99	0.91	0.50	[FW*et al.* 2010]	`2is1`
Rad3	9	0.52	592	55	7	1	54	6	531	0.50	0.02	0.99	0.90	0.14	[FW*et al.* 2010]	`3crv`
DEAD-box	67	0.61	624	66	4	4	62	0	558	$1.15e^{-4}$	0.06	1	0.90	1	[FW*et al.* 2010]	`3i5x`
RecQ	9	0.61	514	65	47	18	47	29	420	$1.92e^{-6}$	0.28	0.94	0.85	0.38	[FW*et al.* 2010]	`2v1x`
Ski2-like	13	0.51	686	62	6	3	59	3	621	0.01	0.05	1	0.91	0.50	[FW*et al.* 2010]	`2xgj`
RigI-like	6	0.47	658	64	8	3	61	5	589	0.03	0.05	0.99	0.90	0.38	[FW*et al.* 2010]	`1wp9`
DEAH-RHA	24	0.65	562	63	13	8	55	5	494	$1.34e^{-5}$	0.13	0.99	0.89	0.62	[FW*et al.* 2010]	`3kx2`
NS3-NPH-II	11	0.6	479	62	2	1	61	1	416	0.24	0.02	1	0.87	0.50	[FW*et al.* 2010]	`1cu1`
Swi2-Snf2	45	0.51	714	76	-	-	-	-	-	-	-	-	-	-	[FW*et al.* 2010]	`3dmq`

Table 6: **Validation of performance of coevolution analysis realized with SCA-TM.** See legend of Table 1-Text 10.

	Size	API	Pos	#Exp	ConSurf	TP	FN	FP	TN	Prob	Sen*	Spe*	Acc*	PPV*	Alignment	pdb
Amyloid	80	0.87	43	25	17	13	12	4	14	$4.76e^{-2}$	0.52	0.78	0.63	0.76	PFAM PF03494	`1aml`
MUKB	200	0.84	234	8	106	8	0	98	128	$1.53e^{-3}$	1	0.57	0.58	0.08	PFAM PF04310	`1qhl:A`
PABD	52	0.82	57	36	26	22	14	4	17	$2.13e^{-3}$	0.61	0.81	0.68	0.85	PFAM PF02216	`1bdd:A`
UPF1	18	0.58	677	64	110	57	7	53	560	$6.39e^{-44}$	0.89	0.91	0.91	0.52	[FW *et al.* 2010]	`2gjk`
DEAD-box	67	0.61	624	66	110	66	0	44	514	$6.95e^{-60}$	1	0.92	0.93	0.60	[FW *et al.* 2010]	`3i5x`
Ski2-like	13	0.51	686	62	134	62	0	72	552	$7.85e^{-51}$	1	0.88	0.90	0.46	[FW *et al.* 2010]	`2xgj`

Table 7: **Validation of performance of conservation analysis realized with ConSurf.**
See legend of Table 1-Text 10.

	Size	API	Pos	#Exp	ET	TP	FN	FP	TN	Prob	Sen*	Spe*	Acc*	PPV*	Alignment	pdb
Amyloid	80	0.87	43	25	16	13	12	3	15	$1.88e^{-2}$	0.52	0.83	0.65	0.81	PFAM PF03494	`1aml`
MUKB	200	0.84	234	8	109	8	0	101	125	$1.92e^{-3}$	1	0.55	0.57	0.07	PFAM PF04310	`1qhl:A`
PABD	452	0.82	57	36	37	26	10	11	10	0.11	0.72	0.48	0.63	0.70	PFAM PF02216	`1bdd:A`
UPF1	18	0.58	677	64	135	54	10	81	532	$2.19e^{-32}$	0.84	0.87	0.87	0.40	[FW *et al.* 2010]	`2gjk`
Ski2-like	13	0.51	686	62	196	62	0	134	490	$6.16e^{-38}$	1	0.79	0.80	0.32	[FW *et al.* 2010]	`2xgj`

Table 8: **Validation of performance of conservation analysis realized with ET**.
See legend of Table 1-Text 10.

	Size	API	Pos	#Exp	Rate4Site	TP	FN	FP	TN	Prob	Sen*	Spe*	Acc*	PPV*	Alignment	pdb
Amyloid	80	0.87	43	25	26	21	4	5	13	$2.75e^{-4}$	0.84	0.72	0.79	0.81	PFAM PF03494	1qhl:A
MUKB	200	0.84	234	8	83	7	1	76	150	$3.37e^{-3}$	0.88	0.66	0.67	0.08	PFAM PF04310	1qhl:A
PABD	452	0.82	57	36	0	0	36	0	21	1	0	1	0.63	0	PFAM PF02216	1bdd:A
UPF1	18	0.58	677	64	23	13	51	10	603	$7.96e^{-9}$	0.20	0.98	0.91	0.57	[FW *et al.* 2010]	2gjk
RECD	6	0.51	642	55	5	3	52	2	585	0.005	0.05	1	0.92	0.60	[FW *et al.* 2010]	1p4d
UvrD/rep	8	0.53	661	62	29	10	52	19	580	$1.24e^{-4}$	0.16	0.97	0.89	0.34	[FW *et al.* 2010]	2is1
Rad3	9	0.52	592	55	2	0	55	2	535	1	0	1	0.90	0	[FW *et al.* 2010]	3crv
DEAD-box	67	0.61	624	66	33	15	51	18	540	$1.15e^{-7}$	0.23	0.97	0.89	0.45	[FW *et al.* 2010]	3i5x
RecQ	9	0.61	514	65	24	20	45	4	445	$4.47e^{-16}$	0.31	0.99	0.90	0.83	[FW *et al.* 2010]	2v1x
Ski2-like	13	0.51	686	62	36	20	42	16	608	$1.49e^{-13}$	0.32	0.97	0.92	0.56	[FW *et al.* 2010]	2xgj
RigI-like	6	0.47	658	64	8	5	59	3	591	$3.33e^{-4}$	0.08	0.99	0.91	0.63	[FW *et al.* 2010]	1wp9
DEAH-RHA	24	0.65	562	63	41	26	37	15	484	$1.86e^{-17}$	0.41	0.97	0.91	0.63	[FW *et al.* 2010]	3kx2
NS3-NPH-II	11	0.6	479	62	9	7	55	2	415	$1.30e^{-5}$	0.11	1	0.88	0.78	[FW *et al.* 2010]	1cu1
Swi2-Snf2	45	0.51	714	76	34	22	54	12	626	$4.02e^{-15}$	0.29	0.98	0.91	0.65	[FW *et al.* 2010]	3dmq

Table 9: **Validation of performance of conservation analysis realized with Rate4Site**.
See legend of Table 1-Text 10.
