# Supplementary material for: Protein Fragments: Functional and Structural Roles of Their Coevolution Networks
Source: PLoS One. 2012 Nov 5;7(11):e48124. doi: 10.1371/journal.pone.0048124 (PMC3489791; doi:10.1371/journal.pone.0048124)
Supplement: Text S11 — Conservation analysis of the protein families analyzed in the article. (PDF) [file pone.0048124.s013.pdf]

Supporting Information Text S11: conservation analysis of the protein families analyzed in the article

Conservation analysis of Amyloid - 80 sequences	
**Method**	**List of conserved residues**
Rate4Site	11 12 14 16 17 18 19 21 23 26 27 28 29 30 31 32 33 34 35 36 37 38 39 40 41 42
ConSurf	12 17 19 26 27 28 29 30 31 32 33 34 35 36 37 38 40
ET	17 19 26 27 28 29 30 31 32 33 34 35 36 37 38 40

Table 1: **Conservation analysis for the Amyloid set of 80 sequences.**
List of residues resulting from the conservation analysis of the protein family realized with Rate4Site (score of conserved positions ≤ 0.12), ConSurf (conserved residues have rank 8 and 9) and ET methods.

Conservation analysis of MukB - 200 sequences	
**Method**	**List of conserved residues**
Rate4Site	1 4 6 9 11 14 16 17 18 19 20 22 23 26 29 30 31 32 33 34 35 36 37 39 40 41 42 44 45 52 53 54 57 58 60 61 62 63 64 65 66 67 72 73 74 76 77 79 80 81 84 94 95 99 108 112 113 115 116 121 175 179 182 189 193 194 200 202 203 204 205 207 208 209 210 213 216 217 218 221 222 223 225
ConSurf	4 5 6 9 11 14 15 16 17 18 19 20 21 22 23 24 26 29 30 31 32 33 34 35 36 37 38 39 40 41 42 44 45 48 49 50 51 52 53 54 55 57 58 77 79 80 81 84 86 87 90 94 95 98 99 104 106 107 108 110 111 112 113 115 116 117 121 133 145 147 154 170 175 179 182 187 188 189 193 194 196 198 199 200 201 202 203 204 205 206 207 208 209 210 211 213 214 216 217 218 219 220 221 222 223 225
ET	4 5 6 9 10 11 14 15 16 17 18 19 20 21 22 23 24 26 28 29 30 31 32 33 34 35 36 37 38 39 40 41 42 44 45 47 49 51 52 53 54 57 58 77 79 80 81 84 86 87 90 94 95 98 99 106 107 108 110 111 112 113 115 116 117 121 145 146 147 154 165 166 170 173 174 175 178 179 182 188 189 193 194 196 197 198 200 201 202 203 204 205 206 207 208 209 210 211 213 214 216 217 218 219 220 221 222 223 225

Table 2: **Conservation analysis for the MukB set of 200 sequences.**
List of residues resulting from the conservation analysis of the protein family realized with Rate4Site (score of conserved positions ≤ 0.12), ConSurf (conserved residues have rank 8 and 9) and ET methods.

Conservation analysis of Protein A - B domain - 452 sequences	
**Method**	**List of conserved residues**
Rate4Site	no residues
ConSurf	11 13 14 15 18 22 23 27 29 30 31 32 33 34 35 36 37 38 39 40 42 46 48 49 51 52
ET	10 11 13 14 15 18 21 22 23 24 27 28 29 31 32 33 34 35 36 37 38 39 40 41 42 44 46 48 49 50 51 52 53 56 57 58 59

Table 3: **Conservation analysis for the Protein A - B domain set of 452 sequences.**
List of residues resulting from the conservation analysis of the protein family realized with Rate4Site (score of conserved positions ≤ 0.12), ConSurf (conserved residues have rank 8 and 9) and ET methods.

Conservation analysis of Upf1 - 18 sequences	
**Method**	**List of conserved residues**
Rate4Site	475 478 496 498 499 543 614 634 635 660 663 664 696 701 764 767 797 828 838 840 859 862 863
ConSurf	471 472 474 475 478 490 492 493 494 495 496 497 498 499 501 505 506 517 518 521 522 523 524 526 527 528 543 612 613 616 633 634 636 637 638 640 643 649 650 658 659 660 661 662 663 665 666 667 668 675 680 683 684 687 698 701 702 703 704 705 708 713 717 721 745 753 763 766 769 770 773 790 791 792 793 794 795 796 799 800 823 825 826 827 829 830 831 833 837 838 840 842 843 844 845 851 852 853 855 858 860 861 862 864 865 866 867 874 875 879
ET	471 472 474 475 478 488 489 490 491 492 493 494 495 496 497 498 499 501 502 518 519 521 522 523 524 526 527 528 530 543 549 550 559 561 562 603 614 616 617 620 622 624 625 630 634 636 637 640 643 644 645 649 661 662 663 665 666 667 668 679 680 683 684 685 686 687 688 695 697 698 701 702 703 704 706 708 711 713 716 717 719 721 723 724 739 745 748 753 755 756 761 763 765 766 769 770 774 790 791 794 795 796 797 798 799 800 802 818 825 826 827 829 830 831 832 833 834 835 837 841 842 843 845 850 851 852 853 857 858 859 860 861 864 865 866 869 874 577 580 591

Table 4: **Conservation analysis for the Upf1 set of 18 sequences.**
List of residues resulting from the conservation analysis of the protein family realized with Rate4Site (score of conserved positions ≤ 0.12), ConSurf (conserved residues have rank 8 and 9) and ET methods.

Conservation analysis of Ski2-like - 13 sequences	
**Method**	**List of conserved residues**
Rate4Site	173 174 176 177 178 200 203 239 262 263 265 272 293 294 295 298 421 469 473 474 482 486 500 502 504 506 508 514 537 538 540 541 542 543 544 548
ConSurf	151 154 166 169 170 171 172 173 174 175 176 177 178 180 181 182 183 185 196 197 198 199 200 202 203 204 205 206 207 216 219 222 223 224 225 238 239 241 242 248 253 254 256 260 261 262 263 264 265 267 269 272 273 274 276 277 280 282 283 288 290 292 293 294 295 296 297 298 301 302 303 305 306 408 410 411 412 413 415 416 419 423 468 471 472 473 475 476 477 478 479 483 484 487 488 490 491 494 497 498 499 501 502 503 504 505 506 508 509 510 511 512 513 514 515 516 517 518 519 520 536 539 540 541 542 543 544 545 546 547 550 553 555 556
ET	143 148 150 151 153 154 166 167 168 169 171 172 173 174 175 176 177 178 181 182 183 184 185 195 196 197 198 199 200 201 202 203 204 205 206 207 208 209 210 211 216 218 219 220 221 223 224 225 228 229 232 234 235 236 237 238 239 240 241 242 243 244 245 246 247 249 250 256 259 261 262 263 265 266 267 269 272 273 274 275 276 277 278 280 281 283 284 287 292 293 294 295 296 297 298 302 303 305 306 310 312 318 319 322 323 324 325 326 408 409 410 412 414 415 416 419 420 423 436 451 455 460 467 468 470 471 473 475 476 477 478 479 480 481 482 484 485 487 488 490 491 494 495 497 499 500 501 502 503 504 505 506 508 510 511 512 513 514 515 516 517 518 519 523 525 526 530 535 536 537 539 540 542 543 544 545 546 547 548 550 553 555 556 577 579 581 583 585 587 588 589 591 592 593 595 597 342 348

Table 5: **Conservation analysis for the Ski2-like set of 13 sequences.**

List of residues resulting from the conservation analysis of the protein family realized with Rate4Site (score of conserved positions ≤ 0.12), ConSurf (conserved residues have rank 8 and 9) and ET methods.
