# Supplementary material for: Protein Fragments: Functional and Structural Roles of Their Coevolution Networks
Source: PLoS One. 2012 Nov 5;7(11):e48124. doi: 10.1371/journal.pone.0048124 (PMC3489791; doi:10.1371/journal.pone.0048124)
Supplement: Text S12 — Comparison between BIS analysis based on exceptions and BIS analysis on all hits. (PDF) [file pone.0048124.s014.pdf]

Supporting Information Text S12: comparison between BIS analysis based on exceptions and BIS analysis on all hits

Family	Properties			Block analysis with exceptions						Block analysis with all hits				
	Align length	%id	#seq	#exc	pos cov	# blocks	average block size	min	max	pos cov	# blocks	average block size	min	max
Amyloid	43	0.87	80	0	30	17	2.94	1	14	39	24	3.42	1	17
				1	14	6	2.5	1	7					
				2	17	1	17	17	17					
MUKB	234	0.84	200	0	27	14	2.14	1	7	227	176	2.26	1	11
				1	167	107	2.45	1	11					
				2	69	37	2.24	1	11					
PABD	57	0.82	452	0	37	24	3.25	1	16	53	37	3.08	1	16
				1	21	12	2.83	1	6					
				2	2	1	2	2	2					
UPF1	677	0.58	18	0	41	37	1.27	1	3	399	395	1.13	1	5
				1	67	56	1.196	1	4					
				2	63	56	1.143	1	5					
RECD	642	0.51	6	0	41	33	1.45	1	6	343	316	1.49	1	9
				1	59	51	1.157	1	3					
				2	117	87	1.41	1	7					
UvrD/rep	661	0.53	8	0	34	27	1.59	1	5	346	332	1.33	1	5
				1	52	40	1.375	1	4					
				2	59	54	1.13	1	5					
Rad3	592	0.52	9	0	55	50	1.26	1	3	454	438	1.80	1	36
				1	78	68	1.176	1	4					
				2	136	110	1.25	1	5					
DEAD-box	624	0.61	67	0	44	41	1.22	1	3	430	427	1.07	1	4
				1	65	57	1.21	1	4					
				2	71	66	1.076	1	4					
RecQ	514	0.61	9	0	39	33	1.36	1	4	349	337	1.39	1	20
				1	57	42	1.45	1	5					
				2	103	76	1.37	1	6					
Ski2-like	686	0.51	13	0	48	40	1.625	1	6	426	417	1.16	1	6
				1	78	66	1.21	1	4					
				2	62	54	1.19	1	4					
RigI-like	658	0.47	6	0	68	58	1.43	1	4	451	425	1.41	1	6
				1	65	52	1.27	1	3					
				2	125	104	1.26	1	6					
DEAH-RHA	562	0.65	24	0	45	42	1.21	1	4	409	404	1.17	1	4
				1	92	73	1.47	1	4					
				2	88	74	1.24	1	4					
NS3-NPH-II	479	0.6	11	0	90	83	1.53	1	5	365	353	1.38	1	9
				1	96	84	1.15	1	4					
				2	114	91	1.29	1	6					
Swi2-Snf2	714	0.51	45	0	24	21	1.19	1	3	522	519	1.04	1	5
				1	50	45	1.2	1	5					
				2	87	83	1.05	1	3					

Table 1: **Properties of BIS analysis based on exceptions and based on all hits.** Summary of BIS analysis when realized 1. for different numbers of exceptions (# exc), that is 0, 1 or 2 (columns 5 through 10), and 2. by considering all alignment positions as hits independently on exceptions (last five columns from the right). For each protein family, the alignment length where all gapped positions are excluded (Align length), the sequence identity (%id) and the number of sequences (#seq) are reported. For each analysis, the number of alignment positions retained as hit (pos cov), the number of blocks (#blocks), the average size for a block (average block size), the minimal (min) and maximal (max) size of a block are reported. Notice that for the second analysis (on all hits), the number of positions considered as hits corresponds to all positions in the alignment with the exclusion of those positions that contain more than 60% of gaps.

Family	Properties			Block analysis with exceptions					
	Align length	%id	#seq	#exc	pos cov	# blocks	average block size	min	max
Amyloid	43	0.87	80	0	30	17	2.94	1	14
				0 1	38	23	2.83	1	14
				0 1 2	39	24	3.42	1	17
MUKB	234	0.84	200	0	27	14	2.14	1	7
				0 1	172	121	2.41	1	11
				0 1 2	209	158	2.37	1	11
PABD	57	0.82	452	0	37	24	3.25	1	16
				0 1	52	36	3.11	1	16
				0 1 2	53	37	3.08	1	16
UPF1	677	0.58	18	0	41	37	1.27	1	3
				0 1	97	93	1.26	1	4
				0 1 2	153	149	1.19	1	5
RECD	642	0.51	6	0	41	33	1.45	1	6
				0 1	92	84	1.27	1	6
				0 1 2	180	171	1.36	1	7
UvrD/rep	661	0.53	8	0	34	27	1.59	1	5
				0 1	75	67	1.46	1	5
				0 1 2	129	121	1.31	1	5
Rad3	592	0.52	9	0	55	50	1.34	1	4
				0 1	123	118	1.25	1	4
				0 1 2	234	227	1.26	1	5
DEAD-box	624	0.61	67	0	44	41	1.22	1	3
				0 1	101	98	1.21	1	4
				0 1 2	167	164	1.16	1	4
RecQ	514	0.61	9	0	39	33	1.36	1	4
				0 1	83	75	1.41	1	5
				0 1 2	162	151	1.40	1	6
Ski2-like	686	0.51	13	0	48	40	1.62	1	6
				0 1	115	106	1.37	1	6
				0 1 2	169	160	1.31	1	6
RigI-like	658	0.47	6	0	68	58	1.43	1	4
				0 1	121	110	1.35	1	4
				0 1 2	227	213	1.31	1	6
DEAH-RHA	562	0.65	24	0	45	42	1.21	1	4
				0 1	120	115	1.37	1	4
				0 1 2	194	189	1.32	1	4
NS3-NPH-II	479	0.6	11	0	90	83	1.53	1	5
				0 1	174	167	1.34	1	5
				0 1 2	268	258	1.33	1	6
Swi2-Snf2	714	0.51	45	0	24	21	1.19	1	3
				0 1	69	66	1.20	1	5
				0 1 2	152	149	1.11	1	5

Table 2: **Properties of BIS analysis based on exceptions and based on all hits (cont'd).** Summary of BIS analysis when realized for different numbers of exceptions (# exc), that is 0, 1 or 2, as in Table 1-Text S12. Here, for each protein family we consider a number of positions and of blocks that is cumulated with respect to the number of allowed exceptions. Namely, we count the number of positions/blocks for a number of exceptions d that is 0, ≤ 1 (0 1) or ≤ 2 (0 1 2).
