# Supplementary material for: Protein Fragments: Functional and Structural Roles of Their Coevolution Networks
Source: PLoS One. 2012 Nov 5;7(11):e48124. doi: 10.1371/journal.pone.0048124 (PMC3489791; doi:10.1371/journal.pone.0048124)
Supplement: Text S13 — Instructions for running BIS. (PDF) [file pone.0048124.s015.pdf]

Supporting Information Text S13: instructions for running BIS

1 Instructions for running BIS

The program BIS uses several external tools that should be installed. It requires java6, perl, PhyML v2.4.4 (that creates the phylogenetic tree associated to the set of aligned sequences given as input; it was run with the Blosum62 matrix) (Guindon S, Gascuel O, A simple, fast, and accurate algorithm to estimate large phylogenies by maximum likelihood, *Systematic Biology*, 52(5):696-704, 2003), retree (that transforms trees generated with PhyML in binary trees, if needed; found in the package PHYLIP 3.67 and downloaded at `http://cmgm.stanford.edu/phylip/`) (Felsenstein J, PHYLIP – Phylogeny Inference Package, *Cladistics*, 5:164–166, 1989). It also uses the clustering program CLAG (that clusterizes matrices of coevolution scores; downloaded at `http://www.ihes.fr/∼carbone/data11`) that we include in the package and needs not be installed.

The flowchart illustrating the different steps of BIS method highlights two alternative pathways of co-evolution analysis, one based on positions (in red) and the other based on blocks (in blue).

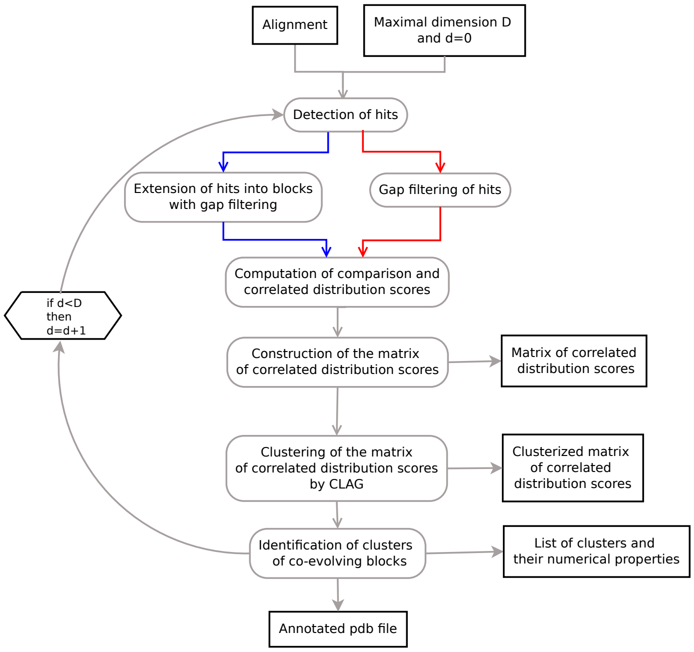

The program BIS has been designed to detect co-evolution starting from an alignment file, say PF00000, and it is split into two steps:

1. the calculation of co-evolution of pairs of blocks/positions and the generation of networks and clusters
2. the visualization of clusters in a matrix form, and the creation of an annotated pdb file with clusters information.

The first part of the algorithm needs as input the aligned sequences filename (with extension `_full.txt`) and an upper limit D on the number of exceptions allowed. The first sequence in the alignment is considered to be the reference sequence.

Example. Let us consider the file `PF00000_full.txt`, containing 4 aligned sequences:

name1 FNKEQQNAFYEILNMSNLNEEQRNGFIQSLKDDPSQSAN
name2 FNKEQQNAFYEILNMSNLNEEQRNGFIQSLKDDPSQSAN
name3 FNKEQQNAFYEILNMSNLNEEQRNGFIQSLKDDPSQSAN
name4 FNKEQQNAFYEILNMSNLNEEQRNGFIQSLKDDPSQSAN

and suppose it to be located in the path `DATA-block/PABD/452/`. To run BIS, the command line is:

./exe.pl -f=DATA-block/PABD/452/ -d=4 -h=y -e=/UserName/TOOLBIS-2 -s=/UserName/Setup/ -o=macOS

where the arguments are:
f: path where to find the file containing the alignment having extension `_full.txt`;
d: number of exceptions allowed;
h: coevolution analysis based on blocks - y or n;
e: BIS scripts localization;
s: setup folder localisation for phylip and phyml;
o: os either linux or macOS.

BIS realizes coevolution analysis based on the physico-chemical properties of the amino-acids, instead of considering the amino-acids themselves. This can be done by including in the command line the argument "-pc=y".

BIS realizes coevolution analysis by considering all positions as hits, instead of considering the subset defined by the number of exceptions d. This can be done by including in the command line the argument "-a=y". If this command is not present, then the argument d is used instead.

BIS generates the associated phylogenetic tree, identifies hits for every d where $0 \leq d \leq 4$, extends them to generate blocks and computes co-evolving scores among these blocks in order to determine clusters of co-evolving blocks. Clusterization is realized by invoking the program CLAG. The results are placed in the directory Results, in the path set with the option -f.

To run BIS on alignment positions, instead of blocks, we give the argument h the value n:

./exe.pl -f=DATA-block/PABD/452/ -d=4 -h=n -e=/UserName/TOOLBIS-2 -s=/UserName/Setup/ -o=macOS

BIS generates the associated phylogenetic tree, identifies hits for every d where $0 \leq d \leq 4$, filters gap positions and computes co-evolving scores among these positions in order to determine clusters of co-evolving positions. The results will be placed in the directory Results in the path set with the option -f.

The second part of the algorithm identifies co-evolving positions on the reference sequence and visualizes them on a matrix. The matrix identifiers (for blocks or residues) can either be alignment positions or pdb positions. When the user mentions the pdb file name then the output result will be given with pdb identifiers. The visualization program takes as input the alignment, the number of exceptions D and the pdb file (if

it exists; it should be placed in the same folder as the alignment file indicated by the user with the option -f) and it provides a set of figures representing clustered blocks (or residues) for each number of exceptions $d \leq D$. All matrices for d and d^+ are generated. No figure is generated when the option -a is used. Notice that BIS also annotates the temperature column of the pdb file by affecting the same colors to residues belonging to the same cluster. All files are placed on the directory Results, automatically created in the path defined by the user using the option -f.

Example. To run the program using the command line, where the PDB is 1BDD, the chain is A and the name of the associated PDB sequence in the alignment is nameRef, write:

./exe-RCommand.pl -f=DATA-block/PABD/452 -d=4 -e=/UserName/TOOLBIS-2 -p=1BDD -c=A -n=nameRef

where the arguments are:
f: path;
d: number of exceptions allowed;
e: BIS scripts localization;
p: pdb;
c: chain;
n: nameRef (it is the reference sequence name, used to realize the correspondance between pdb and alignment positions).

The optional command "-pc=y", for coevolution analysis based on the physico-chemical properties of the amino-acids, can be added to the list of mandatory arguments. The same holds for the optional command "-a=y", for coevolution analysis considering all positions as hits.

1.1 BIS Parameters

A parameter for the number of exceptions. Each position of an alignment has a number of exceptions associated to it. It corresponds to the number d of residues with no repetitions along the alignment column. We say that a *hit* (that is, a position) has d *exceptions*, for some d. BIS evaluates the correlations between blocks/positions with d (d-environment) exceptions and between blocks/positions with at most d (d^+-environment) exceptions.

Notice that the parameter Δ, needed in the clustering algorithm CLAG, could be also used to further decipher the structure of the co-evolution signal. The modulation of both parameters d and Δ allows the user to investigate a fine structure of the signal.

1.2 BIS output files

BIS first step outputs several files and locates them in the directory Results, automatically created in the path specified by the user with the parameter -f. The first list concerns the phylogenetic tree evaluation and the definition of blocks and environments:

PF00000-input.txt: it contains the phylogenetic tree
PF00000-output.txt: it contains the binary tree generated by retree
PF00000-outHit.txt: it describes hits for all number of exceptions d, and there is a line for each residue it is composed to. Each line is of the form X,Y,Z, where X is a residue associated to the hit, Y is the alignment position and Z is a boolean value saying whether the residue is (1) or not (0) an exception (that is, it has only one occurrence). Blank lines separate the treatment of different numbers of exceptions.
PF00000-outGroupsMotifs.txt: it describes all pseudo-blocks derived from hits

PF00000-outBlocksMotifsDIM.txt: it describes blocks forming the d-environment
PF00000-outBlocksMotifs.txt: it describes blocks forming the d^+-environment
PF0000-familySeq-Ordered.txt: it contains the protein sequences reshuffled according to the PHYML tree
PF0000-HitOfBlocks.txt: it enumerates the hit that was extended to generate each block. Notice that a block might be generated by several hits. Blank lines separate lists of blocks obtained by treating different numbers of exceptions.
PF0000-ENVIRONMENT-DM-MatrixD.txt: it lists blocks composing the d environment. The positions enumerated here might be either pdb positions or alignment positions. Each line has the form X:Y:Z where Z is a block in the alignment, X is the block Z but rewritten with pdb positions if the pdb is available, otherwise with alignment positions, Y is the block Z but rewritten with alignment positions.
PF0000-ENVIRONMENT-DM-MatrixDD.txt: similarly as above, it lists blocks forming the d^+-environment.

Another list of files is issued from the evaluation of S^T_{corr}. For instance, for $d = \mathbf{N}$ we have:

PF00000-SAM.txt: it is the list of all subtrees of the binary tree. Each line contains the description of a subtree, and it is of the form List,X,Y,Z, where List is the list of the leaves of the tree indexing the sequences in the alignment and ordered accordingly to the tree, X is the distance from the root (data not used), Y is the level in the tree (data not used), and Z is a subtree identifier (obtained by recursively reading the main tree from the root to the leaves following a prefix order).
PF00000-PR-output.txt: it contains information associated to the calculation of the score S_{comp}. Namely, the sum over all elements in $\mathcal{B}_1$ is computed. Value computed for each word in $\mathcal{B}_1$ are recorded.
PF00000-PR-SCORING-output-D**N**.txt: it contains S^T_{corr} values for every pair of blocks.

The files associated to the clusterisation for d and d^+-environments at a given value Δ coming from CLAG's clustering, say $\Delta = 0.\mathbf{M}$, are:

PF00000-CLUSTERFILE-**M**D.txt: each line is a description of a different cluster and its corresponding scores. Only clusters with positive scores are reported. Analysis done for d.
PF00000-CLUSTERFILE-**M**DD.txt: as above, for d^+.

where the CLAG parameter Δ is set by default to $0.05, 0.10, 0.20, 0.40$. Each line in the files is a description of a distinguished cluster and their corresponding positive scores (clusters with negative scores are not reported).

The implementation of CLAG included in BIS generates several figures (when the option -a is not used):

PF00000-Matrix-d-D.pdf: unclustered matrix for d
PF00000-Matrix-d-DD.pdf: unclustered matrix for d^+
PF00000-Matrix-d-Clusterized-M-D.pdf: the clustered matrix for all scores, for d
PF00000-Matrix-d-Clusterized-M-DD.pdf: the clustered matrix for all scores, for d^+
PF00000-Matrix-d-Clusterized-M-Scores1-D.pdf: the clustered matrix for environmental scores = 1 (and symmetric scores = 1), for d. The generation of this matrix can be dropped off on a comment line in the code. This option has been imposed because, at times, the generated files are too large and the user might want to avoid their generation.
PF00000-Matrix-d-Clusterized-M-Scores1-DD.pdf: as above for d^+.

Matrices are indexed by alignment positions if the PDB is not available, by PDB positions otherwise. All these files are found in the directory 'Results'.

Whenever the PDB file is given, a folder might be created where, for each cluster, an annotated pdb file (on the "temperature" column) is provided. If one wants to activate this feature, she/he needs to move line 112-128 of the exe-RCommandPDB.pl (called by exe-RCommand.pl) just before the code line "exit 0;" at

line 100 of the same file. Notice that these files are usually large.

Note that the files beginning with "PF00000-PR-SCORING-output" contain the matrix given to R for the generation of the corresponding pdf files. The commands given to R are contained in the files beginning with "PF00000_R_COMMAND". All files generated by R and neato are in pdf format.

Warning: at times, the tree issued by the algorithm retree is not binary at the root and it should be forced to be binary, possibly by hand. The criteria followed to root the tree are described in Methods.

1.3 BIS coevolution analysis based on all alignment positions as hits: option -a

BIS allows the user to work with blocks generated by all alignment positions instead of a subset dependent on exceptions (with the option -a). In this case, all alignment positions are hits of blocks (strictly speaking, the length of the alignment is an upper bound to the number of generated blocks because positions with more than 60% of gaps are not considered as hits). For long proteins, this means that BIS should handle a very large number of blocks and that its computational time will be affected since, this latter, is quadratic on the number of defined blocks (see next section). For AATPase subfamilies for instance, the analysis based on all positions as hits has to handle from 310 up to 520 blocks depending on the subfamily, while the analysis based on exceptions done above with $d \leq 1$, handled less than 100 blocks (see SI Table 82). It is important to say that BIS coevolution analysis based on exceptions or based on all alignment positions as hits gives the same result when the characterized blocks are the same. This is the case for the Amyloid family, for instance, where the blocks identified for $d \leq 2$ coincide with the blocks generated by all positions (see SI Tables 82-83).

Note that when an alignment is constituted by a few sequences, say N, as it can be the case for the pool of sequences we handle, it might be that certain positions are occupied by N distinct letters. This would imply that the block associated to this position is the full alignment. BIS does not evaluate such extreme situations (of no interest for coevolution) and proposes this option to allow the user experiment the tool with looser parameters. This point is addressed in the discussion.

1.4 BIS time of execution.

Let N be the number of sequences and l be the length of the alignment. The time of execution for BIS (where clustering is not taken into account) depends on two major parameters: the number of blocks/positions with d (d^+) exceptions which is bound by l and the number of subtrees in the distance tree T associated to the alignment which is bound by N. Based on the fact that the coevolving score is computed recursively for each pair of blocks/positions and for each subtree of the phylogenetic tree, and that it is based on the computation of the comparison score that is dependent on the number of sequences N, the computational time of the algorithm is $\mathcal{O}(N^2 \cdot l^2)$. The algorithm is run for several d and d^+. Notice that by increasing d^+ we increase the number of blocks to be considered in the analysis. This is not necessarily the case when d is increased.
