# Supplementary material for: Protein Fragments: Functional and Structural Roles of Their Coevolution Networks
Source: PLoS One. 2012 Nov 5;7(11):e48124. doi: 10.1371/journal.pone.0048124 (PMC3489791; doi:10.1371/journal.pone.0048124)
Supplement: Text S14 — Systems of coevolution and conservation analysis used for comparison, sets of aligned sequences, and clustering. (PDF) [file pone.0048124.s016.pdf]

Supporting Information Text S14: Systems of coevolution and conservation analysis used for comparison, sets of aligned sequences, and clustering

1 Comparison of BIS with other systems of coevolution analysis

Comparison with other systems. BIS was run on blocks and on positions and comparison with several perturbation-based functions was realized. The systems are:

A. Statistical Coupling Analysis (SCA) (Lockless SW, Ranganathan R, Evolutionary conserved pathways of energetic connectivity in protein families. *Science*, 286:295–299, 1999): we used the SCA implementation in COEVOLUTION (Yip KY, Patel P, Kim PM, Engelman DM, McDermott D, Gerstein M, An integrated system for studying residue coevolution in proteins. *Bioinformatics*, 24: 290-292, 2008) downloaded at http://coevolution.gersteinlab.org/coevolution/. Since not all the algorithmic details were given in the SCA reference sources, the implementation follows a number of designed choices. Two functions (DB and TM) were proposed to compute a "symmetric score" in COEVOLUTION. We compared to both of them. It is important to notice that the implementation we used applies to all alignment positions while the SCA analysis is supposed to apply only to positions that are sufficiently divergent. Very conserved positions are usually filtered out by SCA analysis contrary to COEVOLUTION that successfully detects patterns like the Walker-A motif, even though very conserved (see SI Tables 3-4).

B. Explicit Likelihood of Subset Variation (ELSC) (Dekker JP, Fodor A, Aldrich RW, Yellen G, A perturbation-based method for calculating explicit likelihood of evolutionary co-variance in multiple sequence alignments. *Bioinformatics*, 20: 1565–1572, 2004): its implementation is available through COEVOLUTION.

C. Mutual Information (MI) (Gloor GB, Martin LC, Wahl LM, Dunn SD, Mutual Information in protein multiple sequence alignments reveals two classes of coevolving positions. *Biochemistry*, 44:7156-7165, 2005) can be viewed as a generalized perturbation method: it considers all residues occupying a pair of positions and combines them by a weighted average according to their frequencies. We used the standard definition of MI and the implementation of COEVOLUTION.

D. Maximal SubTree method (MST) (Baussand J, Carbone A, A combinatorial approach to detect co-evolved amino-acid networks in protein families with variable divergence. *PLoS Computational Biology*, 5(9):e1000488, 2009): downloaded at http://www.ihes.fr/∼carbone/data7/MaxSubTree.tgz and run with

default values for its parameters.

E. Continuous-Time Markov Process (CTMP) (Yeang C-H, Haussler D, Detecting coevolution in and among proteins domains. *PLoS Comp. Biol.*, 3:2122–2134, 2007): found at http://www.stat.sinica.edu.tw/chyeang/

Comparative tests run on different datasets of sequences. For the MukB and Protein A families, we tested BIS and all systems considered for comparison on four datasets of sequences. Given a protein family, we considered the full dataset of PFAM sequences (v23), and its restriction to homologous sequences that are not very divergent from the rest of the set (this filtering was realized by manually eliminating from the associated distance tree all isolated long branches). Since some of the sequences in these two datasets might be 100% identical, for each of the two sets we also run the analysis on the respective subsets admitting at most 98% of sequence identity (filtering was done with COEVOLUTION). For the MukB family, we consider datasets of 205, 200, 54 and 49 sequences; for Protein A, datasets contain 490, 452, 28 and 20 sequences. For the Amyloid beta peptide we considered two datasets of 80 and 16 sequences by filtering out almost identical sequences. For the SF1 and SF2 AATPase subfamilies, we considered the alignments used in (Fairman-Williams et al. 2010).

Comparative tests based on an automatic clustering. Clustering of correlation scores matrices associated to the different methods has been done with the automatic clustering algorithm CLAG, run with parameter $\Delta = 0.05$. The usage of this clustering algorithm allowed to compare the systems. We need to recall here that no automatic clustering was available before CLAG for coevolution score matrices, and that this is the first systematic comparison on the prediction performance of these systems. BIS clusters extracted and reported in the SI Tables have environmental score = 1. For other systems, we allowed symmetric and environmental scores to be > 0. Clusters strength is measured by CLAG symmetricity score.

An overview of methodologies comparison on all protein families is found in SI Table 0 where we rapidly describe the outcomes of each system of coevolution analysis, for each protein family. Predictions of all methods in all datasets are reported in Text S10.

**Predictions by methods of coevolution analysis**									
**Family**	**Seq**	**%Identity**	**BIS**	**SCA-DB**	**SCA-TM**	**ELSC**	**MI**	**CTMP**	**MST**
Walker-A	205	0.81	4	-	-	6	5	-	6
in MukB	200	0.84	8	8	8	13	-	-	-
	54	0.70	4	-	-	-	5	-	6
	49	0.77	8	8	8	13	-	-	-
Protein A-	490	0.75	5	16	11	2	35	-	9
B domain	452	0.82	30	29	28	30	14	-	17
	28	0.60	5	5	5	7	32	-	5
	20	0.71	30	-	-	30	2	-	13
Amyloid	80	0.87	27	-	-	28	25	-	16
	16	0.77	24	-	-	25	5	-	21
SF1: Upf1	18	0.58	26	8	5	22	-	-	35
SF1: RecD	6	0.51	73	12	10	87	-	-	63
SF1: UvrD/Rep	8	0.53	38	18	16	65	-	-	59
SF2: Rad3	9	0.52	62	22	7	25	-	-	110
SF2: DEAD-box	67	0.61	16	4	4	-	-	-	17
SF2: RecQ	9	0.61	42	47	47	35	-	-	81
SF2: Ski2-like	13	0.51	35	6	6	70	-	-	53
SF2: RigI-like	6	0.47	104	8	8	70	-	-	207
SF2: DEAH-RHA	24	0.65	43	17	13	15	-	-	55
SF2: NS3/NPH-II	11	0.60	101	2	2	2	-	-	132
SF2: Swi2/Snf2	45	0.51	15	-	-	19	-	-	20

Table 0. **Overview of methodologies comparison on all protein families.** Protein families (first column), number of sequences considered in the alignment (second column), %Identity (third column) and number of co-evolving positions predicted by different methodologies (fourth to sixth columns). BIS is run on blocks. CLAG clustering is done asking for

symmetric and environmental scores to be > 0 for SCA-DB, SCA-TM, ELSC, MI, CTMP, MST, and for scores $= 1$ for BIS; its parameter Δ is set to 0.05, for all methods. The symbol - is used to indicate that a methodology did not provide any coevolving cluster for a given dataset. Methods performances are reported in Text S10.

2 Comparison of BIS with other systems of conservation analysis

Comparison with other systems. Comparison with systems for conservation analysis was done on online tools:

A. ET Viewer 2.0 was accessed at `http://mammoth.bcm.tmc.edu/` (Lichtarge O, Bourne HR, Cohen FE, An evolutionary trace method defines binding surfaces common to protein families. *J Mol Biol*, 257: 342-358, 1996).

B. Consurf was accessed at `http://consurf.tau.ac.il/` and conserved residues were selected by fixing the rank at 8 and 9 (Armon A, Graur D, Ben-Tal N, ConSurf: an algorithmic tool for the identification of functional regions in proteins by surface mapping of phylogenetic information. *J Mol Biol*, 307: 447-463, 2001).

C. Rate4Site was run at `http://www.tau.ac.il/ itaymay/cp/rate4site.html` with score for conserved positions ≤ 0.12 (Mayrose I, Graur D, Ben-Tal N, Pupko T, Comparison of site-specific rate-inference methods: Bayesian methods are superior. *Mol Biol Evol* 21: 1781-1791, 2004).

Their performance is reported in Text S10.
