# Supplementary material for: Protein Fragments: Functional and Structural Roles of Their Coevolution Networks
Source: PLoS One. 2012 Nov 5;7(11):e48124. doi: 10.1371/journal.pone.0048124 (PMC3489791; doi:10.1371/journal.pone.0048124)
Supplement: Text S15 — Mapping between PDB positions and alignment positions. (PDF) [file pone.0048124.s017.pdf]

Supporting Information Text S15: mapping between PDB positions and alignment positions

Matrix rank	PDB	Alignment
1	1	3
2	4	6
3	4-6	6-8
4	6-7	8-9
5	6	8
6	9	11
7	11	13
8	12	14
9	14-20	16-22
10	22-23	24-25
11	26	28
12	34-37	36-39
13	39-42	41-44
14	144	146

Table 1: **MukB protein: BIS environment for $d = 0$ on the dataset of 200 sequences.** List of blocks defined for $d = 0$ over which the correlated distribution score matrix analysis is computed. Blocks are identified by the starting and ending positions if they are composed by several amino-acids, or by one single position otherwise. Positions are given for the PDB file (second column) and for the PFAM sequence alignment (third column). The matrix rank corresponds to the line in the correlated distribution score matrix starting from the bottom up to the top in Fig. 2B in the main text.

Matrix rank	PDB	Alignment	Matrix rank	PDB	Alignment	Matrix rank	PDB	Alignment
1	2	4	37	101	103	73	170	172
2	6-9	8-11	38	104	106	74	171	173
3	10-11	12/13/11	39	105	107	75	173	175
4	13-20	15-22	40	107-108	109-110	76	174-175	176-177
5	14-23	250	41	108-109	109-111	77	175	177
6	22-24	24-26	42	108	110	78	175-176	177-178
7	25-26	27-28	43	111-117	113-119	79	178-179	180-181
8	28-33	30-35	44	111-113	113-115	80	179-180	181-182
9	29-33	31-35	45	112-113	114-115	81	179	181
10	34-42	36-44	46	115-116	117-118	82	181-182	183-184
11	44-45	46-47	47	115-117	117-119	83	182	184
12	48	50	48	118	120	84	184	186
13	49	51	49	119	121	85	185	187
14	50	52	50	120-121	122-123	86	186	188
15	51-54	55-56	51	121	123	87	187	189
16	52-55	54-57	52	125	127	88	189-190	191-192
17	52-54	54-56	53	126	128	89	189	191
18	56-58	58-60	54	132-133	134-135	90	191	193
19	57-58	59-60	55	133	135	91	193-194	195-196
20	57-67	59-69	56	136	138	92	197-198	199-200
21	60-68	62-70	57	137	139	93	198-200	200-202
22	60-67	62-69	58	141	143	94	198	200
23	69	71	59	145	147	95	200	202
24	71-74	73-76	60	146	148	96	202-210	204-212
25	72-74	74-76	61	147	149	97	212-214	214-216
26	76-77	78-79	62	148	150	98	213-214	215-216
27	76-81	78-83	63	149	151	99	213	215
28	79-82	81-84	64	150	152	100	215-218	217-220
29	79-81	81-83	65	152	154	101	216-223	218-225
30	84	86	66	153	155	102	216-218	218-220
31	86-87	88-89	67	157	159	103	220-223	222-225
32	91	93	68	158	160	104	220-225	222-227
33	94-95	96-97	69	160	162	105	225	227
34	98-99	100-101	70	162	164	106	226	228
35	98-100	100-102	71	163	165	107	227	229
36	99	101	72	168	170			

Table 2: **MukB protein: BIS environment for $d = 1$ on the dataset of 200 sequences.** List of blocks defined for $d = 1$ over which the correlated distribution score matrix is computed. Blocks are identified by the starting and ending positions if they are composed by several amino-acids, or by one single position otherwise. Positions are given for the PDB file (second column) and for the PFAM sequence alignment (third column).

Amino acid	Alignment	PDB
Q	34	475
A	37	478
G	70	492
G	73	495
G	75	497
K	76	498
L	125	518
R	168	543
T	213	616
E	257	637
G	315	661
D	316	662
L	417	698
R	422	703
Y	436	717
N	513	766
Y	554	796
Q	557	799
Q	708	830
G	709	831
G	786	851
R	801	865

Table 3: **AATPase Upf1: mapping between structural and sequence positions of co-evolving amino-acids.**

Co-evolving residues clustered by BIS analysis of Upf1 are described through structural positions (used in Fig. 5A in the main text; pdb structure `2gjk`) and alignment positions (used in Fig. 5B in the main text). Notice that alignment positions refer to the alignment given in (Fairman-Williams et al. 2010).

Amyloid 12 sequences		
Res	Al pos	PDB
F	4	4
R	5	5
H	6	6
D	7	7
S	8	8
G	9	9
Y	10	10
E	11	11
V	12	12
H	13	13
H	14	14
Q	15	15
K	16	16
L	17	17
V	18	18
F	19	19
F	20	20
A	21	21
E	22	22
D	23	23
V	24	24
G	25	25
S	26	26
N	27	27
K	28	28
G	29	29
A	30	30
I	31	31
I	32	32
G	33	33
L	34	34
M	35	35
V	36	36
G	37	37
G	38	38
V	39	39
V	40	40
I	41	41
A	42	42

Table 4: **Alignment vs PDB residue mapping for the Amyloid set of 12 sequences.** Correspondence between alignment positions (Al pos) and PDB positions (PDB). The residue (Res) corresponding to a given position is also reported.

**Amyloid 80 sequences**		
Res	Al pos	PDB
F	4	4
R	5	5
H	6	6
D	7	7
S	8	8
G	9	9
Y	10	10
E	11	11
V	12	12
H	13	13
H	14	14
Q	15	15
K	16	16
L	17	17
V	18	18
F	19	19
F	20	20
A	21	21
E	22	22
D	23	23
V	24	24
G	25	25
S	26	26
N	27	27
K	28	28
G	29	29
A	30	30
I	31	31
I	32	32
G	33	33
L	34	34
M	35	35
V	36	36
G	37	37
G	38	38
V	39	39
V	40	40
I	41	41
A	42	42

Table 5: **Alignment vs PDB residue mapping for the Amyloid set of 80 sequences.**
See legend Table 4-Text S15.

Protein A-B domain 490 sequences		
Res	Al pos	PDB
A	5	2
D	7	3
N	8	4
K	9	5
F	10	6
N	11	7
K	12	8
E	13	9
Q	14	10
Q	15	11
N	16	12
A	17	13
F	18	14
Y	19	15
E	20	16
I	21	17
L	22	18
H	23	19
L	24	20
P	25	21
N	26	22
L	27	23
N	28	24
E	29	25
E	30	26
Q	31	27
R	32	28
N	33	29
G	34	30
F	35	31
I	36	32
Q	37	33
S	38	34
L	39	35
K	40	36
D	41	37
D	42	38
P	43	39
S	44	40
Q	45	41
S	46	42
A	47	43
N	48	44
L	49	45
L	50	46
A	51	47
E	52	48
A	53	49
K	54	50
K	55	51
L	56	52
N	57	53
D	58	54
A	59	55

Table 6: **Alignment vs PDB residue mapping for the Protein A-B domain set of 490 sequences.** See legend Table 4-Text S15.

Protein A-B domain 452 sequences		
Res	Al pos	PDB
A	1	2
D	3	3
N	4	4
K	5	5
F	6	6
N	7	7
K	8	8
E	9	9
Q	10	10
Q	11	11
N	12	12
A	13	13
F	14	14
Y	15	15
E	16	16
I	17	17
L	18	18
H	19	19
L	20	20
P	21	21
N	22	22
L	23	23
N	24	24
E	25	25
E	26	26
Q	27	27
R	28	28
N	29	29
G	30	30
F	31	31
I	32	32
Q	33	33
S	34	34
L	35	35
K	36	36
D	37	37
D	38	38
P	39	39
S	40	40
Q	41	41
S	42	42
A	43	43
N	44	44
L	45	45
L	46	46
A	47	47
E	48	48
A	49	49
K	50	50
K	51	51
L	52	52
N	53	53
D	54	54
A	55	55

Table 7: **Alignment vs PDB residue mapping for the Protein A-B domain set of 452 sequences.**
See legend Table 4-Text S15.

Protein A-B domain 28 sequences		
Res	Al pos	PDB
A	5	2
D	7	3
N	8	4
K	9	5
F	10	6
N	11	7
K	12	8
E	13	9
Q	14	10
Q	15	11
N	16	12
A	17	13
F	18	14
Y	19	15
E	20	16
I	21	17
L	22	18
H	23	19
L	24	20
P	25	21
N	26	22
L	27	23
T	28	24
E	29	25
E	30	26
Q	31	27
R	32	28
N	33	29
G	34	30
F	35	31
I	36	32
Q	37	33
S	38	34
L	39	35
K	40	36
D	41	37
D	42	38
P	43	39
S	44	40
V	45	41
S	46	42
K	47	43
E	48	44
I	49	45
L	50	46
A	51	47
E	52	48
A	53	49
K	54	50
K	55	51
L	56	52
N	57	53
D	58	54
A	59	55

Table 8: **Alignment vs PDB residue mapping for the Protein A-B domain set of 28 sequences.** See legend Table 4-Text S15.

Protein A-B domain 20 sequences		
Res	Al pos	PDB
A	1	2
D	3	3
N	4	4
K	5	5
F	6	6
N	7	7
K	8	8
E	9	9
Q	10	10
Q	11	11
N	12	12
A	13	13
F	14	14
Y	15	15
E	16	16
I	17	17
L	18	18
H	19	19
L	20	20
P	21	21
N	22	22
L	23	23
T	24	24
E	25	25
E	26	26
Q	27	27
R	28	28
N	29	29
G	30	30
F	31	31
I	32	32
Q	33	33
S	34	34
L	35	35
K	36	36
D	37	37
D	38	38
P	39	39
S	40	40
V	41	41
S	42	42
K	43	43
E	44	44
I	45	45
L	46	46
A	47	47
E	48	48
A	49	49
K	50	50
K	51	51
L	52	52
N	53	53
D	54	54
A	55	55

Table 9: **Alignment vs PDB residue mapping for the Protein A-B domain set of 20 sequences.** See legend Table 4-Text 15.

MukB - 205 sequences																	
Res	Al pos	PDB	Res	Al pos	PDB	Res	Al pos	PDB	Res	Al pos	PDB	Res	Al pos	PDB	Res	Al pos	PDB
M	7	1	S	80	41	L	120	81	F	162	121	E	203	161	I	243	201
I	8	2	T	81	42	K	121	82	A	163	122	G	204	162	E	244	202
E	9	3	T	82	43	A	122	83	I	164	123	V	205	163	A	245	203
R	10	4	M	83	44	G	124	84	Q	165	124	Q	206	164	S	246	204
G	11	5	A	84	45	V	126	85	G	166	125	F	207	165	L	247	205
K	12	6	A	85	46	C	127	86	L	167	126	K	208	166	Y	248	206
F	13	7	F	86	47	Y	128	87	P	168	127	Q	209	167	G	249	207
R	14	8	V	87	48	S	129	88	M	169	128	F	210	168	G	250	208
S	15	9	T	88	49	M	130	89	S	170	129	N	211	169	I	251	209
L	16	10	A	89	50	L	131	90	V	171	130	S	212	170	S	252	210
T	17	11	L	90	51	D	132	91	Q	172	131	I	213	171	S	253	211
L	18	12	I	91	52	T	133	92	P	173	132	T	214	172	A	254	212
I	19	13	P	92	53	I	134	93	T	174	133	D	215	173	I	255	213
N	20	14	D	93	54	N	135	94	Q	175	134	Y	216	174	T	256	214
W	21	15	L	94	55	S	136	95	L	176	135	H	217	175	R	257	215
N	22	16	T	95	56	H	137	96	V	177	136	S	218	176	S	258	216
G	23	17	L	96	57	H	138	97	T	178	137	L	219	177	L	259	217
F	24	18	L	97	58	Q	139	98	E	179	138	M	220	178	R	260	218
F	25	19	H	98	59	R	140	99	T	180	139	F	221	179	D	261	219
A	26	20	F	99	60	V	141	100	L	181	140	D	222	180	Y	262	220
R	27	21	R	100	61	V	142	101	N	182	141	L	223	181	L	263	221
T	28	22	N	101	62	V	143	102	E	183	142	G	224	182	L	264	222
F	29	23	T	102	63	G	144	103	R	184	143	I	225	183	P	265	223
D	30	24	T	103	64	V	145	104	Q	185	144	I	226	184	E	266	224
L	31	25	E	104	65	R	146	105	A	186	145	A	227	185	N	267	225
D	32	26	A	105	66	L	147	106	R	187	146	R	228	186	S	268	226
E	33	27	G	106	67	Q	148	107	V	188	147	R	229	187	G	269	227
L	45	28	A	107	68	Q	149	108	L	189	148	L	230	188			
V	60	29	T	108	69	V	150	109	P	190	149	R	231	189			
T	61	30	S	109	70	A	151	110	L	191	150	S	232	190			
T	70	31	G	110	71	G	152	111	N	193	151	A	233	191			
L	71	32	S	111	72	R	153	112	E	194	152	S	234	192			
S	72	33	R	112	73	D	154	113	L	195	153	D	235	193			
G	73	34	D	113	74	R	155	114	K	196	154	R	236	194			
G	74	35	K	114	75	K	156	115	D	197	155	S	237	195			
N	75	36	G	115	76	V	157	116	K	198	156	K	238	196			
G	76	37	L	116	77	D	158	117	L	199	157	F	239	197			
A	77	38	H	117	78	I	159	118	E	200	158	Y	240	198			
G	78	39	G	118	79	K	160	119	A	201	159	R	241	199			
K	79	40	K	119	80	P	161	120	M	202	160	L	242	200			

Table 10: **Alignment vs PDB residue mapping for the MukB domain set of 205 sequences.** See legend Table 4-Text S15.

MukB - 200 sequences																	
Res	Al pos	PDB	Res	Al pos	PDB	Res	Al pos	PDB	Res	Al pos	PDB	Res	Al pos	PDB	Res	Al pos	PDB
M	3	1	S	43	41	L	83	81	F	123	121	E	163	161	I	203	201
I	4	2	T	44	42	K	84	82	A	124	122	G	164	162	E	204	202
E	5	3	T	45	43	A	85	83	I	125	123	V	165	163	A	205	203
R	6	4	M	46	44	G	86	84	Q	126	124	Q	166	164	S	206	204
G	7	5	A	47	45	V	87	85	G	127	125	F	167	165	L	207	205
K	8	6	A	48	46	C	88	86	L	128	126	K	168	166	Y	208	206
F	9	7	F	49	47	Y	89	87	P	129	127	Q	169	167	G	209	207
R	10	8	V	50	48	S	90	88	M	130	128	F	170	168	G	210	208
S	11	9	T	51	49	M	91	89	S	131	129	N	171	169	I	211	209
L	12	10	A	52	50	L	92	90	V	132	130	S	172	170	S	212	210
T	13	11	L	53	51	D	93	91	Q	133	131	I	173	171	S	213	211
L	14	12	I	54	52	T	94	92	P	134	132	T	174	172	A	214	212
I	15	13	P	55	53	I	95	93	T	135	133	D	175	173	I	215	213
N	16	14	D	56	54	N	96	94	Q	136	134	Y	176	174	T	216	214
W	17	15	L	57	55	S	97	95	L	137	135	H	177	175	R	217	215
N	18	16	T	58	56	H	98	96	V	138	136	S	178	176	S	218	216
G	19	17	L	59	57	H	99	97	T	139	137	L	179	177	L	219	217
F	20	18	L	60	58	Q	100	98	E	140	138	M	180	178	R	220	218
F	21	19	H	61	59	R	101	99	T	141	139	F	181	179	D	221	219
A	22	20	F	62	60	V	102	100	L	142	140	D	182	180	Y	222	220
R	23	21	R	63	61	V	103	101	N	143	141	L	183	181	L	223	221
T	24	22	N	64	62	V	104	102	E	144	142	G	184	182	L	224	222
F	25	23	T	65	63	G	105	103	R	145	143	I	185	183	P	225	223
D	26	24	T	66	64	V	106	104	Q	146	144	I	186	184	E	226	224
L	27	25	E	67	65	R	107	105	A	147	145	A	187	185	N	227	225
D	28	26	A	68	66	L	108	106	R	148	146	R	188	186	S	228	226
E	29	27	G	69	67	Q	109	107	V	149	147	R	189	187	G	229	227
L	30	28	A	70	68	Q	110	108	L	150	148	L	190	188			
V	31	29	T	71	69	V	111	109	P	151	149	R	191	189			
T	32	30	S	72	70	A	112	110	L	152	150	S	192	190			
T	33	31	G	73	71	G	113	111	N	153	151	A	193	191			
L	34	32	S	74	72	R	114	112	E	154	152	S	194	192			
S	35	33	R	75	73	D	115	113	L	155	153	D	195	193			
G	36	34	D	76	74	R	116	114	K	156	154	R	196	194			
G	37	35	K	77	75	K	117	115	D	157	155	S	197	195			
N	38	36	G	78	76	V	118	116	K	158	156	K	198	196			
G	39	37	L	79	77	D	119	117	L	159	157	F	199	197			
A	40	38	H	80	78	I	120	118	E	160	158	Y	200	198			
G	41	39	G	81	79	K	121	119	A	161	159	R	201	199			
K	42	40	K	82	80	P	122	120	M	162	160	L	202	200			

Table 11: **Alignment vs PDB residue mapping for the MukB domain set of 200 sequences.** See legend Table 4-Text S15.

MukB 54 sequences																	
Res	Al pos	PDB	Res	Al pos	PDB	Res	Al pos	PDB	Res	Al pos	PDB	Res	Al pos	PDB	Res	Al pos	PDB
M	7	1	S	80	41	L	120	81	F	162	121	E	203	161	I	243	201
I	8	2	T	81	42	R	121	82	M	163	122	G	204	162	E	244	202
E	9	3	T	82	43	P	122	83	I	164	123	L	205	163	A	245	203
R	10	4	M	83	44	G	124	84	Q	165	124	H	206	164	S	246	204
G	11	5	A	84	45	V	126	85	G	166	125	F	207	165	L	247	205
K	12	6	A	85	46	C	127	86	V	167	126	K	208	166	Y	248	206
F	13	7	F	86	47	Y	128	87	P	168	127	Q	209	167	G	249	207
R	14	8	I	87	48	A	129	88	I	169	128	F	210	168	G	250	208
S	15	9	T	88	49	V	130	89	A	170	129	N	211	169	I	251	209
L	16	10	A	89	50	L	131	90	T	171	130	S	212	170	S	252	210
T	17	11	M	90	51	D	132	91	V	172	131	I	213	171	S	253	211
L	18	12	I	91	52	V	133	92	P	173	132	T	214	172	A	254	212
I	19	13	P	92	53	L	134	93	T	174	133	D	215	173	I	255	213
N	20	14	D	93	54	N	135	94	E	175	134	Y	216	174	T	256	214
W	21	15	L	94	55	S	136	95	V	176	135	H	217	175	R	257	215
N	22	16	T	95	56	K	137	96	L	177	136	S	218	176	S	258	216
G	23	17	L	96	57	H	138	97	T	178	137	V	219	177	L	259	217
F	24	18	L	97	58	Q	139	98	E	179	138	L	220	178	R	260	218
F	25	19	H	98	59	R	140	99	I	180	139	F	221	179	D	261	219
A	26	20	F	99	60	V	141	100	V	181	140	E	222	180	Y	262	220
R	27	21	R	100	61	I	142	101	D	182	141	L	223	181	L	263	221
T	28	22	N	101	62	V	143	102	G	183	142	G	224	182	L	264	222
F	29	23	T	102	63	G	144	103	R	184	143	V	225	183	P	265	223
D	30	24	T	103	64	V	145	104	Q	185	144	L	226	184	E	266	224
L	31	25	E	104	65	R	146	105	A	186	145	P	227	185	N	267	225
D	32	26	A	105	66	L	147	106	K	187	146	K	228	186	S	268	226
E	33	27	G	106	67	Q	148	107	V	188	147	R	229	187	G	269	227
L	45	28	A	107	68	Q	149	108	I	189	148	L	230	188			
V	60	29	T	108	69	V	150	109	A	190	149	R	231	189			
T	61	30	T	109	70	A	151	110	L	191	150	S	232	190			
T	70	31	G	110	71	G	152	111	N	193	151	A	233	191			
L	71	32	S	111	72	R	153	112	E	194	152	S	234	192			
S	72	33	R	112	73	D	154	113	L	195	153	D	235	193			
G	73	34	D	113	74	K	155	114	K	196	154	R	236	194			
G	74	35	K	114	75	K	156	115	E	197	155	S	237	195			
N	75	36	G	115	76	V	157	116	R	198	156	K	238	196			
G	76	37	L	116	77	D	158	117	L	199	157	Y	239	197			
A	77	38	H	117	78	I	159	118	E	200	158	Y	240	198			
G	78	39	G	118	79	K	160	119	Q	201	159	R	241	199			
K	79	40	K	119	80	P	161	120	Q	202	160	L	242	200			

Table 12: **Alignment vs PDB residue mapping for the MukB domain set of 54 sequences.**
See legend Table 4-Text S15.

MukB 49 sequences																	
Res	Al pos	PDB	Res	Al pos	PDB	Res	Al pos	PDB	Res	Al pos	PDB	Res	Al pos	PDB	Res	Al pos	PDB
M	3	1	S	43	41	L	83	81	F	123	121	E	163	161	I	203	201
I	4	2	T	44	42	K	84	82	A	124	122	G	164	162	E	204	202
E	5	3	T	45	43	A	85	83	I	125	123	V	165	163	A	205	203
R	6	4	M	46	44	G	86	84	Q	126	124	Q	166	164	S	206	204
G	7	5	A	47	45	V	87	85	G	127	125	F	167	165	L	207	205
K	8	6	A	48	46	C	88	86	L	128	126	K	168	166	Y	208	206
F	9	7	F	49	47	Y	89	87	P	129	127	Q	169	167	G	209	207
R	10	8	V	50	48	S	90	88	M	130	128	F	170	168	G	210	208
S	11	9	T	51	49	M	91	89	S	131	129	N	171	169	I	211	209
L	12	10	A	52	50	L	92	90	V	132	130	S	172	170	S	212	210
T	13	11	L	53	51	D	93	91	Q	133	131	I	173	171	S	213	211
L	14	12	I	54	52	T	94	92	P	134	132	T	174	172	A	214	212
I	15	13	P	55	53	I	95	93	T	135	133	D	175	173	I	215	213
N	16	14	D	56	54	N	96	94	Q	136	134	Y	176	174	T	216	214
W	17	15	L	57	55	S	97	95	L	137	135	H	177	175	R	217	215
N	18	16	T	58	56	H	98	96	V	138	136	S	178	176	S	218	216
G	19	17	L	59	57	H	99	97	T	139	137	L	179	177	L	219	217
F	20	18	L	60	58	Q	100	98	E	140	138	M	180	178	R	220	218
F	21	19	H	61	59	R	101	99	T	141	139	F	181	179	D	221	219
A	22	20	F	62	60	V	102	100	L	142	140	D	182	180	Y	222	220
R	23	21	R	63	61	V	103	101	N	143	141	L	183	181	L	223	221
T	24	22	N	64	62	V	104	102	E	144	142	G	184	182	L	224	222
F	25	23	T	65	63	G	105	103	R	145	143	I	185	183	P	225	223
D	26	24	T	66	64	V	106	104	Q	146	144	I	186	184	E	226	224
L	27	25	E	67	65	R	107	105	A	147	145	A	187	185	N	227	225
D	28	26	A	68	66	L	108	106	R	148	146	R	188	186	S	228	226
E	29	27	G	69	67	Q	109	107	V	149	147	R	189	187	G	229	227
L	30	28	A	70	68	Q	110	108	L	150	148	L	190	188			
V	31	29	T	71	69	V	111	109	P	151	149	R	191	189			
T	32	30	S	72	70	A	112	110	L	152	150	S	192	190			
T	33	31	G	73	71	G	113	111	N	153	151	A	193	191			
L	34	32	S	74	72	R	114	112	E	154	152	S	194	192			
S	35	33	R	75	73	D	115	113	L	155	153	D	195	193			
G	36	34	D	76	74	R	116	114	K	156	154	R	196	194			
G	37	35	K	77	75	K	117	115	D	157	155	S	197	195			
N	38	36	G	78	76	V	118	116	K	158	156	K	198	196			
G	39	37	L	79	77	D	119	117	L	159	157	F	199	197			
A	40	38	H	80	78	I	120	118	E	160	158	Y	200	198			
G	41	39	G	81	79	K	121	119	A	161	159	R	201	199			
K	42	40	K	82	80	P	122	120	M	162	160	L	202	200			

Table 13: **Alignment vs PDB residue mapping for the MukB domain set of 49 sequences.**
See legend Table 4-Text S15.
