# Supplementary material for: Protein Fragments: Functional and Structural Roles of Their Coevolution Networks
Source: PLoS One. 2012 Nov 5;7(11):e48124. doi: 10.1371/journal.pone.0048124 (PMC3489791; doi:10.1371/journal.pone.0048124)
Supplement: Text S16 — BIS and CTMP comparison. (PDF) [file pone.0048124.s018.pdf]

Supporting Information Text S16: BIS and CTMP comparison

Protein family	Method	Co-evolving pairs
MukB (49 seq)	BIS, *pos*, $d = 0$	C1: 4 6 9<br>C2: 14 15 16 17 18 19 20 22 23 26<br>C3: **36 37 39 40 41** 42
	BIS, *pos*, $d = 1$	C1: 44 45 52 53 54 57 58 60 61 62 63 64 65 66 67 72 73 74 76 77 79<br>80 81 84 86 87 94 95 99 106 108 112 113 115 116 121 175 179<br>182 189 193 194 198 200 202 203 204 205 206 207 208 209 210 213 216<br>217 218 220 221 222 223 225 227<br>C2: 98 174<br>C3:107 111 117 214 219<br>C4:119 125<br>C5: 59 133<br>C6: 55 187<br>C7: 29 30 31 32 33
	CTMP	X:8 **36**:96 **14**:123 83:139 11:**86** 3:135 159:180 149:171 **29**:**32** **55**:180 50:82<br>128:211 **74**:191 **57**:129 X:**54** 75:226 **17**:X **59**:129 21:X 68:181 128:**208** X:89<br>**22**:X **26**:78 **41**:**64** X:**45** 134:**210** 110:226 **29**:**198** **18**:X 70:166<br>X:**198** X:141 **17**:X 109:191 X:173 154:**202** 7:**133** **72**:191 13:100 129:145 135:150<br>**30**:**76** X:136 **29**:**205** X:92 **14**:**30** **39**:150 **73**:135
MukB (54 seq)	BIS, *pos*, $d = 0$	C1: 16 20 22<br>C2: **36 37 39 40**
	BIS, *pos*, $d = 1$	C1: 45 61 64 66 77 200 202 203<br>C2: 44 60 62 76 84 204 210 222<br>C3: 54 74 81 99 179 182 207 217 221<br>C4: 80 205<br>C5: 52 193 220<br>C6: 31 33
	CTMP	42:83 47:X 42:192 25:X **74**:142 117:139 X:209 X:**205** X:**205** 46:135 2:195 X:25 **64**:144<br>X:91 X:**77** 191:211 4:151 **64**:122 25:X 104:127 98:191 **20**:42 **39**:144 X:156 3:46 25:X<br>48:129 83:108 41:176 X:**220** 4:126 73:127 96:101 X:87 **34**:65 3:212 X:196 X:78 **80**:106<br>3:116 **31**:48 71:128 X:78 156:159 126:145 14:**37** X:91 X:91 38:144
MukB (200 seq)	BIS, *pos*, $d = 0$	C1: 4 6 9<br>C2: 14 15 16 17 18 19 20 22 23 26<br>C3: **34 35 36 37 39 40 41** 42
	BIS, *pos*, $d = 1$	C1:44 45 52 53 54 57 58 60<br>61 62 63 64 65 66 67 72 73 74 76 77 79 80 81 84 86 87 94 95 99<br>108 112 113 115 116 121 175 179 182 189 193 194 198 200 202<br>203 204 205 206 207 208 209 210 213 216 217 218 220 221 222 223 225 227<br>C2:98 174<br>C3:107 111 117 214 219<br>C4:119 125<br>C5:59 133<br>C6:49 145 170<br>C7:55 187<br>C8:29 30 31 32 33
	CTMP	NO CO-EVOLVING PAIRS
MukB (205 seq)	BIS, *pos*, $d = 0$	C1: 16 20 22<br>C2: **36 37 39 40**
	BIS, *pos*, $d = 1$	C1: 45 61 64 66 77 200 202 203<br>C2: 44 60 62 76 84 204 210 222<br>C3: 54 74 81 99 179 182 207 217 221<br>C4: 80 205<br>C5: 52 193 220<br>C6: 31 33
	CTMP	NO CO-EVOLVING PAIRS

Table 1: **MukB protein: comparing BIS to CTMP.**

Comparison of CTMP and BIS (run on alignment positions - *pos*) on the MukB family. The CTMP experiment was run with the following parameters: penalty = 0.1, threshold on the fraction on gapped sequences = 1, threshold on the fraction of conserved sequences = 1, threshold on the fraction of sequences covarying between overlapping states = 0.0, threshold on the likelihood = 6.0. The 50 highest pairs of co-evolving residues (pdb positions) are reported. Bolds highlight residues in common with BIS.

Protein family	Method	Co-evolving pairs
PABD (20 seq)	BIS, *pos*, $d = 0$	C1: 22 23 27 29<br>C2: 30 31 32 33 34 35 36 37 38 39 40 42 46 48 49<br>C3: 41 44<br>C4: 51 52
	BIS, *pos*, $d = 1$	C1: 11 13 14 15 18<br>C2: 16 17
	CTMP	3:**11** 6:**23** **11:15** **11:38** **30:35** 3:6 3:**35** **35:38** **11:18** **11:52** **15:35** 6:**30** **11:16**<br>28:**35** **18:35** **11:36** 6:**38** **35:52** 6:**15** **11:49** **11:34** **16:35** **11:27** **11:51** **11:13**<br>**11:31** **35:49** **11:29** **11:22** **34:35** **35:36** **27:35** **11**:12 6:**18** **13:35** **29:35** **35:51**<br>**22:35** 12:**35** 6:**52** 6:**16** **11**:28 **31:35** 6:**49** 6:**36** 6:**34** **11:32** 6:**27** 6:**13**
PABD (28 seq)	BIS, *pos*, $d = 0$	C1: 32 39<br>C2: 42 49
	BIS, *pos*, $d = 1$	C1: 11 13 14
	CTMP	25:30 37:48 5:**13** **13**:28 25:51 **14**:33 30:33 5:**32** 43:46 8:34 24:**32** 16:25 8:20<br>37:50 21:37 **32**:38 37:45 17:31 8:38 **13**:35 **14**:18 8:19 19:**32** 15:17 8:28 20:**32**<br>**32**:44 28:**32** 31:54 17:51 25:43 15:54 31:33 8:44 15:33 40:54 17:29 **13**:46 17:30<br>8:26 23:25 25:38 **32**:50 33:40 25:34 17:36 3:37 30:40 29:54
PABD (452 seq)	BIS, *pos*, $d = 0$	C1: 22 23 27 29<br>C2: 30 31 32 33 34 35 36 37 38 39 40 42 46 48 49<br>C3: 41 44<br>C4: 51 52
	BIS, *pos*, $d = 1$	C1: 11 13 14 15 18<br>C2: 16 17
	CTMP	**35:42** **27:39** 28:**37** 21:47 8:**30** **16:39** 9:**35** 9:21 X:**34** 12:**52** 3:**39** 20:21<br>10:**46** 8:28 21:**48** 12:47 **39:44** **39:42** 47:**52** 7:21 X:28 **14**:50 6:**37** 5:**52** **17:35**<br>7:10 5:**40** 26:**39** 10:**37** 26:50 7:**52** 21:45 7:47 12:**40** 7:12 **16**:50 **40**:47 **42**:50<br>**40:52** **35**:47 X:25 **33:39** 10:19 **18**:50 **36**:50 21:**52** 5:12 **38:39** **29:39**
PABD (490 seq)	BIS, *pos*, $d = 0$	C1: 32 39
	BIS, *pos*, $d = 1$	C1: 11 13 14
	CTMP	23:50 26:37 **11**:53 20:**39** 4:27 **14**:37 19:**39** 10:52 5:21 X:20 36:37 X:**14** 21:52<br>4:19 X:19 45:50 21:40 7:35 18:50 35:47 4:20 **39**:46 X:23 3:8 **39**:43 X:36 8:23<br>7:52 **39**:45 3:50 47:52 31:**39** 4:**14** 25:**39** 40:52 30:53 44:50 4:44 4:26 37:44 4:16<br>4:18 8:37 10:35 X:44 8:25 10:**39** 19:37 31:50

Table 2: **Protein A-B domain: comparing BIS to CTMP.**

Comparison of CTMP and BIS (run on alignment positions) analyses of the Protein A-B domain family. See legend in Table 1-Text S16.

AATpase family	Method	Co-evolving pairs
DEAD-box	BIS, pos, $d = 0$	C1: 34 73 75 76 256 257 259 318 319 709 797 804
	BIS, pos, $d = 1$	C1: 133 213<br>C2: 136 801
	CTMP	528:529 528:856 153:528 153:826 528:826 528:851 155:528 154:528 470:528 108:528 828:829 528:530 112:528 528:626 528:857 93:528 528:612 161:528 826:828 498:528 288:528 528:621 529:530 472:528 490:528 61:528 111:528 52:528 501:528 528:858 528:702 492:528 46:528 500:528 528:624 528:860 528:609 486:528 525:528 485:528 528:623 528:854 59:528 1:528 528:611 528:614 7:528 289:528 528:618
DEAH-RHA	BIS, pos, $d = 0$	C1: 33 73 75 76 78 129 140 256 257 260 261 266 319 699 745 791 798 801 808
	BIS, pos, $d = 1$	C1: 70 127<br>C2: 72 130 174 213 215 259 317 318 700 708711 797 800 803 804<br>C3: 134 554<br>C4: 138 170<br>C5: 750 834
	CTMP	171:672 83:171 171:**801** 171:175 83:672 325:814 814:860 83:818 161:171 171:**261** 171:216 171:342 175:672 171:748 83:175 814:823 171:267 36:790 171:**804** 171:558 79:171 171:835 171:701 790:814 202:860 83:161 171:312 83:89 672:**801** 83:**801** 36:89 566:814 36:90 89:790 325:790 814:858 672:790 171:695 171:805 83:748 34:171 672:748 89:672 36:325 558:672 36:860 36:163 141:171 **33**:171
NS3/NPH-II	BIS, pos, $d = 0$	C1: 22 37 122 128 211 264 265 312 342 346 551 813 838<br>C2: 124 711<br>C3: 73 75 76 129 170 213 256 257 259 318 319 568 745 797 800 801 803 804<br>C4: 130 327<br>C5: 131 267<br>C6: 72 132 810<br>C7: 140 214 664 667<br>C8: 8 18 536 537 674<br>C9: 78 81 210<br>C10: 215 258 562<br>C11: 254 262<br>C12: 317 554<br>C13: 320 322 699 709 743<br>C14: 321 666<br>C15: 553 802<br>C16: 677 806<br>C17: 695 701
	BIS, pos, $d = 1$	C1: 125 698<br>C2: 136 217 271 657 751 862<br>C3: 16 137<br>C4: 139 831 844<br>C5: 20 23 328<br>C6: 28 763<br>C7: 333 746<br>C8: 39 794<br>C9: 544 661<br>C10: 79 759
	CTMP	1:2 1:3
Swi2/Snf2	BIS, pos, $d = 0$	C1: 73 75 76 77 136 256 257 320 804
	BIS, pos, $d = 1$	C1: 129 797 801 807<br>C2: 74 709
	CTMP	140:750 40:750 **73**:750 137:140 334:750 750:816 **74**:750 40:334 67:750 710:750 **75**:750 **256**:750 140:215 40:140 **73**:**136** 34:40 393:750 **136**:750 641:750 **74**:**75** 72:750 40:393 750:806 34:393 40:170 40:747 40:**256** 40:**73** 393:433 40:337 **73**:**74** 750:817 712:750 215:750 750:**801** 671:750 140:393 **73**:**75** 34:750 67:140 396:750 137:215 328:750 86:393 **73**:140 **74**:710 747:750 **709**:750 **75**:710

Table 3: **AATPase subfamilies: comparing BIS to CTMP.**

Comparison of CTMP and BIS (run on alignment positions) analyses of the AATPase subfamilies. BIS was run on the 11 subfamilies but only 4 of them output a list of residues. See legend in Table 1-Text S16. Note that these are the only AATPase subfamilies where CTMP provided some answer.
