# Supplementary material for: Protein Fragments: Functional and Structural Roles of Their Coevolution Networks
Source: PLoS One. 2012 Nov 5;7(11):e48124. doi: 10.1371/journal.pone.0048124 (PMC3489791; doi:10.1371/journal.pone.0048124)
Supplement: Text S17 — Protein A-B domain supplementary figures. (PDF) [file pone.0048124.s019.pdf]

Supporting Information Text S17:
Protein A-B domain supplementary figures

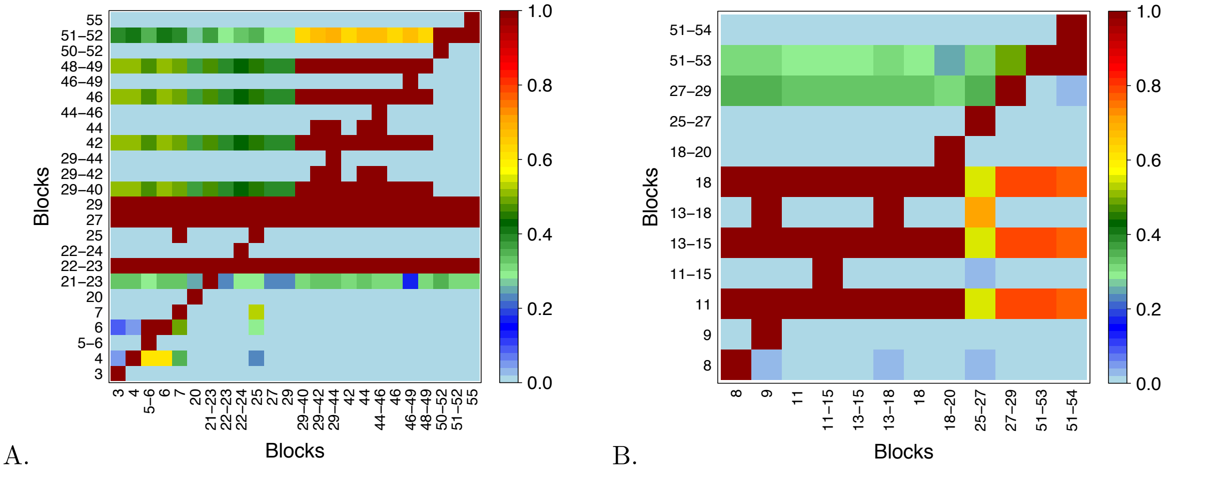

Figure 1: **Protein A-B domain: BIS unclustered correlated distribution score matrices.** BIS analysis realized on the dataset of 452 sequences. **A**: matrix computed for $d = 0$. The clustered matrix is reported in Fig. 2A-Text S17 (clusters have symmetric and environmental scores ≥ 0). **B**: matrix computed for $d = 1$.

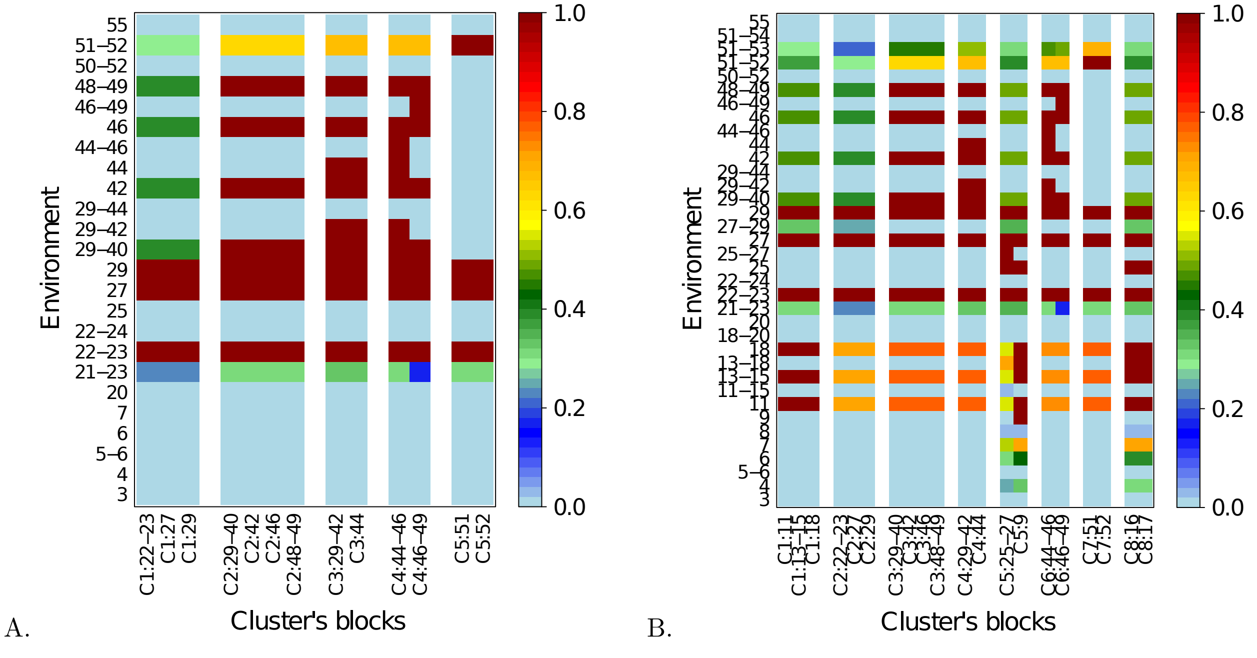

Figure 2: **Protein A-B domain: matrices computed for $d = 0$ and $d \leq 1$.** BIS analysis realized on the dataset of 452 sequences. Clustered correlated distribution score matrices for $d = 0$ (**A**) and $d \leq 1$ (**B**), where symmetric and environmental scores used for selecting clusters are ≥ 0. Notice that Fig. 4A is made of clusters of this matrix with scores $= 1$.
